# A Versatile and Sustainable Multicomponent Platform for the Synthesis of Protein Degraders: Proof-of-Concept Application to BRD4-Degrading PROTACs

**DOI:** 10.1021/acs.jmedchem.2c01218

**Published:** 2022-11-02

**Authors:** Irene Preet Bhela, Alice Ranza, Federica Carolina Balestrero, Marta Serafini, Silvio Aprile, Rita Maria Concetta Di Martino, Fabrizio Condorelli, Tracey Pirali

**Affiliations:** Department of Pharmaceutical Sciences, Università degli Studi del Piemonte Orientale, Largo Donegani 2, 28100 Novara, Italy

## Abstract

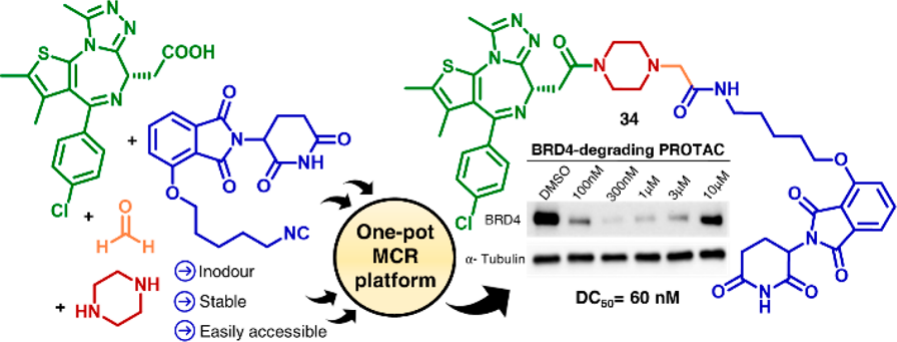

The use of small molecules to induce targeted protein degradation is increasingly growing in the drug discovery landscape, and protein degraders have progressed rapidly through the pipelines. Despite the advances made so far, their synthesis still represents a significant burden and new approaches are highly demanded. Herein we report an unprecedented platform that leverages the modular nature of both multicomponent reactions and degraders to enable the preparation of highly decorated PROTACs. As a proof of principle, our platform has been applied to the preparation of potential BRD4-degrading PROTACs, resulting in the discovery of a set of degraders endowed with high degradation efficiency. Compared to the existing methods, our approach offers a versatile and cost-effective means to access libraries of protein degraders and increase the chance of identifying successful candidates.

## Introduction

**Figure 1:**
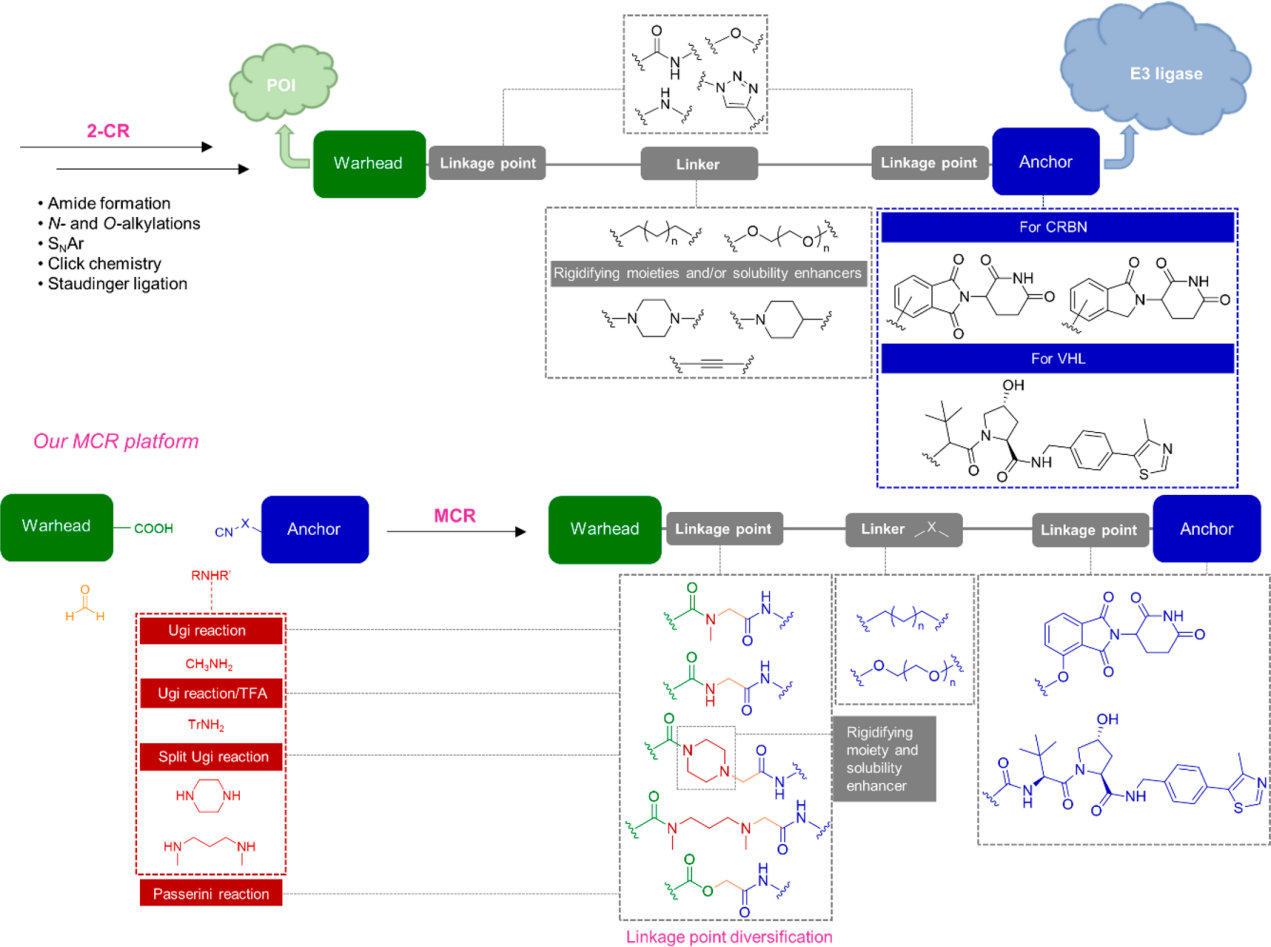
Structural components of PROTACs. Schematic illustration of their two-component reaction (2-CR)-based synthesis and of our multicomponent reaction (MCR)-based platform.

Protein degraders represent an attractive tool to control levels of disease-related proteins and are set to revolutionize drug discovery.^1,2^ Among them, PROTACs (Proteolysis TArgeting Chimeras) have drawn attention as one of the most appealing approaches.^3^ Being composed of a ligand for the protein of interest (POI) (referred to as warhead) and a recruiting moiety for the E3 ubiquitin ligase (referred to as anchor) connected through a suitable linker, PROTACs are able to hijack the ubiquitin–proteasome cascade and force the degradation of the POI (Figure 1). They effectively act as catalysts, being available for successive rounds of degradation upon completion of their cycle, thus minimizing the need for a continuous drug administration.^4^ Around 100 different proteins have been targeted so far by applying the PROTAC technology, which also proved to be an effective modality for accessing the “undruggable genome”.^5^ However, a grand total of more than 1000 proteins might be considered as PROTACtable, offering opportunities for future efforts based on protein degraders.^6^ Compared to the *occupancy-driven* approaches, PROTACs utilize an *event-driven* mechanism of action, which allows the knock down of disease-related POIs, offering an extraordinary strategy to overcome issues often experienced in classic drug discovery approaches, including resistance mechanisms (e.g., target protein overexpression and resistance mutations).^5,7−9^

**Figure 2:**
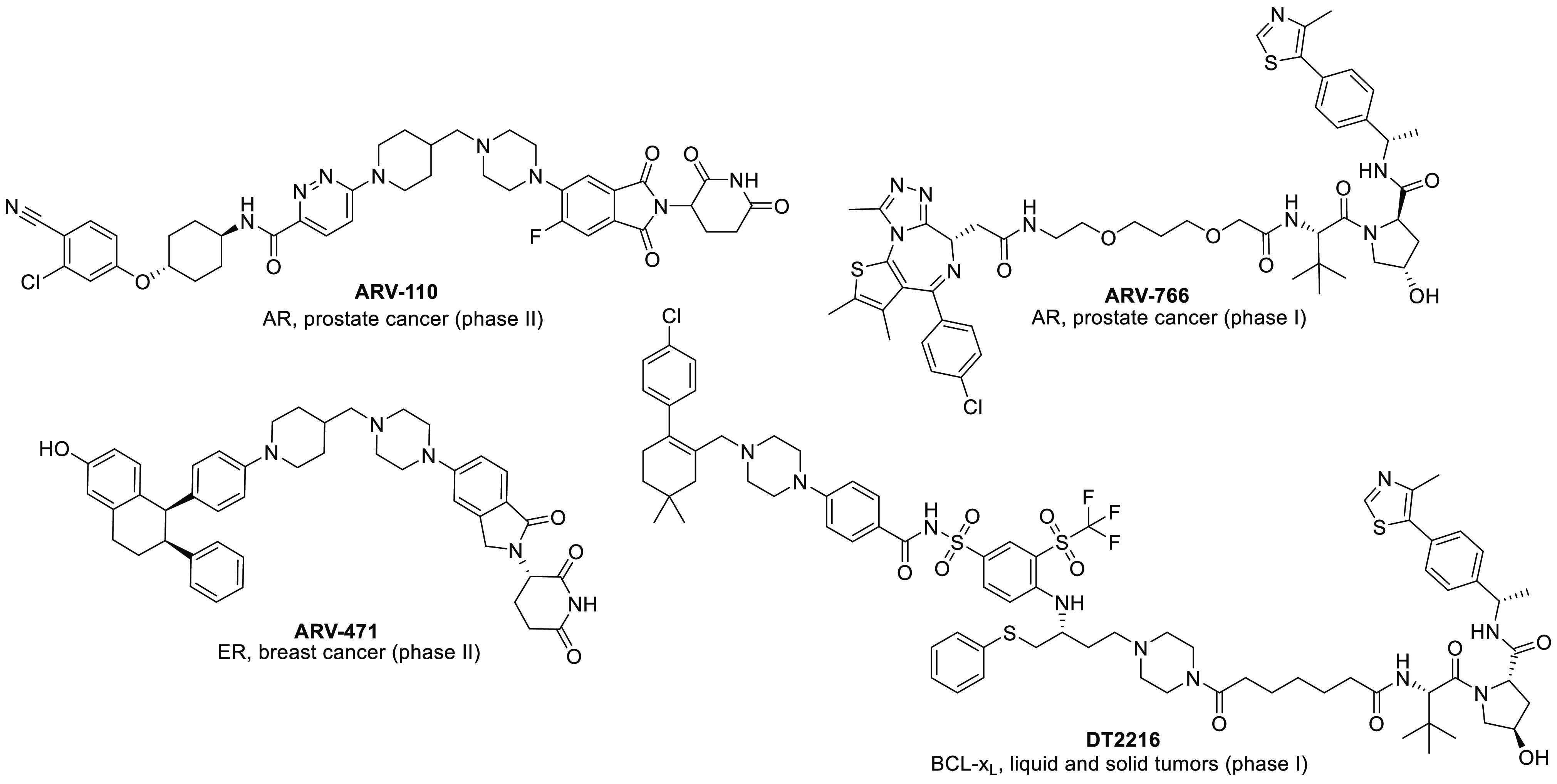
Disclosed structures of PROTACs advanced into clinical investigations. Androgen receptor (AR); estrogen receptor (ER); B-cell lymphoma-extra large (BCL-x_L_).

The great potential of the approach, which has grown exponentially in the past few years, has been confirmed by the first degraders that have reached clinical trials, including Arvinas degraders ARV-110, ARV-470, and ARV-766,^10,11^ and DT2216 developed by Dialectic Therapeutic, whose structures have been recently disclosed (Figure 2).^8,10^

**Figure 3:**
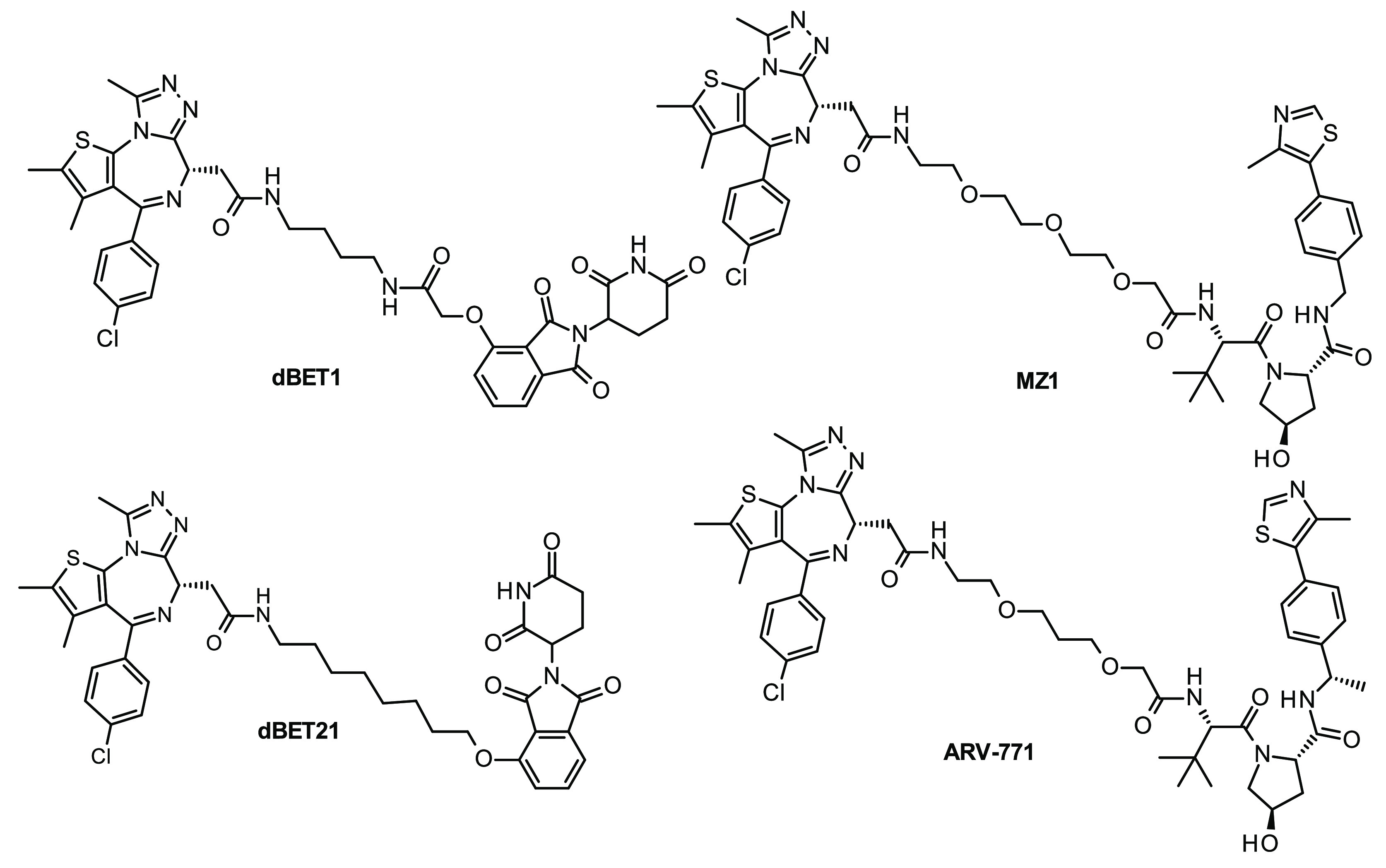
Structures of representative BET-degrading PROTACs based on (+)-JQ1 as a warhead.

While the POIs targeted by PROTACs currently under clinical investigation show a high degree of heterogeneity, bromodomain and extraterminal domain (BET) proteins have emerged as a model POI in many pilot studies probing PROTACs. BET proteins include BRD2, BRD3, BRD4, and BRDT and recruit transcriptional complexes by binding to acetylated lysine residues on histones, thereby controlling genes involved in cellular proliferation and cell cycle progression. Because alterations in the regulation of activities by BET proteins, especially BRD4,^12^ are associated with different inflammatory diseases and cancer, several BET degraders, including dBET1,^13^ MZ1,^14^ dBET21,^15,16^ and ARV-771^17^ (Figure 3) bearing (+)-JQ1^18,19^ as a warhead, have been developed, with the ultimate goal of finding more effective treatments compared to small-molecule inhibitors.^18−20^

From a structural point of view, while a large variety of warheads have been explored to bind different POIs, independently from their mechanism of action, cereblon (CRBN) targeting ligands (i.e., immunomodulatory imide drug (IMiD)-based ligands, such as thalidomide and its structural analogues)^21^ and Von Hippel-Lindau (VHL) targeting ligands (i.e., hydroxyproline-based molecules) are the most commonly used anchors to recruit E3 ligases (Figure 1).^4^ In contrast with the limited room of maneuver on both warhead and anchor, the linker and the linkage points represent important sites of diversification and are the focus of several medicinal chemistry programs currently. Linkers of different length and chemical nature (i.e., linear aliphatic and polyethylene glycol (PEG) chains and extended glycol chains) have been investigated with the main goal to modulate the degradation efficiency induced by PROTACs. On the same note, diverse linkage points of the linker to both the anchor and the warhead have been explored so far and their chemical nature is mainly driven by the chemical feasibility of PROTACs synthesis rather than by a rational choice (i.e., amine, ether, amide moieties, Figure 1). The sites of attachment on the anchor and the warhead are critical and are usually selected by analyzing the solvent-exposed regions, to minimize the interference of the linker with the binding of the PROTAC to the E3 ligase and the POI, respectively (Figure 1).

An optimal combination of all different PROTAC structural elements is of pivotal importance in triggering the productive formation of the ternary E3 ligase–PROTAC–POI complex and the following proteasome cascade activation.^22,23^ The rational design of effective PROTACs, which require cooperativity in assembling the ternary complex (TC) and establishing adventitious favorable interactions between the POI and the E3 ligase, is still a key challenge. However, progress in structural biology, X-ray crystallography, molecular modeling, and dynamics simulations has been recently made to rationalize the formation and stability of PROTAC-mediated TC and to understand the PROTAC mechanism of action.^19,24−26^

Besides degradation efficiency, drug-like properties such as aqueous solubility, metabolic stability, and cell permeability represent additional challenges to face for achieving in vivo efficacy, with far more reason because PROTACs lie outside the rule-of-five space.^27−29^ Hence, massive efforts have been devoted to rationally explore the role of the different structural elements in conferring favorable in vitro ADME properties.^30^ Because it has become increasingly clear that the length and chemical nature of the linker influence both the bioactivity and physicochemical properties of PROTACs, a shift from synthetically tractable linear aliphatic and PEG-based chains to more sophisticated and rigid motifs (e.g., piperazines, piperidines) is currently ongoing.^27,31,32^

Despite the advances made so far, the rational conversion of a ligand for the POI into an effective degrader in vivo remains rather empirical and based on a “trial and error” approach.^33^ Furthermore, the lack of reliable and economically sustainable synthetic methods represents an important caveat that necessarily limits the number of accessible compounds. The assemble of PROTACs has proven to be far from straightforward, as it involves the asymmetric diversification of the two sides of the linker and their chemoselective reactions with the two protein binding motifs. This challenge is usually achieved through orthogonal conditions or protection/deprotection sequences. Linking strategies to couple the linker with the warhead and the anchor include amide bond formation, N- and O-alkylations, nucleophilic aromatic substitution (S_N_Ar), acylation, and Williamson ether synthesis, transformations that are known to suffer from poor reactivity, low chemoselectivity, and lack of atom economy (Figure 1).^34^ Thus, the preparation of PROTACs often requires low-yielding and cumbersome multistep synthetic routes, makes extensive use of protecting groups, and relies on highly functionalized and costly building blocks.^35^

To overcome these limitations, advances aimed at simplifying the access to protein degraders and their screening have been reported^32^ and include the use of orthogonally protected bifunctional linkers,^36^ solid-phase synthesis,^37^ click chemistry,^38^ Staudinger ligation,^39^ a direct-to-biology (D2B) approach,^40^ and others.^41,42^ Nevertheless, none of these strategies has established itself as the ideal method so far, due to existing limitations. For instance, among the recent methods reported to ease the preparation of PROTACs, the click chemistry platform allows the preparation of both CRBN- and VHL-recruiting degraders, but the introduction of the triazole ring is well-known for leading to poorly soluble products and for its reluctance to scale-up.^43^ In an effort to streamline the discovery of successful PROTACs and expand the chemical space explored so far, we report a modular synthetic platform that capitalizes on the versatility of multicomponent reactions (MCRs) to assemble heterobifunctional protein degraders (Figure 1).

## Results and Discussion

### Selection of Synthons for the Preparation of PROTACs via MCR Platform

**Scheme 1:**
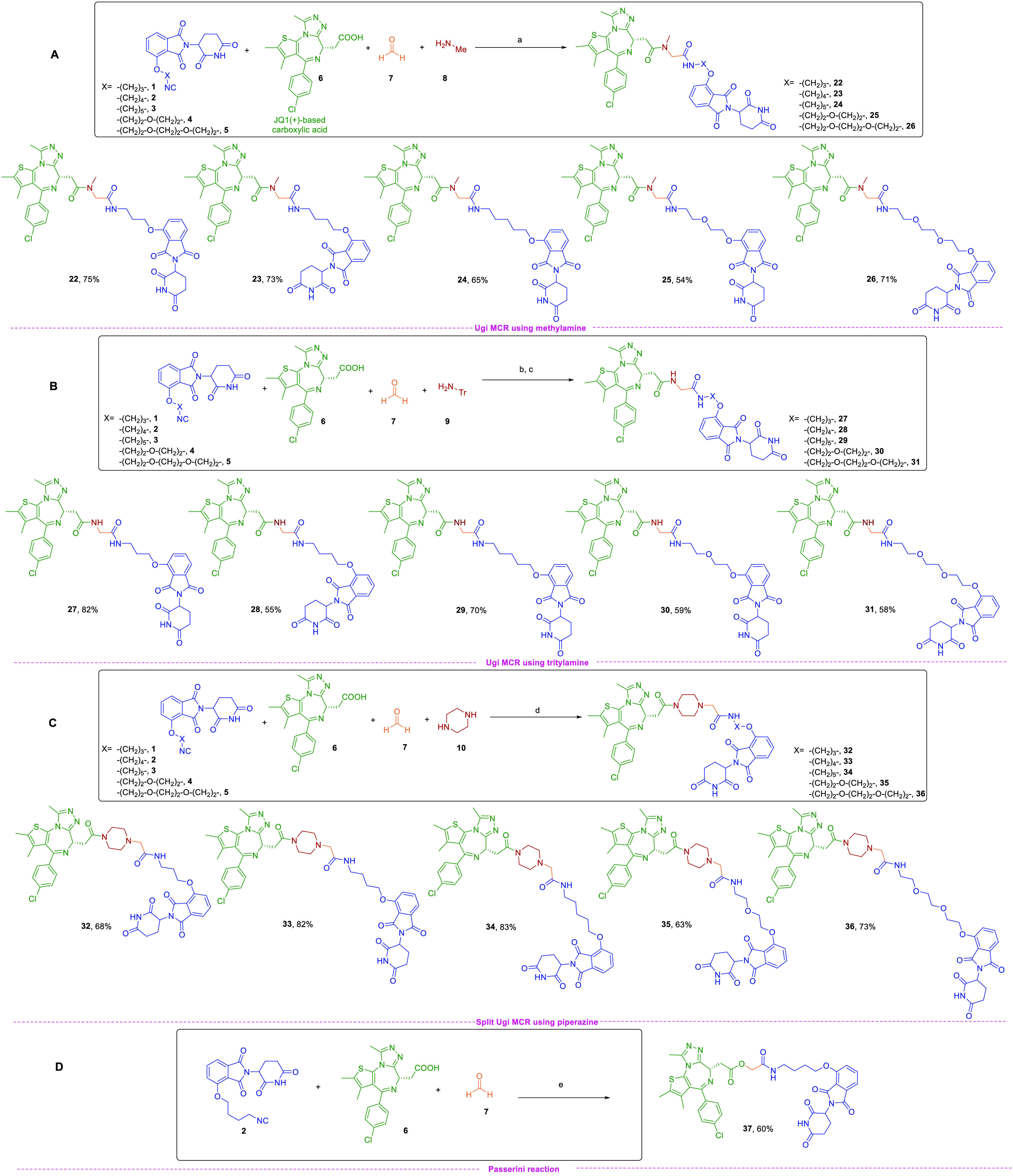
Library of PROTACs Synthesized by Exploiting Ugi/Split Ugi and Passerini MCRs Reagents and conditions: (a) MeOH, 0 °C, 2 h, then rt, 1 h; (b) MeOH, 40 °C, 1 h, then 24 h rt; (c) TFA, CH_2_Cl_2,_ 0 °C, 30 min, then rt, 3 h; (d) MeOH, reflux, 2 h; (e) CH_2_Cl_2,_ 40 °C, 4 h. Color code does not refer to the MCR mechanism but is intended to represent the synthons and their assembly into the protein degraders.

In designing our platform we initially focused on Ugi^44,45^ and Passerini^46^ reactions, as these MCRs stand out for their efficiency, versatility, high atom economy, and simple and environmentally friendly experimental procedures, where the only byproduct, when present, is one water molecule per molecule of product. As a proof of concept, we decided to use the following synthons as a model system: five different isocyanides bearing chains of different length and the thalidomide-based CRBN-recruiting anchor (**1**–**5**), a carboxylic acid (**6**) bearing the warhead based on (+)-JQ1,^19^ formaldehyde (**7**) as the carbonyl compound to minimize the interference of the linker with the binding to the POI, and three different commercially available amines (methylamine **8**, tritylamine **9**, and piperazine **10**). Scheme 1 provides an overview of the synthesized PROTACs by using the aforementioned synthons and by exploiting the reactions as discussed in further detail below.

### Preparation of Isocyanide-Based CRBN-Recruiting Anchors

**Scheme 2:**
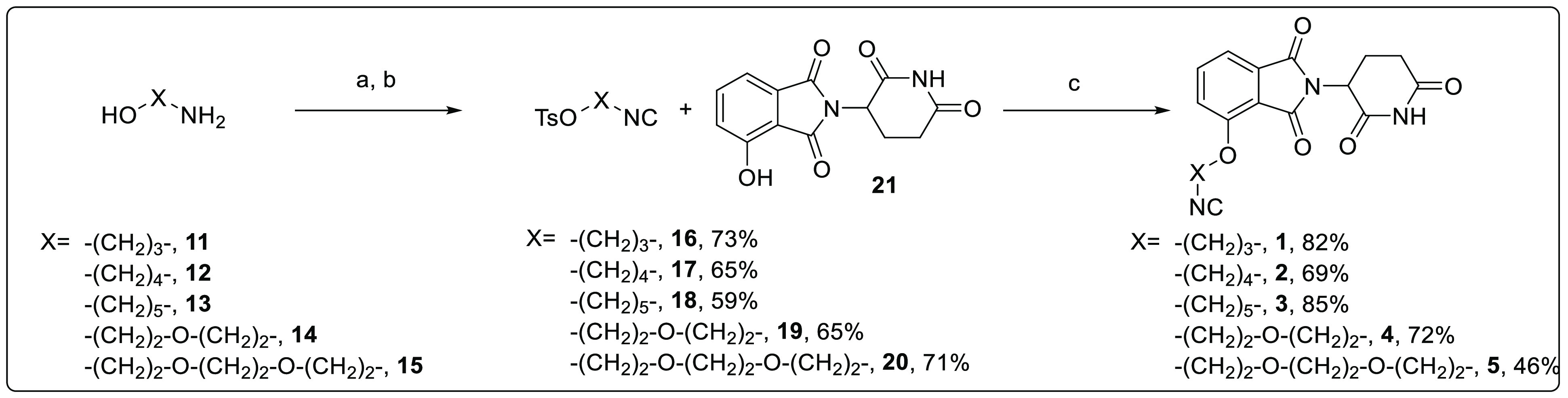
Synthetic Route To Access Isocyanide-Based CRBN-Recruiting Anchors **1**–**5** Reagents and conditions: (a) ethyl formate, reflux, 6 h; (b) TsCl, TEA, dry CH_2_Cl_2_, rt, 5 h; (c) NaHCO_3_, dry DMF, 65 °C, 24 h.

While (+)-JQ1-based carboxylic acid (**6**), formaldehyde (**7**), and all the selected amines (**8**, **9**, and **10**) are commercially available, we applied a sustainable multistep protocol as depicted in Scheme 2 to access the desired thalidomide-based CRBN-recruiting isocyanides (**1**–**5**). The first step is the formylation of the proper amino alcohol (**11**–**15**) by using ethyl formate, followed by evaporation of the formylating agent. Further treatment of the *N*-formamide intermediate with tosyl chloride in the presence of triethylamine as base allowed us to obtain the corresponding linker (**16**–**20**) bearing both the isocyanide and the tosylate functionalities in 59–73% yield. According to a recent report by Meier et al. published in *Green Chemistry*, tosyl chloride represents an efficient and practical alternative to toxic and hazardous dehydrating agents such as phosphorus oxychloride in the preparation of aliphatic isocyanides.^47^ This reagent proved to be suitable to our purpose, as it can simultaneously dehydrate the formamide group and convert the hydroxy function of **11**–**15** to an excellent leaving group. In the last step, an O-alkylation reaction takes place between derivatives **16**–**20** and 4-hydroxythalidomide (**21**) in the presence of sodium bicarbonate to afford both linear aliphatic (**1**–**3**) and PEG (**4** and **5**) linkers in yields ranging from 46% to 85%. Notably, our optimized and mild experimental conditions avoided both the N-alkylation at the secondary nitrogen atom and the opening of the phthalimide/glutarimide ring of **21**, two undesirable events that would result in the loss of E3 ligase recruitment.^48^ Furthermore, all isolated isocyanides proved not to suffer from the unpleasant smell typical of these synthons, due to the high molecular weight and solid state at room temperature, and showed stability upon long-term storage.^49^

### Exploration of Different Amines To Probe the Warhead Linkage Point

With all isocyanide-base linkers in our hands, we turned our efforts into the variation of the amine component. We began with methylamine (**8**) as a model for conventional primary amines and PROTACs **22**–**26** (Scheme 1A) synthesized in 54–75% yields. To further reduce steric hindrance at the attachment point and minimally influence affinity of the warhead for its target protein, we then explored the use of tritylamine (**9**), that our laboratory had reported as an effective surrogate of ammonia,^50^ especially when coupled with formaldehyde as the carbonyl component. The reaction was performed in methanol and, upon completion, the trityl group was cleaved by adding trifluoroacetic acid to give the desired PROTACs **27**–**31** in 55–82% yields (Scheme 1B). Finally, we carried out the split Ugi reaction, which we reported in 2006.^51^ In this variant of the Ugi reaction a secondary amine is used, instead of a primary one, leading to the acylation of one nitrogen atom, while the other one is alkylated. This transformation particularly suited our purposes, as it allowed the one-pot incorporation of the piperazine ring, a privileged substructure of several PROTACs, including ARV-110 and ARV-471 (Figure 2),^11,52^ that helps to improve solubility and metabolic stability^27^ and promotes the formation of stable TC.^53−55^ Noteworthy is the use of piperazine as the amine component, which allows an increase in the sp^3^ character and reduces the number of HBDs and HBAs that are associated with oral bioavailability improvement.^31^ Piperazine-based PROTACs **32**–**36** were successfully synthesized in yields of 63–83% (Scheme 1C).

Due to the well-known configurational instability that affects thalidomide substructures, all PROTACs were obtained as a mixture of diastereomers, which include a racemate at the IMiD stereocenter and an enantiopure (+)-configuration at the JQ1 stereocenter. To explore the scalability of our synthetic approach, the preparation of isocyanide **3** was performed on a 6.00 mmol scale and the split Ugi reaction was scaled up to 1.50 mmol to afford 1 g of **34** in 83% yield.

### Implementation of the Platform with the Passerini MCR

Very recently, Ciulli et al. have reported the bioisosteric replacement of an amide function with an ester at the linkage point between the warhead and the linker as a successful strategy to enhance cell permeability, without significantly affecting metabolic stability.^56^ This report encouraged us to include the Passerini reaction^57^ in our MCR platform. To this aim, we selected the isocyanide-bearing linker **2**, which reacted with (+)-JQ1-based carboxylic acid **6** and formaldehyde **7** in the absence of the amine component in dichloromethane to afford PROTAC **37** in 60% yield (Scheme 1D).

### Preparation of VHL-Recruiting PROTACs To Probe the Anchor

**Scheme 3:**
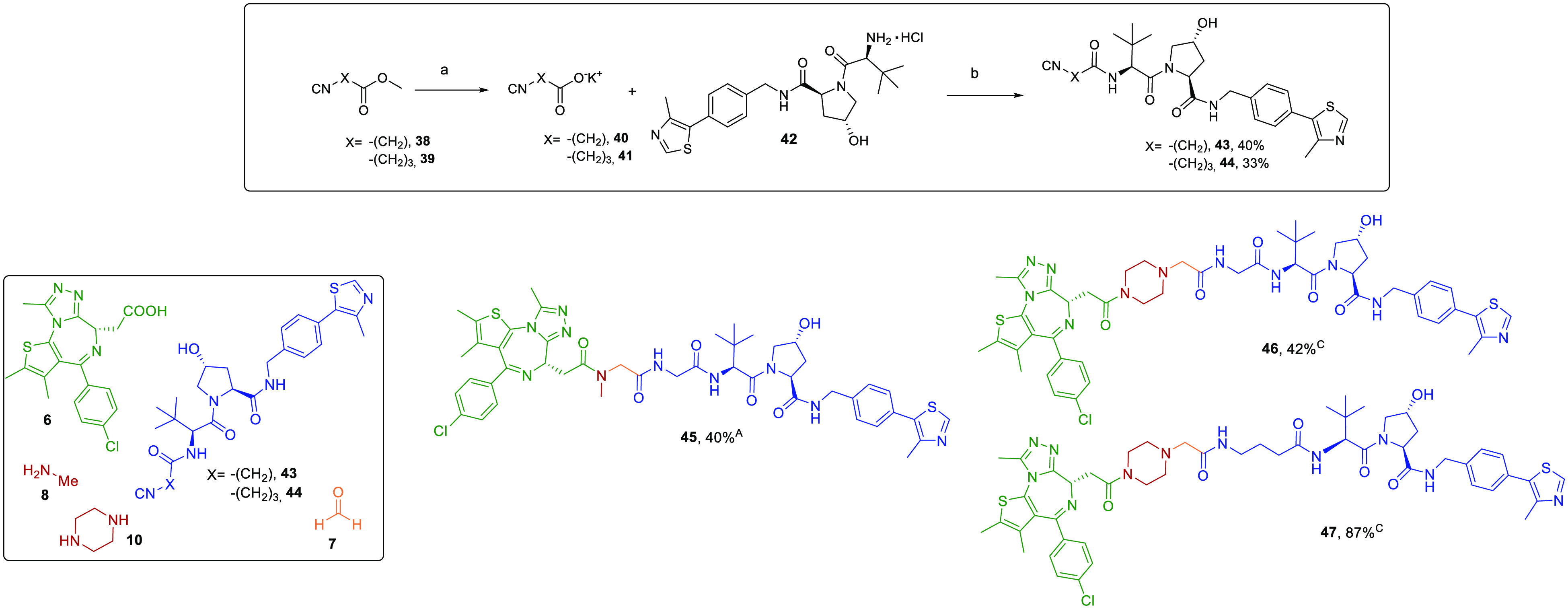
Synthesis of VHL-Recruiting Isocyanides **43** and **44** and PROTACs **45**–**47** Reagents and conditions: (a) KOH, MeOH, 0 °C to rt, 2 h and 30 min; (b) HATU, DIPEA, dry DMF, rt, 7 h. A and C refer to reaction conditions reported in Scheme 1A and 1C. Color code does not refer to the MCR mechanism but is intended to represent the synthons and their assembly into the protein degraders.

To further investigate the versatility of our approach, we synthesized the isocyanide-based linkers bearing the anchor for the VHL proteins **43** and **44** via a two-step procedure depicted in Scheme 3. Saponification of isocyanide methyl esters **38** and **39** gave the corresponding potassium salts **40** and **41**, which reacted with the commercially available hydrochloride amine **42** in the presence of HATU as coupling agent and DIPEA as base.

A final Ugi reaction using methylamine **8** and isocyanide **43** afforded VHL-recruiting PROTAC **45** in 40% yield, as well as two split Ugi reactions employing piperazine **10**, and isocyanides **43** and **44** gave **46** and **47**, respectively, in 42% and 87% yields (Scheme 3).

**In Vitro Biological Validation of PROTACs and Preliminary Assessment of Thermodynamic Aqueous Solubility and Metabolic Stability of Selected BRD4-Degrading PROTACs.** With the aim of proving the utility of our platform and the compatibility of the substructures accessible by the MCR strategy with a proximity-based degradation, the ability of our PROTAC candidates to effectively act as BRD4 degraders was tested in the human breast cancer cell line MDA-MB-231, known to express detectable levels of this protein. To this aim, BRD4 protein levels were assessed by Western blot analysis after exposing cells to each of the selected compounds. dBET1 PROTAC was used as the benchmark reference for comparison of CRBN-recruiting compounds, while (+)-JQ1 was used as negative control of protein degradation. Alternatively, MZ1 was identified as reference and cis-MZ1 as negative control for VHL-recruiting PROTAC candidates **45**–**47**.^13^

**Figure 4:**
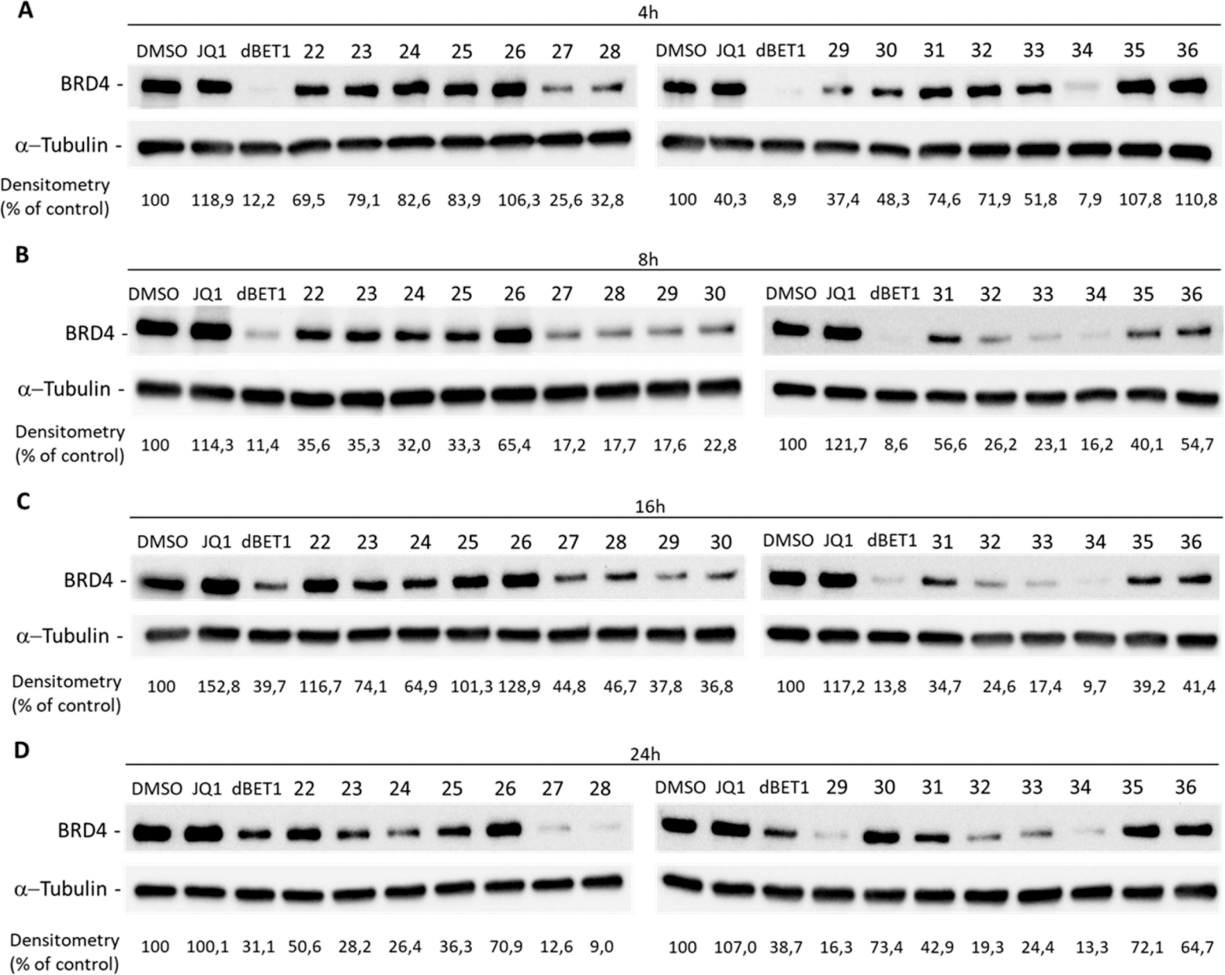
BRD4 degradation after treatment with CRBN-recruiting compounds. Western blot analysis of BRD4 degradation induced by CRBN-recruiting (A–D) compounds at the concentration of 1 μM in MDA-MB-231 cells after 4, 8, 16, and 24 h of treatment. Treatments with 1 μM of (+)-JQ1 or dBET1 were used as negative and positive controls for BRD4 degradation. α-Tubulin was used as internal control for equal loading. Degradation activity is reported below each lane as % of BRD4-immunoreactive band densitometry relative to DMSO vehicle. Western blot results shown in Figure 4 are representative of three (*n* = 3) independent experiments.

**Figure 5:**
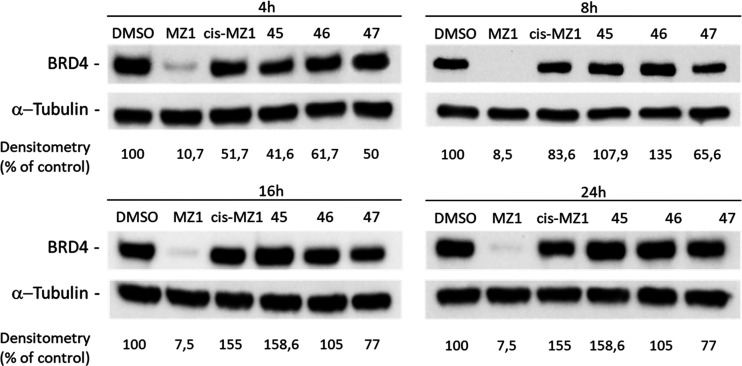
BRD4 degradation after treatment with VHL-recruiting compounds. Western blot analysis of BRD4 degradation induced by VHL-recruiting (E) compounds at the concentration of 1 μM in MDA-MB-231 cells after 4, 8, 16, and 24 h of treatment. Treatments with 1 μM of cis-MZ1 or MZ1 were used as negative and positive controls for BRD4 degradation. α-Tubulin was used as internal control for equal loading. Degradation activity is reported below each lane as % of BRD4-immunoreactive band densitometry relative to DMSO vehicle. Western blot results shown in Figure 5 are representative of three (*n* = 3) independent experiments.

To preliminarily identify which of these molecules showed effective BRD4 degradation, we ran time-course experiments (4 up to 24 h) using 1 μM concentration of each compound (Figures 4 and 5). As shown in Figure 4A–D, CRBN-recruiting compounds **27**, **28**, **29**, and **34** led to a decreased expression of BRD4 at all the time-points tested. Also compounds **32** and **33** were able to induce BRD4 degradation, although the onset of this effect was delayed (after 8 h). Conversely, compound **30** caused a modest and transitory degradation of BRD4 (at 8 and 16 h of treatment), as well as compound **31** (at 16 h of treatment), while none of the VHL-recruiting compounds (**45**–**47**), with the exception of MZ1, acted as a BRD4 degrader (Figure 5).

According to time-course experiments, compounds **27**, **28**, **29**, **32**, **33**, and **34** resulted in the most active degraders, albeit with different times of action. Therefore, these PROTACs were selected for further characterization of dose–response and proteasome-dependence of BRD4 degradation. To define whether their activity is dose-dependent, MDA-MB-231 cells were treated with increasing concentrations of each compound (from 100 nM up to 10 μM) for 8 h, because at this time-point they were found effective, as shown in Figure 4. By this approach, compounds **27**, **28**, and **32** showed dose-dependency, with an effect peaking between 1 and 10 μM concentrations. Conversely, compounds **29** and **33** displayed a narrower window of active concentrations because degradation was observed only at 1 and 3 μM concentrations (Figure 7). Notably, **34** was the most potent compound, was able to cause maximal degradation of BRD4 at 300 nM, and showed the well-known “hook effect”.^33^

**Figure 6:**
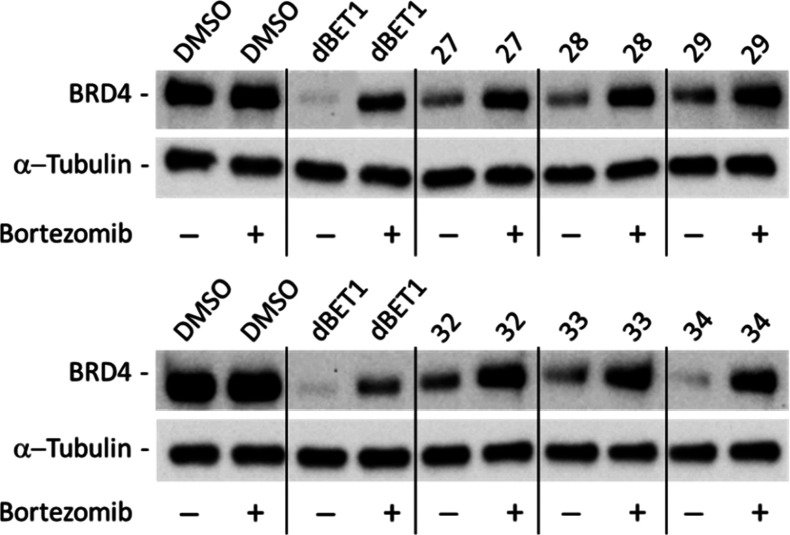
PROTAC activity is inhibited by the proteasome inhibitor bortezomib. BRD4 protein levels after 8 h treatments with DMSO (vehicle) or 1 μM concentrations of compounds **27**, **28**, **29**, **32**, **33**, and **34** alone or in combinations with 5 nM bortezomib. α-Tubulin was used as internal control for equal loading. Western blot results shown in Figure 6 are representative of three (*n* = 3) independent experiments.

To assess the dependency of BRD4 degradation on the proteasome machinery, we studied the effect of our best compounds in the presence of the proteasome inhibitor bortezomib. As shown in Figure 6, cotreatment with 5 nM bortezomib was able, after 8 h, to abolish BRD4 degradation induced by compounds **27**, **28**, **29**, **32**, **33**, and **34** (all used at the 1 μM concentration), demonstrating the specificity of their molecular activity.

**Figure 7:**
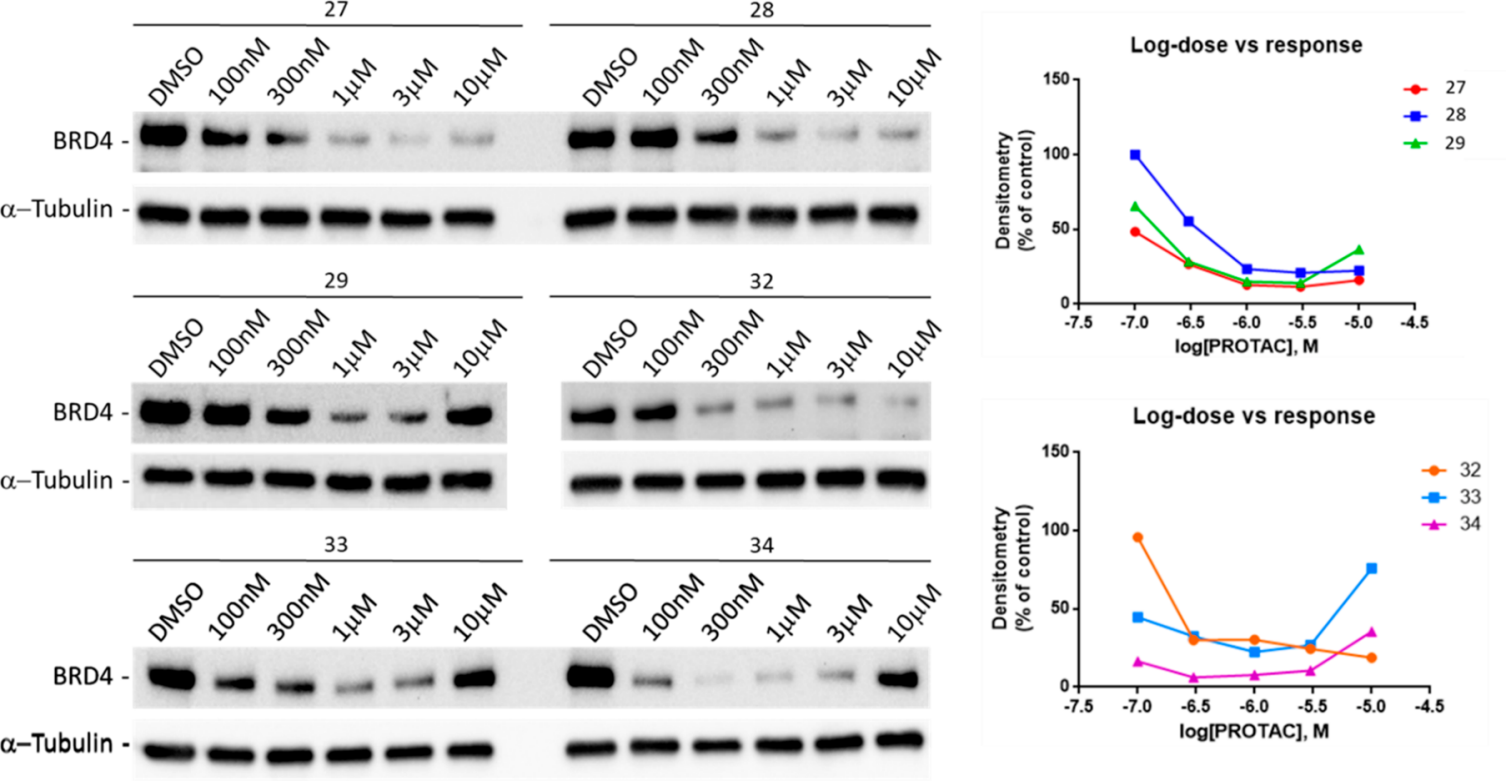
Dose–response of BRD4 degradation induced by PROTAC candidates. Western blot analysis (left) of BRD4 protein levels in MDA-MB-231 cells after 8 h treatments with increasing concentrations (100 nM to 10 μM) of compounds **27**, **28**, **29**, **32**, **33**, and **34** or DMSO (vehicle). α-Tubulin was used as internal control for equal loading. Results were normalized using densitometry of α-tubulin immunoreactive-bands as internal control for equal loading. Western blot shown in Figure 7 are representative of three (*n* = 3) independent experiments.

**Figure 8:**
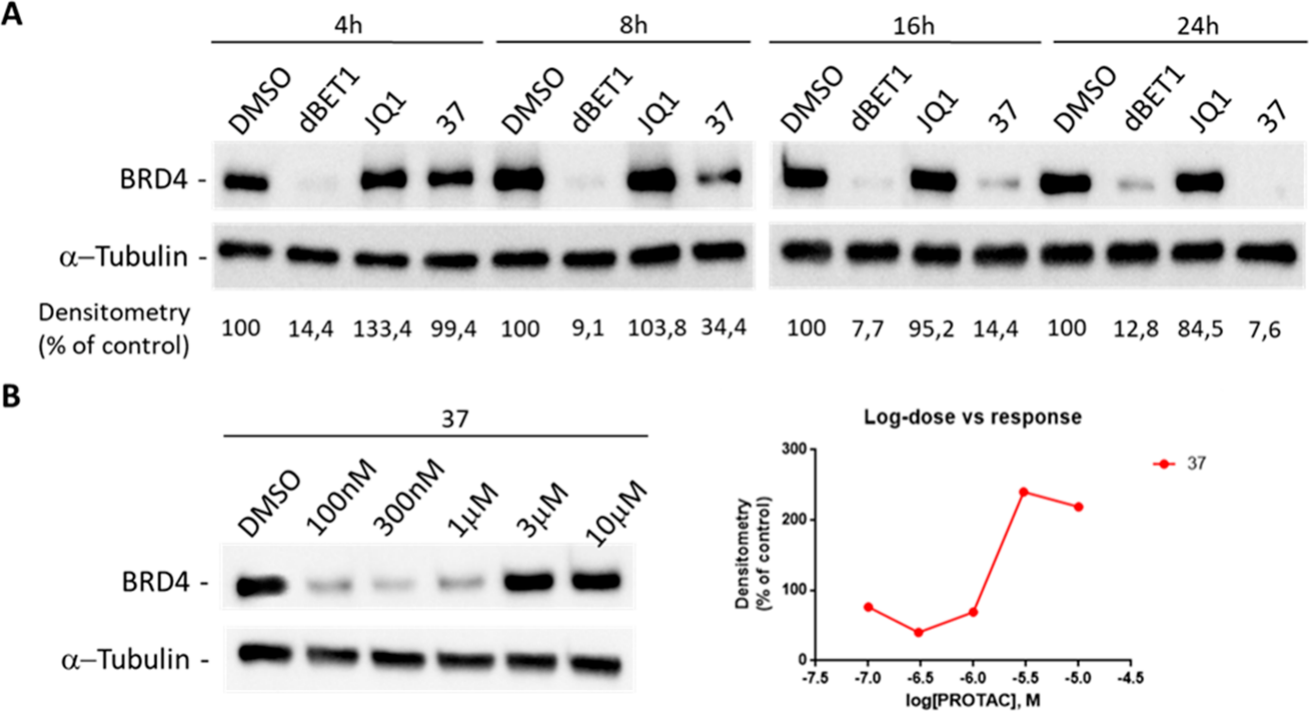
BRD4 degradation after treatment with compound **37**. Western blot analysis of BRD4 protein levels in MDA-MB-231 cells treated with 1 μM **37** for 4, 8, 16, and 24 h (A). Treatments with 1 μM (+)-JQ1 or dBET1 were used as negative and positive controls for BRD4 degradation (A). Western blot analysis (B) of BRD4 protein levels in MDA-MB-231 cells after 8 h treatments with increasing concentrations (100 nM to 10 μM) of compound **37** or DMSO (vehicle).

Of note, compound **37**, which was synthesized using the Passerini reaction and is characterized by a α-acyloxy amide instead of a bis-amide, caused degradation of BRD4, although this effect was evident only after 16 and 24 h of incubation at the 1 μM concentration (Figure 8A). Similarly to **34**, when dose–response degradation was investigated after 8 h of treatment, the maximal effect was seen at 300 nM (Figure 8B).

α-Tubulin was used as internal control for equal loading. Degradation activity is reported below each lane as % of BRD4-immunoreactive bands densitometry relative to DMSO vehicle. Western blot results shown in Figure 8 are representative of three (*n* = 3) independent experiments. DC_50_ (nM) and *D*_max_ (%) of PROTACs **27**–**29**, **32**–**34**, and **37** were calculated and are reported in Table 1.

**Table 1:** DC_50_ (nM) and *D*_max_ (%) Values of Compounds **27**–**29**, **32**–**34**, and **37** after 8 h Treatments^a^

compound	DC_50_ (nM)	*D*_max_ (%)
**27**	97.1	88
**28**	134.0	79
**29**	184.0	86
**32**	239.0	81
**33**	89.9	78
**34**	60.0	94
**37**	62.0	86

a Values were calculated using densitometry of Western blots shown in Figures 7 and 8B. *D*_max_ is expressed as maximal % of reduction toward DMSO control lane.

Overall, the SAR study suggests that, for the CRBN-targeting PROTACs, the substructure including the *N*-methyl-substituted tertiary amide (**22**–**26**) prevents in all cases BRD4 degradation, while the secondary bis-amide moiety as well as the acetyloxy amide favors the efficiency (**27**–**30**, **37**), especially when the total linker length is between 10 and 12 atoms, irrespective of its hydrocarbon (**27–29**, **37**) or PEG composition (**29**). Finally, PROTACs displaying the piperazine ring are effective when the length is in the range of 13 to 15 (**32**–**34**), while longer chains are not tolerated (**36**).

Having demonstrated that the introduction of additional HBAs and HBDs does not prevent cell membrane crossing and subsequent BRD4 degradation, we were curious to preliminarily assess the aqueous solubility of structures potentially accessible through our platform and, to this aim, we selected compounds **27**–**29** as representative of Ugi-like products with different lengths, **34** that exemplifies the split-Ugi products and **37** as the result of the Passerini reaction. Being aware of thalidomide hydrolytic degradation at physiological pH (about 30% degradation in 3 h),^27^ we determined the thermodynamic solubility for the selected compounds for 2 h to limit the permanence in aqueous media and monitored hydrolysis by high performance liquid chromatography (HPLC) analysis. At physiological pH, except for **27**, all tested PROTACs showed a poor solubility (<30 μM), which is comparable with the solubility of the majority of commercial PROTACs.^30^ As expected, the Passerini product **37** that has one less HBD compared to **27**–**29** was the least soluble. Under acidic conditions, the solubility significantly increased, especially for compound **34** that displays an ionizable piperazine ring, further confirming the utility of introducing this substructure in PROTAC design (Table 2).

**Table 2:** Preliminary Data of Thermodynamic Aqueous Solubility (μM) and Metabolic Stability (Residual Substrate %) of Selected PROTACs

compound	PBS 0.1 M (pH = 7.4)	HCl 0.01 N (pH = 2.0)	metabolic stability^a^^,^^b^ (%)
**27**	48	145	36
**28**	17	48	40
**29**	15	39	88
**34**	11	5073	60
**37**	8	18	89
**dBET1**	60^c^	171^c^	66

a Residual substrate after 1 h incubation in MLM in the presence of NADPH.

b The residual substrate in control incubations without MLMs was >99%.

c Literature data.^58^

Finally, we were interested in ruling out the possibility that additional amides and/or esters may represent potential soft spots with a negative impact on metabolic stability. To this aim, we evaluated the metabolic stability of the five selected PROTACs in mouse liver microsomes (MLM). Interestingly, metabolic biotransformation only occurred when the microsomal monooxygenase system was activated by NADPH, suggesting susceptibility to oxidative metabolism but not to hydrolysis.^27^ Contrary to what happened in phosphate saline buffer, no significant degradation of thalidomide was observed in control incubations carried out in Tris-HCl (see Experimental Section). Concerning the active compounds of the Ugi-like products series (**27**–**29**), the stability increased depending on the linker length: indeed, the residual substrate percentage after 1 h incubation raised from 36% for derivative **27** to 88% for **29**. Also PROTAC **37**, synthesized by the Passerini reaction, proved to be highly stable in MLM, in accordance with previous findings on PROTACs displaying esters in plasma (Table 2).^56^

## Conclusions

In this study, we present an unprecedented MCR-based platform that streamlines the synthetic entry into the chemical space of protein degraders. By varying the amine component in the MCR, the platform allows extensive structure–activity and structure–property relationship studies around not only the linker and its length and composition but also the linkage point to the warhead that strongly influences the affinity of the PROTAC for the POI and might be responsible for metabolic instability.^27,59^

When compared to the existing 2-CR methods, our approach stands out for being reliable, high yielding, versatile, protecting group-free, stereoconservative, and sustainable. While in this paper we have focused only on BRD4 degrading PROTACs, the platform might be used for targeting different POIs, provided that the warhead bearing a carboxylic group moiety is accessible. Our approach obviates the need to perform protection/deprotection steps typically required in the synthesis of PROTACs, except for the use of tritylamine as a surrogate of ammonia. An additional feature of this strategy is given by the well-known stereoconservative nature of Ugi and Passerini reactions that is fundamental when chiral warheads and anchors are involved in the assembly of the protein degrader.^60,61^ Finally, the process is characterized by a low environmental footprint. Except for the (+)-JQ1-based carboxylic acid, all the other substrates required for the preparation of both the linkers and the final degraders are inexpensive and easy to handle, in stark contrast with the cost associated with most of the synthons that are currently commercially available.^62^

In this paper, we have applied the platform mainly to BRD4-degrading PROTACs and, by using a model toolkit, we have demonstrated the compatibility of the substructures accessible by the MCRs and placed at the attachment point between the warhead and the linker with degradation activity, aqueous solubility, and in vitro metabolic stability, despite the presence of additional HBAs and HBDs and potential soft spots. The full characterization and development of BRD4-degrading PROTACs are beyond the scope of the present report; however, we have identified a cluster of CRBN-binding PROTACs (**27**, **28**, **29**, **34**, and **37**) that cause a significant and proteasomal-dependent reduction of BRD4 levels. At this stage, compound **34**, displaying the privileged piperazine ring, and **37**, endowed with an α-acyloxy amide, stand out for higher degrading activity, with DC_50_ values of 60 and 62 nM, respectively, in MDA-MB-231 cells, and properties compatible with further preclinical development.

For the time being, we have only scraped the surface of the potential of the platform in the protein degraders field: the described variants have many applications and can be easily applied to other proteins for which ligands are available. Making protein degraders easily accessible to chemists at a reasonable cost must be a major objective in the time to come, and we envision that our method will help to achieve this goal.

## Experimental Section

### Chemistry

Commercially available reagents and solvents were used as purchased without further purification. When needed, solvents were distilled and stored on molecular sieves. Reactions were monitored by thin layer chromatography (TLC) carried out on 5 cm × 20 cm silica gel plates with a layer thickness of 0.25 mm, using UV light as a visualizing agent. When necessary, TLC plates were visualized with aqueous KMnO_4_ or aqueous acidic solution of cerium sulfate and ammonium molybdate reagent. Column chromatography was performed on flash silica gel using Kieselgel 60 silica gel (particle size 0.040–0.063 mm, 230–400 mesh). Melting points were determined in open glass capillary with a Stuart Scientific SMP3 apparatus. All the target compounds were checked by IR (FT-IR Bruker Alpha II), ^1^H NMR and ^13^C NMR (Bruker Avance Neo 400 MHz), and HRMS (Thermo Fisher Q-Exactive Plus) equipped with an Orbitrap (ion trap) mass analyzer. Chemical shifts are reported in parts per million (ppm) with residual solvent signals as internal standard (CDCl_3_, δ = 7.26 ppm for ^1^H NMR, δ = 77.16 ppm for ^13^C NMR; CD_3_OD, δ = 3.31 ppm for ^1^H NMR, δ = 49.00 ppm for ^13^C NMR; (CD_3_)_2_CO, δ = 2.05 ppm for ^1^H NMR, δ = 29.84, 206.26 ppm for ^13^C NMR; (CD_3_)_2_SO, δ = 2.50 ppm for ^1^H NMR, δ = 39.52 ppm for ^13^C NMR). Coupling constants (*J*) are quoted in hertz (Hz). Abbreviations used for multiplicity are as follows: s, singlet; d, doublet; t, triplet; q, quartet; quint, quintet; dd, doublet of doublets; dt, doublet of triplets; br, broad; m, multiplet. All PROTACs based on JQ1 structure were obtained as a mixture of diastereomers which include the racemic mixture at the IMiD stereocenter with enantiopure (+)-configuration at the JQ1 stereocenter.

The purity of selected compounds was determined by HPLC using a Shimadzu HPLC system (Shimadzu, Kyoto, Japan) equipped with a Kinetex C18 (150 × 4.6 mm, 5 μm d.p., Phenomenex Torrance, CA) and using 0.2% formic acid in water and 0.2% formic acid in acetonitrile as eluents (for further details, see Supporting Information, SI). The purity of all tested compounds is 95% or higher.

### General Procedure A for the Synthesis of Isocyanides **16**–**20**

Amino alcohol (7.50 mmol, 1 equiv) **1**–**5** and ethyl formate (5.00 mL) were heated under reflux for 6 h. Then the volatile was removed in vacuo. The corresponding formamide (7.50 mmol, 1 equiv) was solubilized in dry CH_2_Cl_2_ (14.0 mL) under nitrogen and, after adding TEA (45.0 mmol, 6 equiv), the reaction mixture was cooled to 0 °C. Then TsCl (22.5 mmol, 3 equiv) was added, and the reaction was stirred at room temperature for 5 h. The mixture was quenched with a saturated aqueous Na_2_CO_3_ solution and stirred at 0 °C for 30 min. Water was added, and the aqueous phase was extracted with CH_2_Cl_2_ (×3). The combined organic layers were dried over sodium sulfate and evaporated. The crude product was purified by column chromatography using the eluent indicated below.

#### 3-Isocyanopropyl 4-Methylbenzenesulfonate (**16**)

The title compound was synthesized following the general procedure A. The crude material was purified by column chromatography using PE/EtOAc 95:5 as eluent, affording compound **16** (1.31 g, 73%) as a yellow oil. ^1^H NMR (400 MHz; CDCl_3_): δ 7.82 (d, *J* = 8.4 Hz, 2H), 7.39 (d, *J* = 8.4 Hz, 2H), 4.19 (t, *J* = 5.8 Hz, 2H), 3.51 (t, *J* = 5.8 Hz, 2H), 2.48 (s, 3H), 2.05 (quint, *J* = 5.8 Hz, 2H). ^13^C NMR (101 MHz; CDCl_3_): δ 157.5, 145.3, 132.4, 130.1, 127.9, 65.9, 37.8, 28.7, 21.7. HRMS (ESI) *m*/*z* (M + Na)^+^ calcd for C_11_H_13_NO_3_SNa 262.0503, found 262.0504. IR (neat): ν̃ = 2969, 2149, 1598, 1356, 1174, 1020, 944, 814, 664, 554 cm^–1^.

#### 4-Isocyanobutyl 4-Methylbenzenesulfonate (**17**)

The title compound was synthesized following the general procedure A. The crude material was purified by column chromatography using PE/EtOAc 95:5 as eluent, affording compound **17** (1.23 g, 65%) as a yellow oil. ^1^H NMR (400 MHz; CDCl_3_): δ 7.80 (d, *J* = 8.4 Hz, 2H), 7.38 (d, *J* = 8.4 Hz, 2H), 4.09 (t, *J* = 5.9 Hz, 2H), 3.41 (t, *J* = 5.9 Hz, 2H), 2.48 (s, 3H), 1.87–1.82 (m, 2H), 1.78–1.74 (m, 2H). ^13^C NMR (101 MHz; CDCl_3_): δ 156.8, 145.0, 132.9, 130.0, 127.9, 69.5, 40.9, 25.8, 25.2, 21.6. HRMS (ESI) *m*/*z* (M + Na)^+^ calcd for C_12_H_15_NO_3_SNa 276.0659, found 276.0663. IR (neat): ν̃ = 2923, 2148, 1597, 1354, 1174, 1018, 929, 814, 663, 555 cm^–1^.

#### 5-Isocyanopentyl 4-Methylbenzenesulfonate (**18**)

The title compound was synthesized following the general procedure A. The crude material was purified by column chromatography using PE/EtOAc 9:1 as eluent, affording compound **18** (1.14 g, 57%) as a yellow oil. ^1^H NMR (400 MHz; CDCl_3_): δ 7.81 (d, *J* = 8.4 Hz, 2H), 7.38 (d, *J* = 8.4 Hz, 2H), 4.06 (t, *J* = 6.3 Hz, 2H), 3.37 (dt, *J*_*s*_ = 6.6, 1.9 Hz, 2H), 2.48 (s, 3H), 1.75–1.63 (m, 4H), 1.53–1.47 (m, 2H). ^13^C NMR (101 MHz; CDCl_3_): δ 155.9, 145.0, 132.8, 130.0, 127.8, 70.1, 41.3, 28.3, 27.9, 22.3, 21.6. HRMS (ESI) *m*/*z* (M + H)^+^ calcd for C_13_H_18_NO_3_S 268.1002, found 268.1001. IR (neat): ν̃ = 2950, 2148, 1597, 1353, 1174, 1019, 948, 815, 664, 555 cm^–1^.

#### 2-(2-Isocyanoethoxy)ethyl 4-Methylbenzenesulfonate (**19**)

The title compound was synthesized following the general procedure A. The crude material was purified by column chromatography using PE/EtOAc 9:1 as eluent, affording compound **19** (1.31 g, 65%) as a yellow oil. ^1^H NMR (400 MHz; CDCl_3_): δ 7.82 (d, *J* = 8.4 Hz, 2H), 7.38 (d, *J* = 8.4 Hz, 2H), 4.19 (t, *J* = 4.6 Hz, 2H), 3.78 (t, *J* = 4.7 Hz, 2H), 3.64 (t, *J* = 4.6 Hz, 2H), 3.51 (t, *J* = 4.7 Hz, 2H), 2.47 (s, 3H). ^13^C NMR (101 MHz; CDCl_3_): δ 157.7, 145.0, 132.9, 129.9, 127.9, 69.0, 68.9, 68.6, 41.6, 21.6. HRMS (ESI) *m*/*z* (M + Na)^+^ calcd for C_12_H_15_NO_4_SNa 292.0609, found 292.0609. IR (neat): ν̃ = 2958, 2152, 1597, 1352, 1174, 1010, 919, 815, 663, 554 cm^–1^.

#### 2-(2-(2-Isocyanoethoxy)ethoxy)ethyl 4-Methylbenzenesulfonate (**20**)

The title compound was synthesized following the general procedure A. The crude material was purified by column chromatography using PE/EtOAc 6:4 as eluent, affording compound **20** (1.67 g, 71%) as a yellow oil. ^1^H NMR (400 MHz; CDCl_3_): δ 7.82 (d, *J* = 8.2 Hz, 2H), 7.37 (d, *J* = 8.2 Hz, 2H), 4.19 (t, *J* = 4.7 Hz, 2H), 3.72 (t, *J* = 4.8 Hz, 2H), 3.70–3.67 (m, 2H), 3.65–3.62 (m, 4H), 3.58–3.54 (m, 2H), 2.47 (s, 3H). ^13^C NMR (101 MHz; CDCl_3_): δ 157.4, 144.9, 133.1, 129.9, 127.9, 70.8 (2C), 69.2, 68.8, 68.7, 41.8, 21.6. HRMS (ESI) *m*/*z* (M + Na)^+^ calcd for C_14_H_19_NO_5_SNa 336.0871, found 336.0872. IR (neat): ν̃ = 2874, 2152, 1701, 1612, 1350, 1257, 1194, 1052, 734, 574 cm^–1^.

### General Procedure B for the Synthesis of Thalidomide-Bearing Isocyanides **1**–**5**

To a solution of thalidomide derivative **21** (2.20 mmol, 1 equiv) in dry DMF (6.00 mL) was added NaHCO_3_ (3.30 mmol, 1.5 equiv) under nitrogen, and the reaction mixture was heated at 65 °C. After 15 min, a solution of isocyanide **16**–**20** (2.64 mmol, 1.2 equiv) in dry DMF (500 μL) was added dropwise and the resulting mixture was stirred at 65 °C overnight. The reaction mixture was diluted with CH_2_Cl_2_ and washed with water (×3). The organic layer was dried over sodium sulfate and the volatile solvent was removed in vacuo. The crude product was purified by column chromatography using the eluent indicated below.

#### 2-(2,6-Dioxopiperidin-3-yl)-4-(3-isocyanopropoxy)isoindoline-1,3-dione (**1**)

The title compound was synthesized following the general procedure B, starting from isocyanide **16**. The crude material was purified by column chromatography using PE/EtOAc 6:4 as eluent, affording compound **1** (616 mg, 82%) as a yellow solid. ^1^H NMR (400 MHz; CDCl_3_): δ 8.38 (br s, 1H), 7.73 (t, *J* = 7.4 Hz, 1H), 7.51 (d, *J* = 7.4, 1H), 7.27 (d, *J* = 7.4 Hz, 1H), 4.97 (dd, *J*_*s*_ = 12.3, 5.5 Hz, 1H), 4.34 (t, *J* = 5.6 Hz, 2H), 3.77 (t, *J* = 5.6 Hz, 2H), 2.93–2.88 (m, 1H), 2.85–2.75 (m, 2H), 2.25 (quint, *J* = 5.6 Hz, 2H), 2.17–2.14 (m, 1H). ^13^C NMR (101 MHz; CDCl_3_): δ 171.0, 168.1, 166.9, 166.1, 157.1, 155.9, 136.7, 133.8, 119.1, 117.6, 116.5, 64.9, 49.2, 38.2, 31.4, 28.8, 22.6. HRMS (ESI) *m*/*z* (M + Na)^+^ calcd for C_17_H_15_N_3_O_5_Na 364.0898, found 364.0899. IR (neat): ν̃ = 3094, 2153, 1698, 1614, 1396, 1200, 1051, 744, 610, 471 cm^–1^. Mp: 199.7–201.1 °C, dec.

#### 2-(2,6-Dioxopiperidin-3-yl)-4-(4-isocyanobutoxy)isoindoline-1,3-dione (**2**)

The title compound was synthesized following the general procedure B, starting from isocyanide **17**. The crude material was purified by column chromatography using PE/EtOAc 6:4 as eluent, affording compound **2** (539 mg, 69%) as a white solid. ^1^H NMR (400 MHz; DMSO-*d*_6_): δ 11.10 (br s, 1H), 7.82 (t, *J* = 8.4 Hz, 1H), 7.52 (d, *J* = 8.4 Hz, 1H), 7.46 (d, *J* = 7.2 Hz, 1H), 5.09 (dd, *J*_*s*_ = 12.8, 5.4 Hz, 1H), 4.27 (t, *J* = 5.7 Hz, 2H), 3.65 (t, *J* = 5.7 Hz, 2H), 2.93–2.84 (m, 1H), 2.63–2.61 (m, 1H), 2.59–2.57 (m, 1H), 2.06–2.00 (m, 1H), 1.89–1.82 (m, 4H). ^13^C NMR (101 MHz; DMSO-*d*_6_): δ 173.2, 170.4, 167.3, 165.9, 156.3, 156.0, 137.5, 133.7, 120.3, 116.8, 115.8, 68.6, 49.3, 41.5, 31.4, 25.9, 25.8, 22.5. HRMS (ESI) *m*/*z* (M + Na)^+^ calcd for C_18_H_17_N_3_O_5_Na 378.1055, found 378.1058. IR (neat): ν̃ = 3085, 2149, 1704, 1612, 1349, 1202, 1047, 747, 609, 464 cm^–1^. Mp: 201.7–202.0 °C, dec.

#### 2-(2,6-Dioxopiperidin-3-yl)-4-((5-isocyanopentyl)oxy)isoindoline-1,3-dione (**3**)

The title compound was synthesized following the general procedure B, starting from isocyanide **18**. The crude material was purified by column chromatography using PE/EtOAc 5:5 as eluent, affording compound **3** (674 mg, 83%) as a white solid. ^1^H NMR (400 MHz; CDCl_3_): δ 8.25 (br s, 1H), 7.70 (t, *J* = 8.4 Hz, 1H), 7.48 (d, *J* = 8.4 Hz, 1H), 7.23 (d, *J* = 8.4 Hz, 1H), 4.97 (dd, *J*_*s*_ = 12.3, 5.3 Hz, 1H), 4.22 (t, *J* = 6.2 Hz, 2H), 3.47 (tt, *J*_*s*_ = 6.2, 1.9 Hz, 2H), 2.93–2.89 (m, 1H), 2.86–2.82 (m, 1H), 2.79–2.74 (m, 1H), 2.18–2.12 (m, 1H), 1.95 (quint, *J* = 6.2 Hz, 2H), 1.85–1.82 (m, 2H), 1.76–1.70 (m, 2H). ^13^C NMR (101 MHz; CDCl_3_): δ 171.1, 168.2, 167.0, 165.6, 156.4, 156.2, 136.5, 133.8, 119.0, 117.3, 116.0, 69.1, 49.1, 41.4, 31.4, 28.7, 28.0, 23.0, 22.6. HRMS (ESI) *m*/*z* (M + Na)^+^ calcd for C_19_H_19_N_3_O_5_Na 392.1211, found 392.1208. IR (neat): ν̃ = 3100, 2145, 1697, 1613, 1365, 1195, 1049, 745, 613, 469 cm^–1^. Mp: 174.6–175.9 °C, dec.

#### 2-(2,6-Dioxopiperidin-3-yl)-4-(2-(2-isocyanoethoxy)ethoxy)isoindoline-1,3-dione (**4**)

The title compound was synthesized following the general procedure B, starting from isocyanide **19**. The crude material was purified by column chromatography using PE/EtOAc 5:5 as eluent, affording compound **4** (588 mg, 72%) as a yellow solid. ^1^H NMR (400 MHz; CDCl_3_): δ 8.33 (br s, 1H), 7.71 (t, *J* = 7.2 Hz, 1H), 7.50 (d, *J* = 7.2 Hz, 1H), 7.29 (d, *J* = 7.2 Hz, 1H), 4.98 (dd, *J*_*s*_ = 12.1, 5.3 Hz, 1H), 4.38 (t, *J* = 4.5 Hz, 2H), 4.01 (t, *J* = 4.5 Hz, 2H), 3.91–3.88 (m, 2H), 3.62 (t, *J* = 5.5 Hz, 2H), 2.93–2.85 (m, 2H), 2.81–2.75 (m, 1H), 2.18–2.13 (m, 1H). ^13^C NMR (101 MHz; DMSO-*d*_6_): δ 173.2, 170.4, 167.3, 165.8, 156.8, 156.2, 137.5, 133.7, 120.6, 116.9, 116.0, 69.4, 69.1, 68.8, 49.3, 42.1, 31.4, 22.5. HRMS (ESI) *m*/*z* (M + H)^+^ calcd for C_18_H_18_N_3_O_6_ 372.1190, found 372.1193. IR (neat): ν̃ = 3082, 2156, 1680, 1613, 1365, 1198, 1054, 744, 613, 467 cm^–1^. Mp: 183.7–185.2 °C, dec.

#### 2-(2,6-Dioxopiperidin-3-yl)-4-(2-(2-(2-isocyanoethoxy)ethoxy)ethoxy)isoindoline-1,3-dione (**5**)

The title compound was synthesized following the general procedure B, starting from isocyanide **20**. The crude material was purified by column chromatography using PE/EtOAc 4:6 as eluent, affording compound **5** (420 mg, 46%) as a white solid. ^1^H NMR (400 MHz; CDCl_3_): δ 8.29 (br s, 1H), 7.70 (t, *J* = 7.8 Hz, 1H), 7.49 (d, *J* = 7.8 Hz, 1H), 7.28 (d, *J* = 7.8 Hz, 1H), 4.98 (dd, *J*_*s*_ = 12.2, 5.2 Hz, 1H), 4.37 (t, *J* = 4.6 Hz, 2H), 3.98 (t, *J* = 4.6 Hz, 2H), 3.84–3.81 (m, 2H), 3.74–3.71 (m, 4H), 3.58 (t, *J* = 5.8 Hz, 2H), 2.92–2.82 (m, 2H), 2.79–74 (m, 1H), 2.16–2.13 (m, 1H). ^13^C NMR (101 MHz; DMSO-*d*_6_): δ 173.2, 170.4, 167.3, 165.8, 156.3, 153.4, 137.5, 133.7, 120.5, 116.8, 115.9, 70.6, 70.2, 69.4, 69.2, 68.5, 49.3, 42.0, 31.1, 22.5. HRMS (ESI) *m*/*z* (M + Na)^+^ calcd for C_20_H_21_N_3_O_7_Na 438.1266, found 438.1270. IR (neat): ν̃ = 3110, 2152, 1701, 1613, 1350, 1194, 1052, 747, 608, 467 cm^–1^. Mp: 188.9–190.3 °C, dec.

### General Procedure C for Methylamine-Mediated Ugi Reactions **22**–**26**

To a solution of methylamine **8** (40% aqueous solution, 0.240 mmol, 2 equiv) in MeOH (500 μL) were added formaldehyde **7** (37% aqueous solution, 0.360 mmol, 3 equiv), isocyanide **1**–**5** (0.180 mmol, 1.5 equiv), and carboxylic acid **6** (0.120 mmol, 1 equiv) at 0 °C and stirred for 2 h. The reaction mixture was left to reach room temperature and stirred for another 1 h. The volatile solvent was removed in vacuo, and the crude product was purified by column chromatography using the eluent indicated below.

#### 2-((*S*)-4-(4-Chlorophenyl)-2,3,9-trimethyl-6*H*-thieno[3,2-*f*][1,2,4]triazolo[4,3-*a*][1,4]diazepin-6-yl)-*N*-(2-((3-((2-(2,6-dioxopiperidin-3-yl)-1,3-dioxoisoindolin-4-yl)oxy)propyl)amino)-2-oxoethyl)-*N*-methylacetamide (**22**)

The title compound was synthesized following the general procedure C, starting from thalidomide-bearing isocyanide **1**. The crude material was purified by column chromatography using CH_3_CN/MeOH 95:5 as eluent, affording compound **22** (70.6 mg, 75%) as a white solid. ^1^H NMR (400 MHz; DMSO-*d*_6_, 353 K, *: refers to the main rotamer): δ 10.78 (br s, 1H), 8.08 (br s, 1H), 7.75 (t, *J* = 8.1 Hz, 1H), 7.47–7.44 (m, 5H), 7.42 (d, *J* = 7.1 Hz, 1H), 5.05 (dd, *J*_*s*_ = 12.5, 5.4 Hz, 1H), 4.59 (t, *J* = 6.7 Hz, 1H), 4.26 (t, *J* = 6.2 Hz, 2H), 4.22–4.19 (m, 1H), 3.98–3.95 (m, 1H), 3.84–3.80 (m, 1H)*, 3.42–3.37 (m, 1H), 3.33 (t, *J* = 6.3 Hz, 2H), 3.21 (s, 3H), 2.92–2.83 (m, 2H), 2.65–2–63 (m, 1H)*, 2.60 (s, 3H), 2.42 (s, 3H), 2.09–2.03 (m, 1H), 1.96 (quint, *J* = 6.3 Hz, 2H), 1.66 (s, 3H). ^13^C NMR (101 MHz; CD_3_OD, *: refers to the main rotamer): δ = 173.1*, 171.8, 169.9, 169.3, 167.2*, 164.8*, 156.1, 155.8, 150.7, 136.8, 136.6 (2C), 133.5, 133.4, 132.1, 131.8*, 130.7, 130.6, 130.0*, 128.4, 119.1*, 116.8, 115.2, 67.7*, 54.0*, 51.0, 49.1*, 36.6*, 36.1, 34.9, 30.8, 28.4*, 22.3, 13.0, 11.6, 10.3. HRMS (ESI) *m*/*z* (M + H)^+^ calcd for C_38_H_38_ClN_8_O_7_S 785.2267, found 785.2255. IR (neat): ν̃ = 3368, 2924, 1708, 1643, 1355, 1196, 1049, 747, 545, 466 cm^–1^. Mp: 211.5–213.8 °C, dec. See SI Figure S1 for NMR spectra of compound **22**.

#### 2-((*S*)-4-(4-Chlorophenyl)-2,3,9-trimethyl-6*H*-thieno[3,2-*f*][1,2,4]triazolo[4,3-*a*][1,4]diazepin-6-yl)-*N*-(2-((4-((2-(2,6-dioxopiperidin-3-yl)-1,3-dioxoisoindolin-4-yl)oxy)butyl)amino)-2-oxoethyl)-*N*-methylacetamide (**23**)

The title compound was synthesized following the general procedure C, starting from thalidomide-bearing isocyanide **2**. The crude material was purified by column chromatography using CH_3_CN/MeOH 98:2 as eluent, affording compound **23** (69.9 mg, 73%) as a white solid. ^1^H NMR (400 MHz; DMSO-*d*_6_, 353 K): δ 7.78 (t, *J* = 7.9 Hz, 1H), 7.48–7.42 (m, 6H), 5.04 (dd, *J*_*s*_ = 12.6, 5.3 Hz, 1H), 4.59 (t, *J* = 6.7 Hz, 1H), 4.28 (t, *J* = 6.7 Hz, 2H), 4.21–4.19 (m, 1H), 3.97–3.95 (m, 1H), 3.23–3.18 (m, 5H), 3.05–3.03 (s, 2H), 2.92–2.84 (m, 2H), 2.65–2.63 (m, 1H), 2.42 (s, 3H), 2.60 (s, 3H), 2.09–2.03 (m, 1H), 1.82–1.78 (m, 4H), 1.66 (s, 3H). ^13^C NMR (101 MHz; CD_3_OD, *: refers to the main rotamer): δ 173.1*, 171.8*, 169.6, 169.2, 167.2*, 166.1*, 164.8*, 156.4*,155.9*, 150.8*, 136.8, 136.6, 136.5, 133.6*, 132.1, 131.8*, 130.7, 130.5, 130.0, 128.4, 119.2*, 116.7*, 115.0, 69.0, 54.0*, 51.0, 49.0, 38.5*, 36.1, 34.9, 34.2, 30.8, 25.7*, 22.3, 13.0, 11.6, 10.3. HRMS (ESI) *m*/*z* (M + H)^+^ calcd for C_39_H_40_ClN_8_O_7_S 799.2424, found 799.2408. IR (neat): ν̃ = 3339, 2922, 1706, 1648, 1393, 1194, 1048, 747, 558, 466 cm^–1^. Mp: 190.5–192.0 °C, dec. See SI Figure S2 for the NMR spectra of compound **23**.

#### 2-((*S*)-4-(4-Chlorophenyl)-2,3,9-trimethyl-6*H*-thieno[3,2-*f*][1,2,4]triazolo[4,3-*a*][1,4]diazepin-6-yl)-*N*-(2-((5-((2-(2,6-dioxopiperidin-3-yl)-1,3-dioxoisoindolin-4-yl)oxy)pentyl)amino)-2-oxoethyl)-*N*-methylacetamide (**24**)

The title compound was synthesized following the general procedure C, starting from thalidomide-bearing isocyanide **3**. The crude material was purified by column chromatography using CH_3_CN/MeOH 98:2 as eluent, affording compound **24** (63.4 mg, 65%) as a yellow solid. ^1^H NMR (400 MHz; DMSO-*d*_6_, 353 K): δ 7.77 (t, *J* = 7.9 Hz, 1H), 7.48–7.44 (m, 5H), 7.43–7.41 (m, 1H), 5.03 (dd, *J*_*s*_ = 12.3, 5.4 Hz, 1H), 4.62 (t, *J* = 6.7 Hz, 1H), 4.24 (t, *J* = 6.4 Hz, 2H), 4–07–4.03 (m, 1H), 3.68–3.55 (m, 2H), 3.45–3.41 (m, 1H), 3.23 (s, 3H), 3.18 (q, *J* = 6.4 Hz, 2H), 2.69–2.65 (m, 2H), 2.61 (s, 3H), 2.58–2.57 (m, 1H), 2.43 (s, 3H), 2.12–2.06 (m, 1H), 1.81 (quint, *J* = 6.4 Hz, 2H), 1.68 (s, 3H), 1.59–1.49 (m, 4H). ^13^C NMR (101 MHz; CD_3_OD, *: refers to the main rotamer): δ 173.2, 171.8*, 170.0, 169.6, 169.1, 167.3, 166.0, 165.0*, 156.6*, 155.9*, 150.8, 136.8, 136.5*, 133.7, 132.1, 131.8, 130.7, 130.6, 129.4*, 128.4, 119.1, 116.8, 114.9, 69.0, 54.0*, 51.0, 49.0, 38.8*, 36.0, 34.9*, 30.8, 28.6*, 28.2*, 22.8, 22.3, 13.0, 11.5, 10.2. HRMS (ESI) *m*/*z* (M + Na)^+^ calcd for C_40_H_41_ClN_8_O_7_SNa 835.2394, found 835.2381. IR (neat): ν̃ = 3294, 2924, 1709, 1644, 1355, 1196, 1048, 747, 545, 466 cm^–1^. Mp: 175.2–176.8 °C, dec. See SI Figure S3 for NMR spectra of compound **24**.

#### 2-((*S*)-4-(4-Chlorophenyl)-2,3,9-trimethyl-6*H*-thieno[3,2-*f*][1,2,4]triazolo[4,3-*a*][1,4]diazepin-6-yl)-*N*-(2-((2-(2-((2-(2,6-dioxopiperidin-3-yl)-1,3-dioxoisoindolin-4-yl)oxy)ethoxy)ethyl)amino)-2-oxoethyl)-*N*-methylacetamide (**25**)

The title compound was synthesized following the general procedure C, starting from thalidomide-bearing isocyanide **4**. The crude material was purified by column chromatography using CH_3_CN/MeOH 98:2 as eluent, affording compound **25** (52.8 mg, 54%) as a yellow solid. ^1^H NMR (400 MHz; DMSO-*d*_6_, 353 K): δ 10.81 (br s, 1H), 8.01 (br s, 1H), 7.78 (t, *J* = 7.8 Hz, 1H), 7.54–7.51 (m, 1H), 7.49–7.43 (m, 5H), 5.05 (dd, *J*_*s*_ = 12.4, 5.3 Hz, 1H), 4.58 (t, *J* = 6.7 Hz, 1H), 4.36 (t, *J* = 4.7 Hz, 2H), 4.22–4.17 (m, 1H), 3.98–3.94 (m, 1H), 3.83–3.80 (m, 2H), 3.64–3.62 (m, 1H), 3.60–3.55 (m, 2H), 3.46–3.44 (m, 1H), 3.32–3.23 (m, 2H), 3.21 (s, 3H), 2.91–2.83 (m, 2H), 2.68–2.63 (m, 1H), 2.60 (s, 3H), 2.42 (s, 3H), 2.08–2.04 (m, 1H), 1.66 (s, 3H). ^13^C NMR (101 MHz; CD_3_OD, *: refers to the main rotamer): δ = 173.1, 171.8*, 170.0, 169.8, 167.1, 166.1, 164.8*, 156.4*, 155.8*, 150.8, 136.8, 136.6, 136.5, 133.6, 132.1, 131.8, 130.7, 130.6, 130.0*, 128.4, 119.5*, 116.9, 115.3, 69.3, 68.9, 68.6, 54.0*, 50.7, 49.1, 39.0*, 35.9, 34.9*, 30.8, 22.3, 13.0, 11.6, 10.3. HRMS (ESI) *m*/*z* (M + Na)^+^calcd for C_39_H_39_ClN_8_O_8_SNa 837.2187, found 837.2184. IR (neat): ν̃ = 3274, 2923, 1707, 1644, 1354, 1196, 1051, 747, 545, 466 cm^–1^. Mp: 155.6–156.8 °C, dec. See SI Figure S4 for NMR spectra of compound **25**.

#### 2-((*S*)-4-(4-Chlorophenyl)-2,3,9-trimethyl-6*H*-thieno[3,2-*f*][1,2,4]triazolo[4,3-*a*][1,4]diazepin-6-yl)-*N*-(2-((2-(2-(2-((2-(2,6-dioxopiperidin-3-yl)-1,3-dioxoisoindolin-4-yl)oxy)ethoxy)ethoxy)ethyl)amino)-2-oxoethyl)-*N*-methylacetamide (**26**)

The title compound was synthesized following the general procedure C, starting from thalidomide-bearing isocyanide **5**. The crude material was purified by column chromatography using CH_3_CN/MeOH 97:3 as eluent, affording compound **26** (73.1 mg, 71%) as a yellow solid. ^1^H NMR (400 MHz; DMSO-*d*_6_, 353 K): δ 10.81 (br s, 1H), 7.98 (br s, 1H), 7.79 (t, *J* = 7.8 Hz, 1H), 7.52–7.49 (m, 2H), 7.47–7.44 (m, 4H), 5.05 (dd, *J*_*s*_ = 12.5, 5.4 Hz, 1H), 4.59 (t, *J* = 6.7 Hz, 1H), 4.36 (t, *J* = 4.7 Hz, 2H), 4.22–4.18 (m, 1H), 3.98–3.94 (m, 1H), 3.82 (d, *J* = 4.7 Hz, 2H), 3.65 (t, *J* = 4.4 Hz, 2H), 3.57–3.53 (m, 4H), 3.47 (t, *J* = 5.9 Hz, 2H), 3.29–3.26 (m, 2H), 3.21 (s, 3H), 2.91–2.83 (m, 2H), 2.66–2.63 (m, 1H), 2.60 (s, 3H), 2.42 (s, 3H), 2.10–2.04 (m, 1H), 1.66 (m, 3H). ^13^C NMR (101 MHz; CD_3_OD, *: refers to the main rotamer): δ 173.1*, 171.8*, 170.0, 169.7, 167.2, 165.9, 164.8*, 156.4, 155.8, 150.7, 136.8, 136.5, 133.7, 132.1*, 131.8, 130.7, 130.6 (2C), 130.0, 128.4, 119.6, 116.9, 115.3, 70.6*, 70.0*, 69.1 (2C), 69.0, 54.0*, 50.8*, 49.1, 39.0*, 35.9, 34.9*, 30.8, 22.3, 13.0, 11.6, 10.2. HRMS (ESI) *m*/*z* (M + Na)^+^ calcd for C_41_H_43_ClN_8_O_9_SNa 881.2449, found 881.2442. IR (neat): ν̃ = 3294, 2923, 1707, 1645, 1353, 1196, 1051, 747, 546, 465 cm^–1^. Mp: 152.5–153.5 °C, dec. See SI Figure S5 for NMR spectra of compound **26**.

### General Procedure D for Tritylamine-Mediated Ugi Reactions **27**–**31**

A solution of tritylamine **9** (0.120 mmol, 1 equiv) and paraformaldehyde **7** (0.240 mmol, 2 equiv) in MeOH (500 μL) was stirred at 40 °C for 1 h. The reaction was then cooled to room temperature, and isocyanide **1**–**5** (0.120 mmol, 1 equiv) and carboxylic acid **6** (0.120 mmol, 1 equiv) were added. The resulting mixture was stirred overnight. The following day, the volatile solvent was removed in vacuo and the crude product was solubilized in CH_2_Cl_2_ (256 μL). At 0 °C, TFA (256 μL) was added and, after 30 min, the mixture was allowed to reach room temperature and stirred for 3 h. The crude material was subjected to column chromatography using Cy/EtOAc 5:5 to remove TFA and then CH_3_CN/MeOH in the proportion indicated for each compound to elute the desired product.

#### 2-((*S*)-4-(4-Chlorophenyl)-2,3,9-trimethyl-6*H*-thieno[3,2-*f*][1,2,4]triazolo[4,3-*a*][1,4]diazepin-6-yl)-*N*-(2-((3-((2-(2,6-dioxopiperidin-3-yl)-1,3-dioxoisoindolin-4-yl)oxy)propyl)amino)-2-oxoethyl)acetamide (**27**)

The title compound was synthesized following the general procedure D, starting from thalidomide-bearing isocyanide **1**. The crude material was purified by column chromatography using Cy/EtOAc 5:5 and CH_3_CN/MeOH 9:1 as eluents, affording compound **27** (75.8 mg, 82%) as a white solid. ^1^H NMR (400 MHz; (CD_3_)_2_CO): δ 10.02 (br s, 1H), 8.03 (br t, *J* = 5.8 Hz, 1H), 7.78–7.76 (m, 1H), 7.73 (t, *J* = 7.9 Hz, 1H), 7.53 (d, *J* = 8.2 Hz, 2H), 7.44–7.40 (m, 3H), 7.38 (d, *J* = 7.9 Hz, 1H), 5.12 (dd, *J*_*s*_ = 13.5, 4.4 Hz, 1H), 4.66 (t, *J* = 6.2 Hz, 1H), 4.28 (t, *J* = 6.5 Hz, 2H), 4.11–4.04 (m, 1H), 3.78 (dt, *J*_*s*_ = 16.8, 4.5 Hz, 1H), 3.58–3.52 (m, 1H), 3.47 (t, *J* = 5.3 Hz, 2H), 3.36 (dd, *J*_*s*_ = 14.9, 6.5 Hz, 1H), 2.91–2.88 (m, 1H), 2.78–2.71 (m, 2H), 2.62 (s, 3H), 2.43 (s, 3H), 2.25–2.19 (m, 1H), 2.03–2.01 (m, 2H), 1.70 (s, 3H). ^13^C NMR (101 MHz; (CD_3_)_2_CO, *: refers to the main rotamer): δ 171.8*, 170.4*, 169.3, 169.2*, 166.9, 166.0, 163.8, 156.4, 155.8, 150.0, 137.3, 136.7, 135.9, 133.8, 132.6, 130.9*, 130.5, 130.3, 130.2, 128.4, 119.4, 117.0, 115.1, 67.9, 54.4, 49.3, 42.8*, 38.5, 36.4*, 31.1, 29.7, 22.5, 13.6, 12.1, 10.9. HRMS (ESI) *m*/*z* (M + Na)^+^ calcd for C_37_H_35_ClN_8_O_7_SNa 793.1925, found 793.1920. IR (neat): ν̃ = 3351, 2922, 1707, 1656, 1394, 1196, 1049, 748, 529, 467 cm^–1^. Mp: 221.3–223.6 °C, dec HPLC purity: > 99%. See SI Figure S6 for NMR spectra of compound **27** and Figure S20 for the HPLC chromatogram.

#### 2-((*S*)-4-(4-Chlorophenyl)-2,3,9-trimethyl-6*H*-thieno[3,2-*f*][1,2,4]triazolo[4,3-*a*][1,4]diazepin-6-yl)-*N*-(2-((4-((2-(2,6-dioxopiperidin-3-yl)-1,3-dioxoisoindolin-4-yl)oxy)butyl)amino)-2-oxoethyl)acetamide (**28**)

The title compound was synthesized following the general procedure D, starting from thalidomide-bearing isocyanide **2**. The crude material was purified by column chromatography using Cy/EtOAc 5:5 and CH_3_CN/MeOH 9:1 as eluents, affording compound **28** (51.8 mg, 55%) as a white solid. ^1^H NMR (400 MHz; (CD_3_)_2_CO): δ 10.03 (br d, *J* = 9.3 Hz, 1H), 8.17 (br t, *J* = 6.3 Hz, 1H), 7.76–7.72 (m, 2H), 7.53 (d, *J* = 8.2 Hz, 2H), 7.44–7.41 (m, 3H), 7.37 (d, *J* = 7.2 Hz, 1H), 5.11 (dd, *J*_*s*_ = 12.5, 5.4 Hz, 1H), 4.67 (t, *J* = 8.5 Hz, 1H), 4.20 (t, *J* = 6.4 Hz, 2H), 4.16–4.10 (m, 1H), 3.70–3.66 (m, 1H), 3.60–3.59 (m, 1H), 3.38–3.36 (m, 1H), 3.34–3.30 (m, 2H), 2.92–2.89 (m, 1H), 2.79–2.73 (m, 2H), 2.62 (s, 3H), 2.42 (s, 3H), 2.23–2.18 (m, 1H), 1.84–1.80 (m, 2H), 1.75–1.72 (m, 2H), 1.69 (s, 3H). ^13^C NMR (101 MHz; (CD_3_)_2_CO): δ 171.9, 170.4, 169.3, 169.2, 167.0, 165.6, 163.9 (2C), 156.6, 137.3 (2C), 136.7, 135.9, 133.9, 132.6, 131.0, 130.6, 130.3, 130.2, 128.4, 119.5, 117.0, 114.9, 69.1, 54.5, 49.2, 42.7, 38.5, 38.4, 31.1, 26.0, 25.9, 22.4, 13.6, 12.1, 10.9. HRMS (ESI) *m*/*z* (M + H)^+^ calcd for C_38_H_38_ClN_8_O_7_S 785.2267, found 785.2260. IR (neat): ν̃ = 3323, 2929, 1709, 1651, 1394, 1195, 1048, 748, 528, 465 cm^–1^. Mp: 231.9–232.6 °C, dec HPLC purity: 95.6%. See SI Figure S7 for NMR spectra of compound **28** and Figure S21 for the HPLC chromatogram.

#### 2-((*S*)-4-(4-Chlorophenyl)-2,3,9-trimethyl-6*H*-thieno[3,2-*f*][1,2,4]triazolo[4,3-*a*][1,4]diazepin-6-yl)-*N*-(2-((5-((2-(2,6-dioxopiperidin-3-yl)-1,3-dioxoisoindolin-4-yl)oxy)pentyl)amino)-2-oxoethyl)acetamide (**29**)

The title compound was synthesized following the general procedure D, starting from thalidomide-bearing isocyanide **3**. The crude material was purified by column chromatography using Cy/EtOAc 5:5 and CH_3_CN/MeOH 8:2 as eluents, affording compound **29** (67.1 mg, 70%) as a white solid. ^1^H NMR (400 MHz; (CD_3_)_2_CO): δ 10.08 (br s, 1H), 8.22 (br t, *J* = 6.9 Hz, 1H), 7.77 (t, *J* = 8.4 Hz, 1H), 7.64 (br t, *J* = 5.8 Hz, 1H), 7.54 (d, *J* = 8.5 Hz, 2H), 7.47–7.39 (m, 4H), 5.12 (ddd, *J*_*s*_ = 12.6, 5.4, 2.3 Hz, 1H), 4.68 (dd, *J*_*s*_ = 8.6, 6.1 Hz, 1H), 4.22 (t, *J* = 6.3 Hz, 2H), 4.09 (dd, *J*_*s*_ = 18.5, 6.7 Hz, 1H), 3.71 (dd, *J*_*s*_ = 16.8, 5.0 Hz, 1H), 3.58 (ddd, *J*_*s*_ = 15.0, 8.6, 3.4 Hz, 1H), 3.34 (ddd, *J*_*s*_ = 14.9, 6.1, 2.0 Hz, 1H), 3.25 (q, *J* = 6.3 Hz, 2H), 3.01–2.91 (m, 1H), 2.83–2.73 (m, 2H), 2.64 (s, 3H), 2.43 (s, 3H), 2.24–2.19 (m, 1H), 1.83 (quint, *J* = 6.3 Hz, 2H), 1.71 (s, 3H), 1.58–1.49 (m, 4H). ^13^C NMR (101 MHz; (CD_3_)_2_CO): δ 172.1, 170.6, 169.4 (2C), 167.0, 165.6, 163.9, 156.6, 155.8, 150.1, 137.3, 136.7, 135.9, 133.9, 132.5, 131.0, 130.5, 130.3, 130.2, 128.4, 119.5, 117.0, 115.0, 69.1, 54.5, 49.2, 42.7, 38.6, 38.4, 31.1, 28.8, 28.3, 22.7, 22.4, 13.6, 12.1, 10.9. HRMS (ESI) *m*/*z* (M + Na)^+^ calcd for C_39_H_39_ClN_8_O_7_SNa 821.2238, found 821.2231. IR (neat): ν̃ = 3402, 2927, 1706, 1674, 1365, 1183, 1050, 750, 516, 467 cm^–1^. Mp: 223.4–225.1 °C, dec HPLC purity: 97.8%. See SI Figure S8 for NMR spectra of compound **29** and Figure S22 for the HPLC chromatogram.

#### 2-((*S*)-4-(4-Chlorophenyl)-2,3,9-trimethyl-6*H*-thieno[3,2-*f*][1,2,4]triazolo[4,3-*a*][1,4]diazepin-6-yl)-*N*-(2-((2-(2-((2-(2,6-dioxopiperidin-3-yl)-1,3-dioxoisoindolin-4-yl)oxy)ethoxy)ethyl)amino)-2-oxoethyl)acetamide (**30**)

The title compound was synthesized following the general procedure D, starting from thalidomide-bearing isocyanide **4**. The crude material was purified by column chromatography using Cy/EtOAc 5:5 and CH_3_CN/MeOH 9:1 as eluents, affording compound **30** (56.7 mg, 59%) as a yellow solid. ^1^H NMR (400 MHz; CD_3_OD): δ 7.72 (t, *J* = 8.4 Hz, 1H), 7.46 (d, *J* = 8.6 Hz, 2H), 7.43–7.40 (m, 3H), 7.39 (d, *J* = 8.4 Hz, 1H), 5.09 (dd, *J*_*s*_ = 12.8, 5.8 Hz, 1H), 4.63 (t, *J* = 7.1 Hz, 1H), 4.33 (t, *J* = 4.1 Hz, 2H), 4.01–3.96 (m, 1H), 3.93–3.91 (m, 1H), 3.89–3.87 (m, 2H), 3.69 (t, *J* = 5.4 Hz, 2H), 3.47–3.44 (m, 4H), 2.88–2.78 (m, 2H), 2.73–2.71 (m, 1H), 2.69 (s, 3H), 2.43 (s, 3H), 2.13–2.09 (m, 1H), 1.68 (s, 3H). ^13^C NMR (101 MHz; CD_3_OD): δ 173.1, 171.8, 169.9, 169.3, 167.2, 164.8, 156.1, 155.8, 150.7, 136.8, 136.6 (2C), 133.5, 133.4, 132.1, 131.8, 130.7, 130.6, 130.0, 128.4, 119.1, 116.8, 115.2, 67.7, 51.0, 37.2, 36.6, 36.1, 34.9, 34.2, 30.8, 28.4, 22.3, 13.0, 11.6, 10.3. HRMS (ESI) *m*/*z* (M + Na)^+^ calcd for C_38_H_37_ClN_8_O_8_SNa 823.2030, found 823.2026. IR (neat): ν̃ = 3328, 2970, 1710, 1656, 1365, 1200, 1050, 747, 529, 466 cm^–1^. Mp: 224.4–226.6 °C, dec. See SI Figure S9 for NMR spectra of compound **30**.

#### 2-((*S*)-4-(4-Chlorophenyl)-2,3,9-trimethyl-6*H*-thieno[3,2-*f*][1,2,4]triazolo[4,3-*a*][1,4]diazepin-6-yl)-*N*-(2-((2-(2-(2-((2-(2,6-dioxopiperidin-3-yl)-1,3-dioxoisoindolin-4-yl)oxy)ethoxy)ethoxy)ethyl)amino)-2-oxoethyl)acetamide (**31**)

The title compound was synthesized following the general procedure D, starting from thalidomide-bearing isocyanide **5**. The crude material was purified by column chromatography using Cy/EtOAc 5:5 and CH_3_CN/MeOH 9:1 as eluents, affording compound **31** (48.6 mg, 58%) as a yellow solid. ^1^H NMR (400 MHz; (CD_3_)_2_CO): δ 10.00 (br s, 1H), 8.04 (br t, *J* = 6.1 Hz, 1H), 7.78 (t, *J* = 8.5 Hz, 1H), 7.58 (br t, *J* = 5.5 Hz, 1H), 7.54 (d, *J* = 8.5 Hz, 2H), 7.50 (d, *J* = 8.5 Hz, 1H), 7.44–7.42 (m, 3H), 5.11 (dd, *J*_*s*_ = 12.4, 5.3 Hz, 1H), 4.66 (t, *J* = 7.3 Hz, 1H), 4.39 (t, *J* = 4.6 Hz, 2H), 4.06 (dd, *J*_*s*_ = 16.8, 6.9 Hz, 1H), 3.90 (t, *J* = 4.6 Hz, 2H), 3.71 (t, *J* = 4.1 Hz, 2H), 3.68–3.66 (m, 1H), 3.60 (t, *J* = 5.4 Hz, 2H), 3.56–3.54 (m, 1H), 3.53–3.51 (m, 2H), 3.44–3.41 (m, 1H), 3.39–3.32 (m, 2H), 2.83–2.73 (m, 3H), 2.64 (s, 3H), 2.44 (s, 3H), 2.25–2.19 (m, 1H), 1.71 (s, 3H). ^13^C NMR (101 MHz; (CD_3_)_2_CO): δ 171.9, 170.4, 169.3 (2C), 166.9, 165.5, 163.8, 156.4, 155.8, 150.0, 137.3, 136.6, 135.9, 133.9, 132.6, 130.9, 130.5, 130.3, 130.2, 128.4, 119.9, 117.2, 115.3, 70.7, 70.1, 69.4, 69.3, 69.2, 54.4, 49.2, 42.7, 38.8, 38.4, 31.1, 22.4, 13.6, 12.1, 10.9. HRMS (ESI) *m*/*z* (M + H)^+^ calcd for C_40_H_42_ClN_8_O_9_S 845.2478, found 845.2465. IR (neat): ν̃ = 3308, 2970, 1709, 1659, 1354, 1198, 1051, 747, 528, 466 cm^–1^. Mp: 201.2–202.9 °C, dec. See SI Figure S10 for NMR spectra of compound **31**.

### General Procedure E for Piperazine-Mediated Split Ugi Reactions **32**–**36**

To a solution of piperazine **10** (0.120 mmol, 1 equiv) in MeOH (500 μL) were added paraformaldehyde **7** (0.120 mmol, 1 equiv), isocyanide **1**–**5** (0.120 mmol, 1 equiv), and carboxylic acid **6** (0.120 mmol, 1 equiv) sequentially. The reaction mixture was heated at reflux for 2 h, the volatile was removed in vacuo, and the crude product was purified by column chromatography using the eluent indicated below.

#### 2-(4-(2-((*S*)-4-(4-Chlorophenyl)-2,3,9-trimethyl-6*H*-thieno[3,2-*f*][1,2,4]triazolo[4,3-*a*][1,4]diazepin-6-yl)acetyl)piperazin-1-yl)-*N*-(3-((2-(2,6-dioxopiperidin-3-yl)-1,3-dioxoisoindolin-4-yl)oxy)propyl)acetamide (**32**)

The title compound was synthesized following the general procedure E, starting from thalidomide-bearing isocyanide **1**. The crude material was purified by column chromatography using CH_3_CN/MeOH 9:1 as eluent, affording compound **32** (68.5 mg, 68%) as a white solid. ^1^H NMR (400 MHz; CDCl_3_): δ 10.12 (br s, 1H), 7.84–7.81 (m, 1H), 7.79–7.72 (m, 1H), 7.50 (d, *J* = 8.5 Hz, 2H), 7.49–7.46 (m, 1H), 7.44–7.42 (m, 2H), 7.42–7.40 (m, 1H), 5.13 (dd, *J*_*s*_ = 12.4, 5.3 Hz, 1H), 4.72 (t, *J* = 6.6 Hz, 1H), 4.33 (t, *J* = 6.0 Hz, 2H), 3.76–3.73 (m, 2H), 3.66–3.60 (m, 2H), 3.51 (q, *J* = 6.6 Hz, 2H), 3.47–3.45 (m, 1H), 3.43–3.41 (m, 1H), 3.04 (s, 2H), 2.93–2.89 (m, 2H), 2.83–2.78 (m, 2H), 2.75–2.73 (m, 1H), 2.62 (s, 3H), 2.51–2.48 (m, 2H), 2.44 (s, 3H), 2.26–2.20 (m, 1H), 2.11–2.08 (m, 2H), 1.70 (s, 3H). ^13^C NMR (101 MHz; (CD_3_)_2_CO, *: refers to the main rotamer): δ 171.9*, 169.4*, 169.3, 168.5, 166.9*, 165.7*, 163.1, 156.4, 155.8*, 149.6, 137.4, 136.8*, 135.8, 133.9*, 132.7, 130.7, 130.4, 130.2 (2C), 128.4, 119.7*, 117.2*, 115.3*, 67.8*, 61.5, 54.6, 53.5, 53.0, 49.3, 45.4, 41.4, 36.0, 35.0, 31.2, 30.7, 22.5*, 13.7, 12.2, 10.9. HRMS (ESI) *m*/*z* (M + H)^+^ calcd for C_41_H_43_ClN_9_O_7_S 840.2689, found 840.2677. IR (neat): ν̃ = 3366, 2927, 1709, 1639, 1357, 1198, 1048, 747, 559, 467 cm^–1^. Mp: 205.1–207.4 °C, dec. See SI Figure S11 for NMR spectra of compound **32**.

#### 2-(4-(2-((*S*)-4-(4-Chlorophenyl)-2,3,9-trimethyl-6*H*-thieno[3,2-*f*][1,2,4]triazolo[4,3-*a*][1,4]diazepin-6-yl)acetyl)piperazin-1-yl)-*N*-(4-((2-(2,6-dioxopiperidin-3-yl)-1,3-dioxoisoindolin-4-yl)oxy)butyl)acetamide (**33**)

The title compound was synthesized following the general procedure E, starting from thalidomide-bearing isocyanide **2**. The crude material was purified by column chromatography using CH_3_CN/MeOH 9:1 as eluent, affording compound **33** (84.0 mg, 82%) as a white solid. ^1^H NMR (400 MHz; (CD_3_)_2_CO): δ 10.16 (br d, *J* = 15.5 Hz, 1H), 7.81–7.74 (m, 2H), 7.51 (d, *J* = 8.3 Hz, 2H), 7.48–7.46 (m, 1H), 7.44–7.41 (m, 3H), 5.12 (dd, *J*_*s*_ = 14.2, 6.9 Hz, 1H), 4.73 (t, *J* = 8.0 Hz, 1H), 4.29 (t, *J* = 6.5 Hz, 2H), 3.79–3.76 (m, 2H), 3.66 (dd, *J*_*s*_ = 16.2, 6.9 Hz, 2H), 3.48–3.46 (m, 1H), 3.43–3.41 (m, 1H), 3.37 (q, *J* = 6.5 Hz, 2H), 3.01 (s, 2H), 2.96–2.91 (m, 2H), 2.82–2.75 (m, 2H), 2.71–2.67 (m, 1H), 2.62 (s, 3H), 2.51–2.48 (m, 2H), 2.44 (s, 3H), 2.24–2.20 (m, 1H), 1.90 (quint, *J* = 6.5 Hz, 2H), 1.78 (quint, *J* = 6.5 Hz, 2H), 1.70 (s, 3H). ^13^C NMR (101 MHz; (CD_3_)_2_CO, *: refers to the main rotamer): δ 172.0, 169.4 (2C)*, 168.6, 167.0*, 165.6*, 163.2, 156.5*, 155.8, 149.7, 137.4, 136.7, 135.8, 133.9, 132.7, 130.8, 130.4, 130.3, 130.2, 128.4, 119.4*, 117.0*, 115.1*, 69.1, 61.5, 54.6, 53.5, 53.1, 49.3*, 45.4, 41.5, 38.1, 35.1, 31.2*, 26.4, 26.2, 22.5, 13.7, 12.2, 11.0. HRMS (ESI) *m*/*z* (M + H)^+^ calcd for C_42_H_45_ClN_9_O_7_S 854.2846, found 854.2837. IR (neat): ν̃ = 3344, 2946, 1711, 1643, 1362, 1197, 1048, 747, 558, 466 cm^–1^. Mp: 229.4–230.0 °C, dec. See SI Figure S12 for NMR spectra of compound **33**.

#### 2-(4-(2-((*S*)-4-(4-Chlorophenyl)-2,3,9-trimethyl-6*H*-thieno[3,2-*f*][1,2,4]triazolo[4,3-*a*][1,4]diazepin-6-yl)acetyl)piperazin-1-yl)-*N*-(5-((2-(2,6-dioxopiperidin-3-yl)-1,3-dioxoisoindolin-4-yl)oxy)pentyl)acetamide (**34**)

The title compound was synthesized following the general procedure E, starting from thalidomide-bearing isocyanide **3**. The crude material was purified by column chromatography using CH_3_CN/MeOH 9:1 as eluent, affording compound **34** (78.1 mg, 75%) as a white solid. ^1^H NMR (400 MHz; CD_3_OD): δ 7.77 (t, *J* = 8.2 Hz, 1H), 7.47 (d, *J* = 8.7 Hz, 2H), 7.45–7.42 (m, 4H), 5.10 (dd, *J*_*s*_ = 13.6, 5.3 Hz, 1H), 4.70 (t, *J* = 6.8 Hz, 1H), 4.26 (t, *J* = 6.0 Hz, 2H), 3.82–3.79 (m, 2H), 3.70 (t, *J* = 6.0 Hz, 2H), 3.61–3.57 (m, 2H), 3.38–3.35 (m, 2H), 3.09 (s, 2H), 2.92–2.80 (m, 2H), 2.76–2.73 (m, 1H), 2.72 (s, 3H), 2.69–2.65 (m, 2H), 2.56–2.53 (m, 2H), 2.47 (s, 3H), 2.13–2.09 (m, 1H), 1.92 (quint, *J* = 6.0 Hz, 2H), 1.72 (s, 3H), 1.68–1.60 (m, 4H). ^13^C NMR (101 MHz; CD_3_OD): δ 173.1, 171.1, 170.0, 169.3, 167.3, 165.9, 164.7, 156.6, 155.8, 150.7, 136.8, 136.6, 136.5, 133.7, 132.1, 131.8, 130.7, 130.6, 130.0, 128.4, 119.1, 116.7, 115.0, 69.0, 60.8, 54.0, 53.0, 52.7, 49.1, 45.3, 41.5, 38.5, 34.6, 30.8, 28.8, 28.3, 23.2, 22.3, 13.0, 11.6, 10.2. HRMS (ESI) *m*/*z* (M + H)^+^ calcd for C_43_H_47_ClN_9_O_7_S 868.3002, found 868.2990. IR (neat): ν̃ = 3363, 2924, 1711, 1640, 1364, 1197, 1047, 747, 558, 466 cm^–1^. Mp: 194.8–196.8 °C, dec HPLC purity: 95.7%. See SI Figure S13 for NMR spectra of compound **34***and*Figure S23 for the HPLC chromatogram.

#### 2-(4-(2-((*S*)-4-(4-Chlorophenyl)-2,3,9-trimethyl-6*H*-thieno[3,2-*f*][1,2,4]triazolo[4,3-*a*][1,4]diazepin-6-yl)acetyl)piperazin-1-yl)-*N*-(2-(2-((2-(2,6-dioxopiperidin-3-yl)-1,3-dioxoisoindolin-4-yl)oxy)ethoxy)ethyl)acetamide (**35**)

The title compound was synthesized following the general procedure E, starting from thalidomide-bearing isocyanide **4**. The crude material was purified by column chromatography using CH_3_CN/MeOH 9:1 as eluent, affording compound **35** (65.7 mg, 63%) as a yellow solid. ^1^H NMR (400 MHz; (CD_3_)_2_CO): δ 9.96 (br s, 1H), 7.85–7.81 (m, 1H), 7.63–7.60 (m, 1H), 7.52 (d, *J* 8.6 Hz, 2H), 7.45–7.37 (m, 4H), 5.12 (dd, *J*_*s*_ = 12.5, 5.5 Hz, 1H), 4.70 (t, *J* = 6.4 Hz, 1H), 4.46–4.43 (m, 2H), 3.94 (t, *J* = 6.0 Hz, 2H), 3.74–3.71 (m, 2H), 3.63–3.59 (m, 2H), 3.56–3.54 (m, 2H), 3.46 (q, *J* = 5.6 Hz, 2H), 3.41–3.89 (m, 1H), 3.37–3.35 (m, 1H), 2.99 (s, 2H), 2.94–2.90 (m, 1H), 2.79–2.77 (m, 2H), 2.75–2.73 (m, 2H), 2.63 (s, 3H), 2.48 (s, 3H), 2.39–2.36 (m, 2H), 2.25–2.20 (m, 1H), 1.74 (s, 3H). ^13^C NMR (101 MHz; DMSO-*d*_6_): δ 173.1, 170.3, 169.6, 168.6, 167.7, 166.2, 163.4, 156.1, 155.7, 150.2, 137.3, 136.9, 135.7, 134.3, 132.7, 131.2, 130.6, 130.4, 130.1, 128.9, 120.0, 117.4, 115.6, 69.8, 69.3, 68.9, 61.4, 54.6, 53.4, 53.0, 51.7, 44.5, 41.6, 38.6, 35.2, 31.0, 23.6, 14.5, 13.1, 11.7. HRMS (ESI) *m*/*z* (M + H)^+^ calcd for C_42_H_45_ClN_9_O_8_S 870.2795, found 870.2788. IR (neat): ν̃ = 3338, 2925, 1712, 1639, 1365, 1173, 1051, 748, 558, 465 cm^–1^. Mp: 173.8–174.5 °C, dec. See SI Figure S14 for NMR spectra of compound **35**.

#### 2-(4-(2-((*S*)-4-(4-Chlorophenyl)-2,3,9-trimethyl-6*H*-thieno[3,2-*f*][1,2,4]triazolo[4,3-*a*][1,4]diazepin-6-yl)acetyl)piperazin-1-yl)-*N*-(2-(2-(2-((2-(2,6-dioxopiperidin-3-yl)-1,3-dioxoisoindolin-4-yl)oxy)ethoxy)ethoxy)ethyl)acetamide (**36**)

The title compound was synthesized following the general procedure E, starting from thalidomide-bearing isocyanide **5**. The crude material was purified by column chromatography using CH_3_CN/MeOH 9:1 as eluent, affording compound **36** (80.0 mg, 73%) as a yellow solid. ^1^H NMR (400 MHz; (CD_3_)_2_CO): δ 10.02 (br s, 1H), 7.78 (t, *J* = 8.6 Hz, 1H), 7.60 (br t, *J* = 6.1 Hz, 1H), 7.53–7.49 (m, 3H), 7.45–7.42 (m, 3H), 5.11 (dd, *J*_*s*_ = 13.9, 6.7 Hz, 1H), 4.70 (t, *J* = 6.7 Hz, 1H), 4.43 (t, *J* = 4.5 Hz, 2H), 3.95 (t, *J* = 4.5 Hz, 2H), 3.79 (t, *J* = 4.6 Hz, 2H), 3.67–3.62 (m, 5H), 3.58–3.54 (m, 5H), 3.43–3.38 (m, 2H), 2.99 (s, 2H), 2.95–2.93 (m, 2H), 2.79–2.74 (m, 2H), 2.68–2.65 (m, 1H), 2.63 (s, 3H), 2.54–2.49 (m, 2H), 2.45 (s, 3H), 2.25–2.19 (m, 1H), 1.73 (s, 3H). ^13^C NMR (101 MHz; (CD_3_)_2_CO, *: refers to the main rotamer): δ 171.2, 169.2, 169.0, 168.5, 166.9, 165.4, 163.1, 156.5*, 155.8, 149.6, 137.5, 136.6*, 135.8, 134.0, 132.7, 130.7, 130.4, 130.3, 130.2, 128.4, 120.0, 117.3, 115.3, 71.0, 70.2, 69.6, 69.5, 69.3, 61.4, 54.7, 53.4, 53.0, 49.3*, 45.5, 41.5, 38.5, 35.0, 31.1*, 22.4*, 13.6, 12.1, 10.9. HRMS (ESI) *m*/*z* (M + H)^+^ calcd for C_44_H_49_ClN_9_O_9_S 914.3057, found 914.3053. IR (neat): ν̃ = 3351, 2925, 1711, 1643, 1364, 1199, 1051, 748, 558, 466 cm^–1^. Mp 194.5–195.8 °C, dec. See SI Figure S15 for NMR spectra of compound **36**.

#### Synthesis of 2-((4-((2-(2,6-Dioxopiperidin-3-yl)-1,3-dioxoisoindolin-4-yl)oxy)butyl)amino)-2-oxoethyl2-((*S*)-4-(4-chlorophenyl)-2,3,9-trimethyl-6*H*-thieno[3,2-*f*][1,2,4]triazolo[4,3-*a*][1,4]diazepin-6-yl)acetate (**37**)

To a solution of thalidomide-bearing isocyanide **2** (0.240 mmol, 2 equiv) in CH_2_Cl_2_ (1.30 mL) were added formaldehyde **7** (37% aqueous solution, 0.480 mmol, 4 equiv) and (+)-JQ1 carboxylic acid **6** (0.120 mmol, 1 equiv), and the resulting mixture was stirred at 40 °C for 4 h. Then the volatile was removed in vacuo, and the crude product was purified by column chromatography using Cy/EtOAc 1:9 as eluent, affording compound **37** (56.5 mg, 60%) as a white solid. ^1^H NMR (400 MHz; DMSO-*d*_6_): δ 11.10 (br s, 1H), 8.09 (t, *J* = 5.8 Hz, 1H), 7.80 (t, *J* = 6.0 Hz, 1H), 7.53–7.48 (m, 3H), 7.45–7.43 (m, 3H), 5.45 (t, *J* = 5.8 Hz, 1H), 5.08 (dd, *J*_*s*_ = 13.0, 5.3 Hz, 1H), 4.52 (dd, *J*_*s*_ = 24.2, 14.8 Hz, 2H), 4.21 (t, *J* = 5.7 Hz, 2H), 3.57 (dd, *J*_*s*_ = 6.7, 4.0 Hz, 2H), 3.19 (q, *J* = 5.7 Hz, 2H), 2.92–2.83 (m, 1H), 2.61 (s, 3H), 2.57–2.54 (m, 2H), 2.40 (s, 3H), 2.06–2.00 (m, 1H), 1.76 (quint, *J* = 5.7 Hz, 2H), 1.66–1.64 (m, 2H), 1.62 (s, 3H). ^13^C NMR (101 MHz; DMSO-*d*_6_, *: refers to the main rotamer): δ 173.1, 170.6, 170.3, 167.3, 166.9, 165.8, 163.9, 156.5, 155.2, 150.5, 137.5, 137.2, 135.8, 133.8, 132.7, 131.3, 130.7, 130.5, 130.1, 128.9, 120.4, 116.9, 115.7, 69.2, 63.0*, 53.9, 49.3*, 38.4*, 36.9, 31.4*, 26.3, 26.0, 22.5*, 14.4, 13.1, 11.7. HRMS (ESI) *m*/*z* (M + H)^+^ calcd for C_38_H_37_ClN_7_O_8_S 786.2107, found 786.2097. IR (neat): ν̃ = 3370, 2921, 1708, 1554, 1391, 1195, 1048, 747, 559, 467 cm^–1^. Mp: 105.2–107.0 °C, dec HPLC purity: 97.6%. See SI Figure S16 for NMR spectra of compound **37** and Figure S24 for the HPLC chromatogram.

### General Procedure F of Saponification Reaction for **40** and **41**

Isocyanide **38** or **39** (1.00 mmol, 1 equiv) was solubilized in dry MeOH (1.00 mL) under nitrogen, and the resulting mixture was cooled to 0 °C. Then KOH (1.00 mmol, 1 equiv) was added, and the reaction was stirred for 30 min. The mixture was allowed to reach room temperature and stirred for additional 2 h. Then the volatile solvent was removed in vacuo, and the obtained products **40** or **41** was used, without further purification and characterization, in the following step.

### General Procedure G for the Synthesis of Isocyanides **43** and **44**

To a solution of **40** or **41** (0.500 mmol, 1 equiv) in dry DMF were sequentially added (2*S*,4*R*)-1-((*S*)-2-amino-3,3-dimethylbutanoyl)-4-hydroxy-*N*-(4-(4-methylthiazol-5-yl)benzyl)pyrrolidine-2-carboxamide hydrochloride **42** (0.500 mmol, 1 equiv), DIPEA (2.00 mmol, 4 equiv), and HATU (0.630 mmol, 1.25 equiv) under nitrogen. The reaction was stirred at room temperature for 7 h. EtOAc was added, and the organic layer was washed with water (×3), dried over sodium sulfate, and evaporated. The crude material was purified by column chromatography using the eluent indicated below.

#### (2*S*,4*R*)-4-Hydroxy-1-((*S*)-2-(2-isocyanoacetamido)-3,3-dimethylbutanoyl)-*N*-(4-(4-methyl thiazol-5-yl)benzyl)pyrrolidine-2-carboxamide (**43**)

The title compound was synthesized following the general procedure G, using isocyanide **40**. The crude material was purified by column chromatography using Cy/EtOAc 1:9 as eluent, affording compound **43** (99.4 mg, 40%) as a yellow solid. ^1^H NMR (400 MHz; CD_3_OD): δ 8.94 (s, 1H), 8.18 (br s, 1H), 7.91 (br d, *J* = 8.6 Hz, 1H), 7.48 (d, *J* = 4.2 Hz, 2H), 7.44 (d, *J* = 4.2 Hz, 2H), 4.67 (t, *J* = 8.0, 1H), 4.58–4.54 (m, 1H), 4.52 (s, 2H), 4.38 (dd, *J*_*s*_ = 15.1, 5.5 Hz, 1H), 3.98 (d, *J* = 11.1 Hz, 1H), 3.91 (dd, *J*_*s*_ = 11.1, 3.8 Hz, 1H), 3.83 (s, 2H), 2.50 (s, 3H), 2.25–2.21 (m, 1H), 2.13–2.07 (m, 1H), 1.06 (s, 9H). ^13^C NMR (101 MHz; CD_3_OD, *: refers to the main rotamer): δ 173.0*, 170.6*, 169.3, 162.8, 147.5, 138.9 (2C), 132.0, 130.0, 129.0, 127.6, 70.0, 59.4, 57.7*, 56.6, 42.3, 40.5*, 37.5*, 35.4*, 25.6, 14.3. HRMS (ESI) *m*/*z* (M-H)^−^ calcd for C_25_H_30_N_5_O_4_S 496.2013, found 496.2027. IR (neat): ν̃ = 3292, 2922, 2159, 1664, 1535, 1416, 1229, 1084, 728, 550 cm^–1^. Mp: 63.1–64.6 °C.

#### (2*S*,4*R*)-4-Hydroxy-1-((*S*)-2-(4-isocyanobutanamido)-3,3-dimethylbutanoyl)-*N*-(4-(4-methylthiazol-5-yl)benzyl)pyrrolidine-2-carboxamide (**44**)

The title compound was synthesized following the general procedure G, using isocyanide **41**. The crude material was purified by column chromatography using CH_3_CN/MeOH 9:1 as eluent, affording compound **44** (86.7 mg, 33%) as a white solid. ^1^H NMR (400 MHz; CDCl_3_): δ 8.68 (s, 1H), 7.36 (d, *J* = 8.3 Hz, 2H), 7.32 (d, *J* = 8.3 Hz, 2H), 6.70 (br d, *J* = 8.8 Hz, 1H), 4.67 (t, *J* = 8.0, 1H), 4.56 (d, *J* = 8.9 Hz, 1H), 4.54–4.48 (m, 2H), 4.34 (dd, *J*_*s*_ = 15.1, 5.4 Hz, 1H), 3.99 (d, *J* = 11.4 Hz, 1H), 3.67 (dd, *J*_*s*_ = 11.2, 3.7 Hz, 1H), 3.45–3.38 (m, 2H), 2.50 (s, 3H), 2.41 (t, *J* = 7.4 Hz, 2H), 2.34–2.28 (m, 1H), 2.14–2.10 (m, 1H), 1.94 (quint, *J* = 7.4 Hz, 2H), 0.96 (s, 9H). HRMS (ESI) *m*/*z* (M + H)^+^ calcd for C_27_H_36_N_5_O_4_S 526.2483, found 526.2481. IR (neat): ν̃ = 3295, 2953, 2148, 1621, 1531, 1416, 1224, 1085, 850, 569 cm^–1^. Mp: 65.7–66.8 °C.

#### (2*S*,4*R*)-1-((*S*)-2-(2-(2-(2-((*S*)-4-(4-Chlorophenyl)-2,3,9-trimethyl-6*H*-thieno[3,2-*f*][1,2,4]triazolo[4,3-*a*][1,4]diazepin-6-yl)-*N*-methylacetamido)acetamido)acetamido)-3,3-dimethylbutanoyl)-4-hydroxy-*N*-(4-(4-methylthiazol-5-yl)benzyl)pyrrolidine-2-carboxamide (**45**)

To a solution of methylamine **8** (40% aqueous solution, 0.240 mmol, 2 equiv) in MeOH (500 μL) were added formaldehyde **7** (37% aqueous solution, 0.360 mmol, 3 equiv), isocyanide **43** (0.180 mmol, 1.5 equiv), and carboxylic acid **6** (0.120 mmol, 1 equiv) at 0 °C, and the mixture was stirred for 2 h at the same temperature. The volatile solvent was removed in vacuo, and the crude product was purified by column chromatography using CH_3_CN/MeOH 9:1 as eluent, affording compound **45** (45.1 mg, 40%) as a yellow solid. ^1^H NMR (400 MHz; CD_3_OD, *: refers to the main rotamer): δ 8.84 (s, 1H), 8.58 (br s, 1H), 7.47–7.38 (m, 8H), 4.73 (t, *J* = 7.0, 1H), 4.65–4.63 (m, 1H), 4.55 (s, 2H), 4.51 (s, 2H), 4.36 (d, *J* = 15.3 Hz, 1H), 4.25 (d, *J* = 15.3 Hz, 1H), 4.07 (d, *J* = 16.4 Hz, 1H), 4.02–3.98 (m, 1H), 3.92–3.85 (m, 1H), 3.80 (d, *J* = 3.9 Hz, 1H), 3.76 (dd, *J*_*s*_ = 14.5, 7.0 Hz, 1H), 3.64 (dd, *J*_*s*_ = 16.1, 7.0 Hz, 1H), 3.37 (s, 3H), 2.70 (s, 3H), 2.47 (s, 3H), 2.44 (s, 3H), 2.19–2.10 (m, 2H), 1.69 (s, 3H)*, 1.00 (s, 9H)*. ^13^C NMR (101 MHz; CD_3_OD, *: refers to the main rotamer): δ 173.0*, 171.9*, 170.5*, 170.4*, 169.8*, 165.0, 159.8, 155.9*, 150.8*, 147.6, 138.9, 136.8, 136.6*, 132.1, 132.0, 131.8*, 130.7*, 130.6, 130.1, 130.0*, 129.0, 128.4*, 127.6*, 69.7, 59.4, 57.7*, 56.5*, 53.9*, 52.5, 51.0, 42.3*, 37.5, 36.1, 35.5*, 34.8*, 25.6, 14.4, 13.0*, 11.5, 10.2*. HRMS (ESI) *m*/*z* (M + H)^+^ calcd for C_46_H_54_ClN_10_O_6_S_2_ 941.3352, found 941.3347. IR (neat): ν̃ = 3294, 2925, 1626, 1532, 1415, 1348, 1194, 747, 548, 445 cm^–1^. Mp: 114.6–116.0 °C. See SI Figure S17 for NMR spectra of compound **45**.

### General Procedure H for Piperazine-Mediated Split Ugi Reactions **46** and **47**

To a solution of piperazine **10** (0.120 mmol, 1 equiv) in MeOH (500 μL) were added paraformaldehyde **7** (0.120 mmol, 1 equiv), isocyanide **43** or **44** (0.240 mmol, 2 equiv), and carboxylic acid **6** (0.120 mmol, 1 equiv) sequentially. The reaction mixture was heated at reflux for 2 h, the volatile solvent was removed in vacuo, and the crude product was purified by column chromatography using the eluent indicated below.

#### (2*S*,4*R*)-1-((*S*)-2-(2-(2-(4-(2-((*S*)-4-(4-Chlorophenyl)-2,3,9-trimethyl-6*H*-thieno[3,2-*f*][1,2,4]triazolo[4,3-*a*][1,4]diazepin-6-yl)acetyl)piperazin-1-yl)acetamido)acetamido)-3,3-dimethylbutanoyl)-4-hydroxy-*N*-(4-(4-methylthiazol-5-yl)benzyl)pyrrolidine-2-carboxamide (**46**)

The title compound was synthesized following the general procedure H, using (2*S*,4*R*)-4-hydroxy-1-((*S*)-2-(2-isocyanoacetamido)-3,3-dimethylbutanoyl)-*N*-(4-(4-methylthiazol-5-yl)benzyl) pyrrolidine-2-carboxamide **43**. The crude material was purified by column chromatography using CH_3_CN/MeOH 8:2 as eluent, affording compound **46** (50.2 mg, 42%) as a yellow solid. ^1^H NMR (400 MHz; CD_3_OD): δ 8.86 (s, 1H), 8.57 (br s, 1H), 7.45–7.43 (m, 2H), 7.42–7.39 (m, 4H), 7.37 (d, *J* = 8.2 Hz, 2H), 4.69 (t, *J* = 8.0, 1H), 4.68–4.66 (m, 1H), 4.63–4.61 (m, 1H), 4.59–4.56 (m, 1H), 4.52 (s, 2H), 4.36 (dd, *J*_*s*_ = 15.1, 5.5 Hz, 1H), 4.01 (d, *J* = 3.4 Hz, 1H), 3.90 (d, *J* = 11.1 Hz, 1H), 3.84–3.82 (m, 4H), 3.79–3.75 (m, 1H), 3.74–3.66 (m, 2H), 3.58 (d, *J* = 6.9 Hz, 2H), 3.17–3.15 (m, 2H), 2.70 (s, 3H), 2.62–2.59 (m, 2H), 2.46 (s, 3H), 2.45 (s, 3H), 2.27–2.22 (m, 1H), 2.14–2.09 (m, 1H), 1.71 (s, 3H), 1.06 (s, 9H). ^13^C NMR (101 MHz; CD_3_OD): δ 173.1, 172.0, 170.5, 169.7, 169.3, 168.1, 164.7, 155.8, 150.7, 147.6, 138.9, 136.8, 136.5, 132.1, 132.0, 131.8, 130.7 (2C), 130.1, 129.9, 128.9, 128.4, 127.6, 69.7, 60.7, 59.5, 57.6, 56.6, 54.0, 53.0, 52.7, 45.4, 42.3, 41.8, 41.6, 37.6, 35.6, 34.6, 25.6, 14.5, 13.0, 11.6, 10.2. HRMS (ESI) *m*/*z* (M + H)^+^ calcd for C_49_H_59_ClN_11_O_6_S_2_ 996.3774, found 996.3765. IR (neat): ν̃ = 3293, 2921, 1625, 1528, 1417, 1228, 1088, 731, 548, 444 cm^–1^. Mp: 183.3–184.8 °C. See SI Figure S18 for NMR spectra of compound **46**.

#### (2*S*,4*R*)-1-((*S*)-2-(4-(2-(4-(2-((*S*)-4-(4-Chlorophenyl)-2,3,9-trimethyl-6*H*-thieno[3,2-*f*][1,2,4]triazolo[4,3-*a*][1,4]diazepin-6-yl)acetyl)piperazin-1-yl)acetamido)butanamido)-3,3-dimethylbutanoyl)-4-hydroxy-*N*-(4-(4-methylthiazol-5-yl)benzyl)pyrrolidine-2-carboxamide (**47**)

The title compound was synthesized following the general procedure H, using (2*S*,4*R*)-4-hydroxy-1-((*S*)-2-(4-isocyanobutanamido)-3,3-dimethylbutanoyl)-*N*-(4-(4-methylthiazol-5-yl)benzyl) pyrrolidine-2-carboxamide **44**. The crude material was purified by column chromatography using CH_3_CN/MeOH 8:2 as eluent, affording compound **47** (107 mg, 87%) as a white solid. ^1^H NMR (400 MHz; CD_3_OD): δ 8.87 (s, 1H), 7.47–7.44 (m, 4H), 7.43–7.39 (m, 4H), 4.70 (t, *J* = 6.9 Hz, 1H), 4.66–4.64 (m, 1H), 4.61 (t, *J* = 8.3 Hz, 1H), 4.52 (s, 2H), 4.36 (d, *J* = 15.5 Hz, 1H), 3.94 (d, *J* = 11.0 Hz, 1H), 3.84–3.82 (m, 3H), 3.73–3.70 (m, 2H), 3.64 (d, *J* = 9.1 Hz, 1H), 3.60–3.58 (m, 1H), 3.30–3.28 (m, 2H), 3.10 (s, 2H), 2.70 (s, 3H), 2.68–2.65 (m, 2H), 2.55 (t, *J* = 5.1 Hz, 2H), 2.47 (s, 3H), 2.45 (s, 3H), 2.37–2.32 (m, 2H), 2.25–2.21 (m, 1H), 2.13–2.10 (m, 1H), 1.85 (quint, *J* = 7.8 Hz, 2H), 1.70 (s, 3H), 1.07 (s, 9H). ^13^C NMR (101 MHz; CD_3_OD): δ 173.8, 173.1, 171.3, 170.9, 169.3, 164.7, 155.8, 151.4, 150.7, 147.6, 138.8, 136.8, 136.5, 132.1, 132.0, 131.8, 130.7, 130.6, 130.0 (2C), 128.9, 128.4, 127.6, 69.7, 60.8, 59.4, 57.8, 56.6, 54.0, 53.0, 52.7, 45.3, 42.3, 41.5, 38.2, 37.6, 35.1, 34.6, 32.5, 25.7, 25.5, 14.5, 13.0, 11.6, 10.2. HRMS (ESI) *m*/*z* (M + H)^+^ calcd for C_51_H_63_ClN_11_O_6_S_2_ 1024.4087, found 1024.4085. IR (neat): ν̃ = 3302, 2921, 1628, 1526, 1416, 1227, 1088, 803, 549, 445 cm^–1^. Mp: 164.3–165.5 °C. See SI Figure S19 for NMR spectra of compound **47**.

### Synthesis of Isocyanide **18** on a Multigram Scale

Amino alcohol **13** (2.10 g, 20.0 mmol, 1 equiv) and ethyl formate (13.0 mL) were heated under reflux for 6 h. The volatile solvent was removed in vacuo. The corresponding formamide (2.62 g, 20.0 mmol, 1 equiv) was solubilized in dry CH_2_Cl_2_ (30.0 mL) under nitrogen and, after adding TEA (16.7 mL, 120 mmol, 6 equiv), the reaction mixture was cooled at 0 °C. TsCl (11.4 g, 60.0 mmol, 3 equiv) was added, and the reaction was stirred at room temperature for 5 h. The reaction mixture was quenched with a saturated aqueous Na_2_CO_3_ solution and stirred under cooling at 0 °C for 30 min. Water was added, and the aqueous phase was extracted with CH_2_Cl_2_ (×3). The organic layer was dried over sodium sulfate and evaporated. The crude product was purified by silica gel column chromatography using PE/EtOAc 9:1, affording compound **18** (3.15 g, 59%) as a yellow oil.

### Synthesis of Isocyanide-Based Library of CRBN-Recruiting Anchor **3** on a Multigram Scale

To a solution of thalidomide derivative **21** (1.65 g, 6.00 mmol, 1 equiv) in dry DMF (25.5 mL) was added NaHCO_3_ (756 mg, 9.00 mmol, 1.5 equiv) under nitrogen, and the reaction mixture was heated at 65 °C. After 15 min, a solution of isocyanide **18** (1.92 g, 7.20 mmol, 1.2 equiv) in dry DMF (1.50 mL) was added dropwise and the resulting mixture was stirred at 65 °C overnight. The reaction mixture was diluted with CH_2_Cl_2_ and washed with water (×3). The organic layer was dried over sodium sulfate, and the volatile solvent was removed in vacuo. The crude product was purified by silica gel column chromatography using PE/EtOAc 5:5 as eluent, affording compound **3** (1.88 g, 85%) as a white solid.

### Scaled-up Synthesis of PROTAC **34** by Split Ugi Reaction on a One-Gram Scale

To a solution of piperazine **10** (129 mg, 1.50 mmol, 1 equiv) in MeOH (6.00 mL) were added paraformaldehyde (72.1 mg, 1.50 mmol, 1 equiv), isocyanide **3** (554 mg, 1.50 mmol, 1 equiv), and carboxylic acid **6** (601 mg, 1.50 mmol, 1 equiv) sequentially. The reaction mixture was heated at reflux for 2 h, the volatile solvent was removed in vacuo, and the crude product was purified by silica gel column chromatography using CH_3_CN/MeOH 9:1 as eluent, affording compound **34** (1.08 g, 83%) as a white solid.

### Cell Culturing and Western Blot Analysis

The human breast adenocarcinoma MDA-MB-231 cell line was cultured in DMEM (Sigma-Aldrich) supplemented with 10% fetal bovine serum (FBS), 1% glutamine, and 1% penicillin/streptomycin. For Western blot analysis, 15 × 10^5^ cells/well were plated into 12-well plates containing DMEM complete growth medium, to be treated with the synthesized compounds, at the indicated concentrations, for different time lengths. After treatments, cells were scraped and lysed to obtain protein extract using RIPA lysis buffer supplemented with protease (PIC, Merck-Millipore) and phosphatase inhibitors (NaF 1 M, Na_3_VO_4_ 1 M, PMSF 100 nM, Sigma-Aldrich). Cells lysates were centrifuged at 13 000 rpm for 10 min at 4 °C to remove membranes and insoluble fractions. Protein quantification of the supernatant fraction was performed with Braford protein assay (Sigma-Aldrich). Thirty milligram aliquots of protein extracts were used for 12% SDS-PAGE. After transfer into nitrocellulose membranes, BRD4 and tubulin protein levels were evaluated by immunoblotting using a rabbit polyclonal anti-BRD4 (Bethyl Laboratories) and a mouse monoclonal α-tubulin (Sigma-Aldrich) IgG as primary antibodies and an antirabbit or antimouse HRP-conjugated secondary antibody (Bio-Rad Laboratories). Protein levels were detected by chemiluminescent conversion of the HRP substrate LumiGLO (Thermo Fisher Scientific) using the ChemiDoc imaging system (Bio-Rad). Band densitometry was assessed, normalized to α-tubulin immunoreactive bands, and reported as percentage of the 0.3% DMSO control lane.

### Thermodynamic Solubility

Saturated solutions were prepared by dissolving the tested compounds in 0.1 M phosphate-buffered saline (pH = 7.4) or 0.01 N HCl solutions and sonicated for 30 s. After equilibration on an orbital shaker at 25 °C for 2 h, the solutions were centrifuged at 13 000 rpm for 5 min and filtered through a 0.2 μm regenerated cellulose (RC) syringe filter. A 400 μL aliquot of each filtrate was diluted with 100 μL of acetonitrile and analyzed by LC-UV. When the measured peak area was out of the calibration range (5–100 μM), the sample was further diluted in water–acetonitrile 1:1 mixture.

### Metabolic Stability

Mouse liver microsomes (MLM) (pooled male mouse CD-1, protein concentration: 20 mg/mL) were purchased from Corning B.V. Life Sciences (Amsterdam, The Netherlands). The standard incubation mixture (250 μL final volume) was carried out in a 50 mM Tris (tris[hydroxymethyl]aminomethane) buffer (pH 7.4) containing 150 mM KCl, 1.5 mM, 3.3 mM MgCl_2_, 1.3 mM NADPNa_2_, 3.3 mM glucose 6-phosphate, 0.4 units/mL glucose 6-phosphate dehydrogenase, acetonitrile as cosolvent (1% of total volume), and the substrate (50 μM). After pre-equilibration of the mixture, an appropriate volume of MLM suspension was added to give a final protein concentration of 1.0 mg/mL. The mixture was shaken for 60 min at 37 °C. Control incubations were carried out without the presence of an NADPH-regenerating system or microsomes. Each incubation was stopped by the addition of 250 μL of ice-cold acetonitrile, vortexed, and centrifuged at 13 000 rpm for 5 min. The supernatants were analyzed by LC-UV.

## Supporting Information Available

^1^H and ^13^C NMR spectra of final compounds; LC-UV methods for determination of purity of selected active compounds, aqueous solubility, and metabolic stability; HPLC chromatograms (PDF)
Molecular formula strings (CSV)

## Abbreviations Used

2-CR: two-component reaction
AR: androgen receptor
BCL-x_L_: B-cell lymphoma-extra large
BET: bromodomain and extraterminal domain
CRBN: cereblon
D2B: direct-to-biology
DIPEA: *N*,*N*-diisopropylethylamine
DMEM: Dulbecco′s Modified Eagle′s Medium
EtOAc: ethyl acetate
ER: estrogen receptor
FBS: fetal bovine serum
HATU: 1-[bis(dimethylamino)methylene]-1*H*-1,2,3-triazolo[4,5-*b*]pyridinium 3-oxide hexafluorophosphate
IMiD: immunomodulatory imide drug
MCR: multicomponent reaction
MDA-MB-231 Cells: MD Anderson-metastatic breast-231 cells
MeOH: methanol
MLM: mouse liver microsomes
PE: petroleum ether
POI: protein of interest
PROTAC: Proteolysis TArgeting Chimera
RC: regenerate cellulose
RIPA: radioimmunoprecipitation assay
S_N_Ar: nucleophilic aromatic substitution
TC: ternary complex
TEA: triethylamine
TFE: trifluoroethanol
TsCl: tosyl chloride
VHL: von Hippel-Lindau.
