# Supplementary material for: A Versatile and Sustainable Multicomponent Platform for the Synthesis of Protein Degraders: Proof-of-Concept Application to BRD4-Degrading PROTACs
Source: J Med Chem. 2022 Nov 2;65(22):15282–99. doi: 10.1021/acs.jmedchem.2c01218 (PMC9706574; doi:10.1021/acs.jmedchem.2c01218)
Supplement: Supplementary file 1 — jm2c01218_si_001.pdf [file jm2c01218_si_001.pdf]

SUPPORTING INFORMATION

A Versatile and Sustainable Multicomponent Platform for the Synthesis of Protein Degraders: Proof-of-Concept Application to BRD4-Degrading PROTACs

Irene Preet Bhela[1], Alice Ranza[1], Federica Carolina Balestrero[1], Marta Serafini[1§], Silvio Aprile[1], Rita Maria Concetta Di Martino[1], Fabrizio Condorelli[1], and Tracey Pirali[1]*

[1] Department of Pharmaceutical Sciences, Università degli Studi del Piemonte Orientale, Largo Donegani 2, 28100 Novara, Italy

[§] Present Address: Department of Chemistry, Chemistry Research Laboratory, University of Oxford, Mansfield Road, Oxford OX1 3TA, UK

Email: tracey.pirali@uniupo.it

Table of contents

^{1}H and ^{13}C-NMR spectra..S3

LC-UV methods for determination of purity of selected active compounds, aqueous solubility, and metabolic stability S22

HPLC chromatograms..S23

^{1}H and ^{13}C spectra

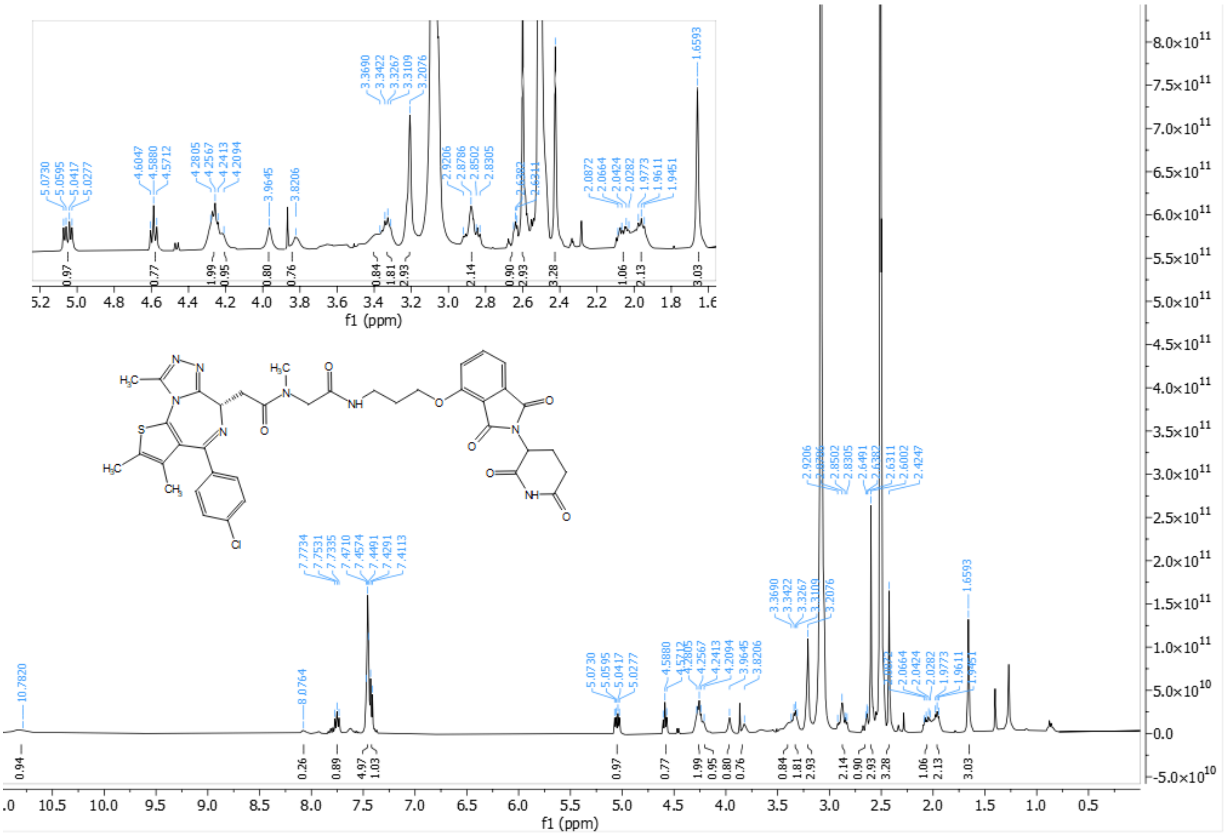

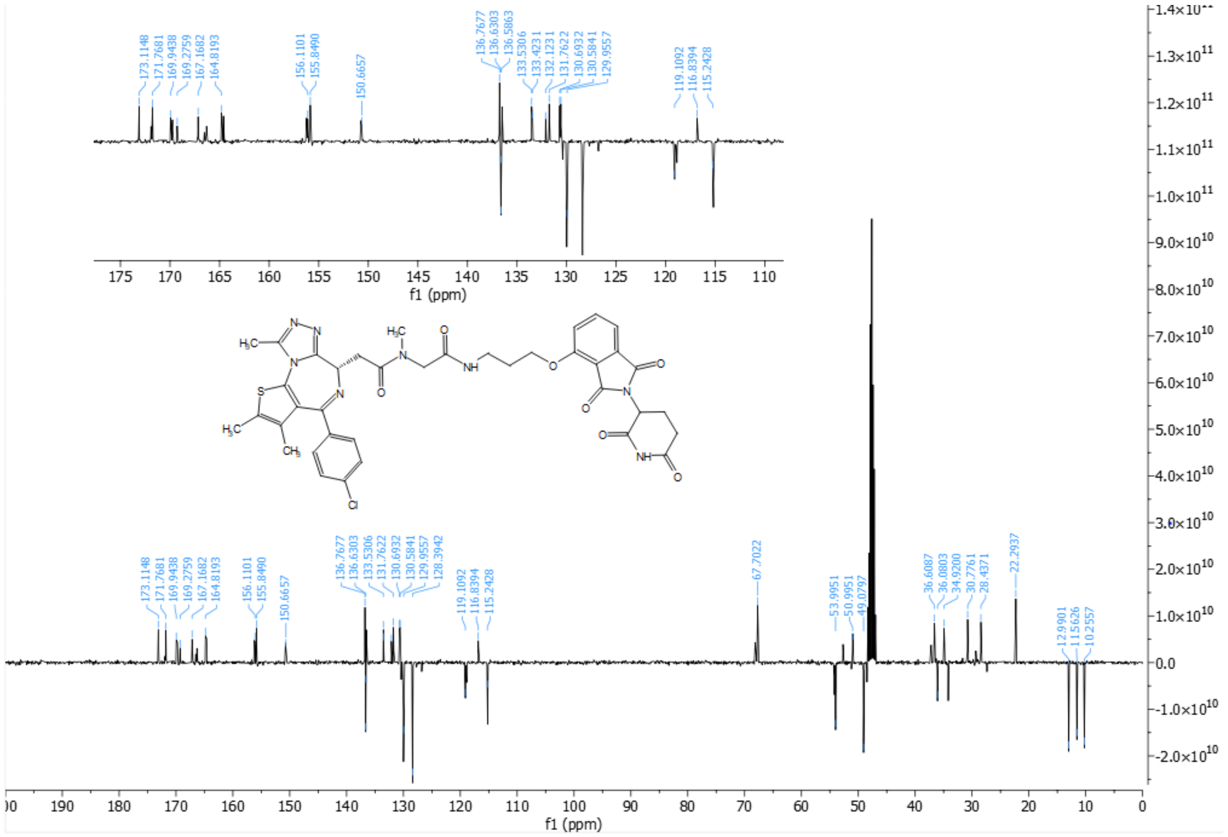

Figure S1. ^{1}H (400 MHz, DMSO-d_6, 353 K, top) and ^{13}C (101 MHz, CD_3OD, bottom) NMR spectra of compound **22**.

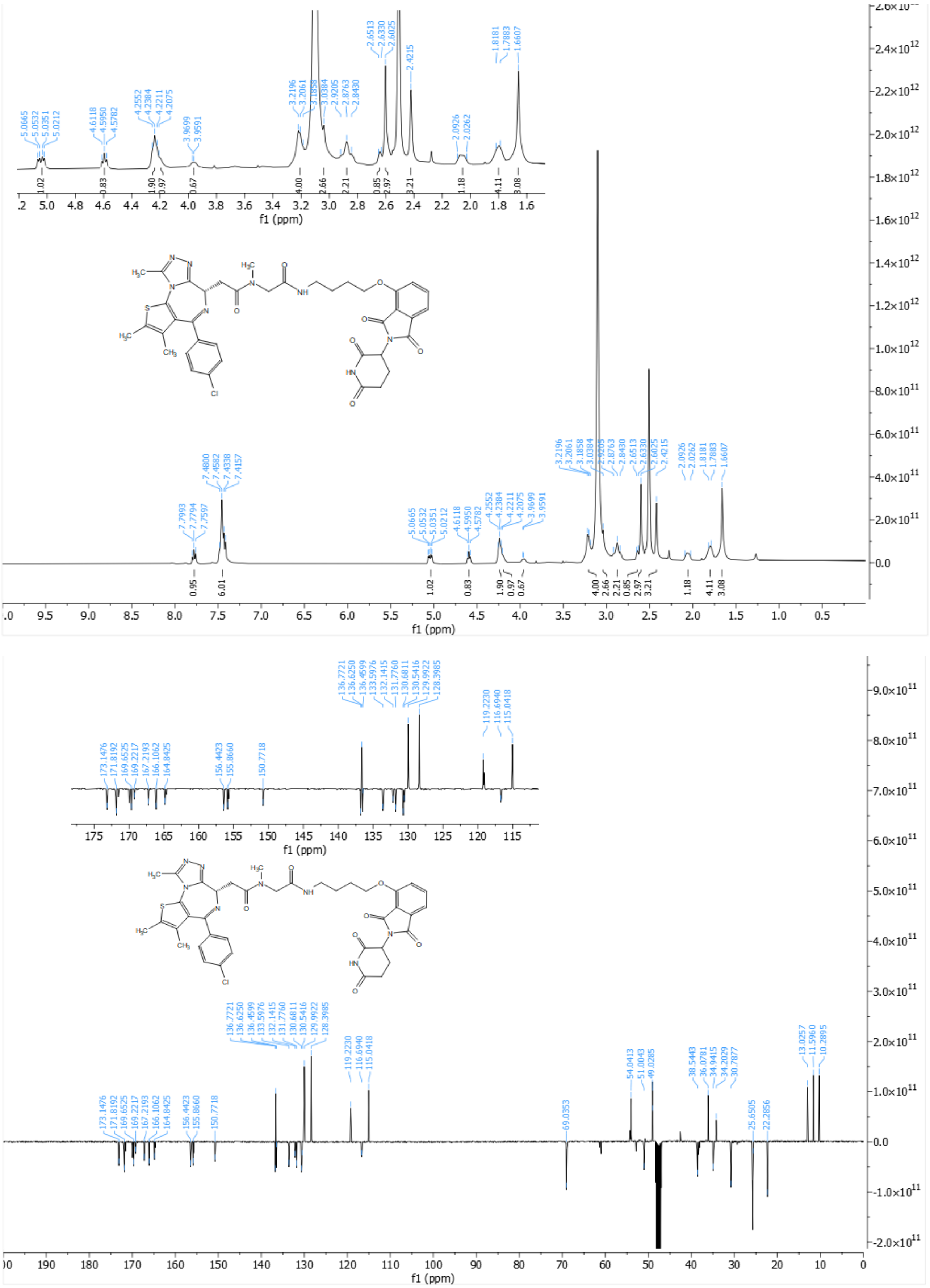

Figure S2. ^{1}H (400 MHz, DMSO-d_6, 353 K, top) and ^{13}C (101 MHz, CD_3OD, bottom) NMR spectra of compound **23**.

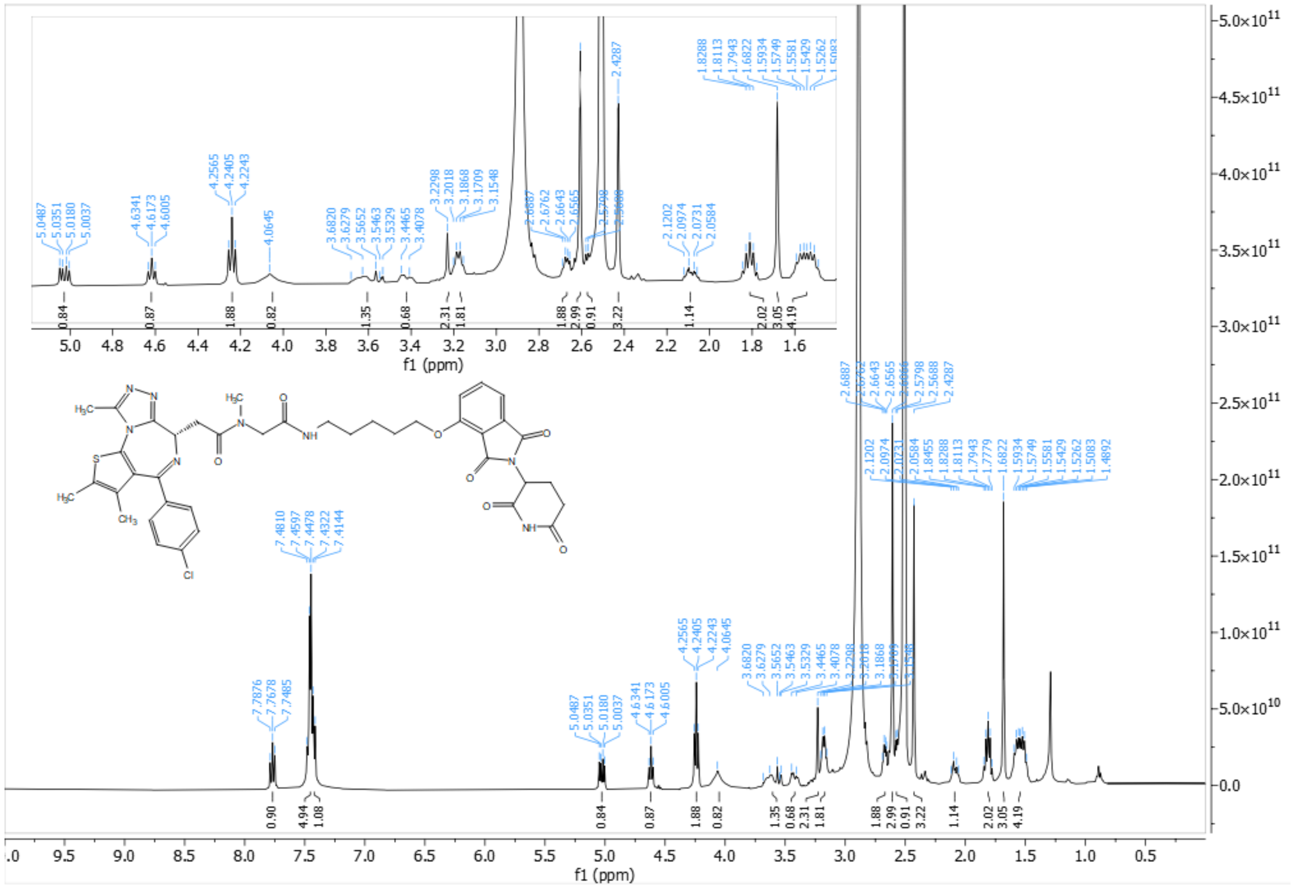

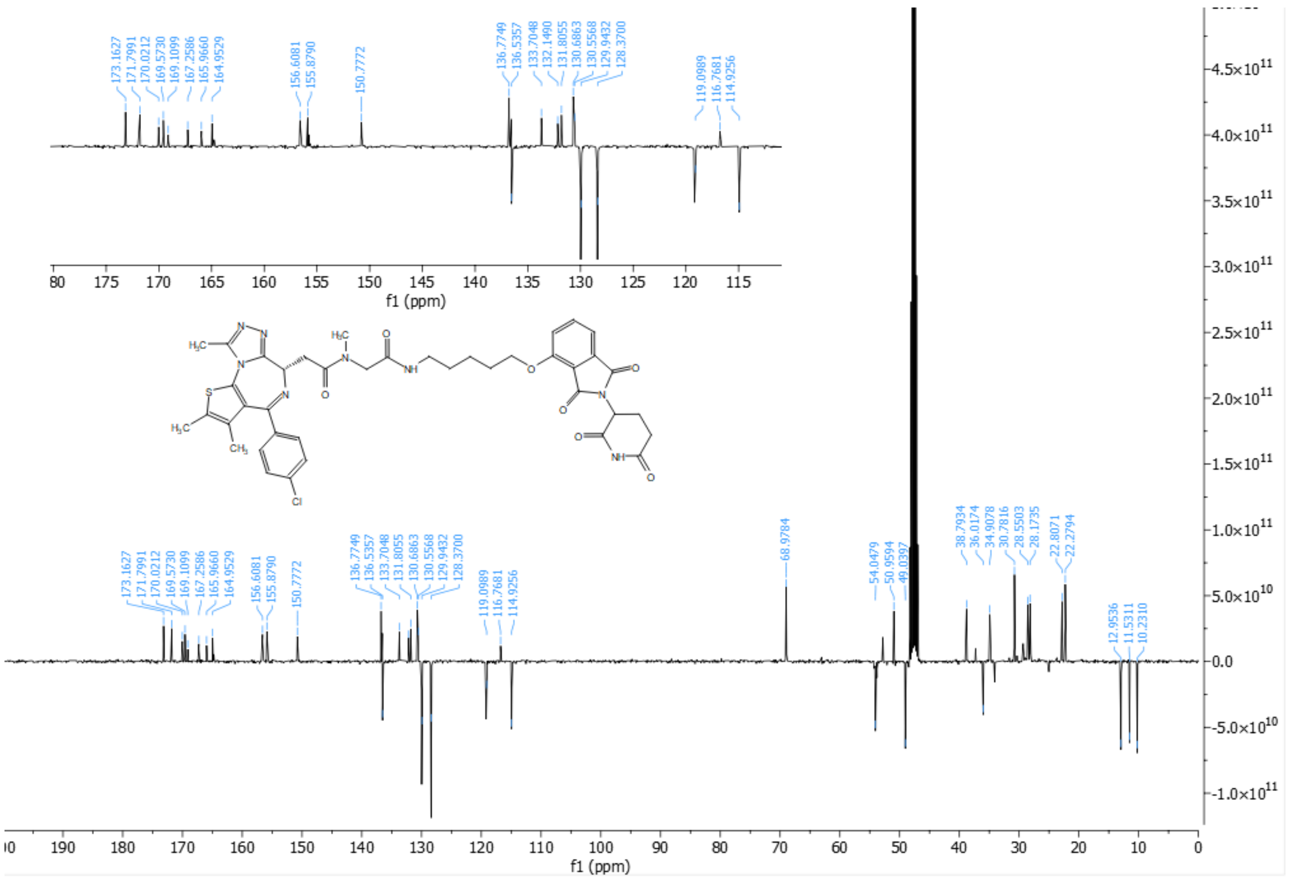

Figure S3. ^{1}H (400 MHz, DMSO-d_6, 353 K, top) and ^{13}C (101 MHz, CD_3OD, bottom) NMR spectra of compound **24**.

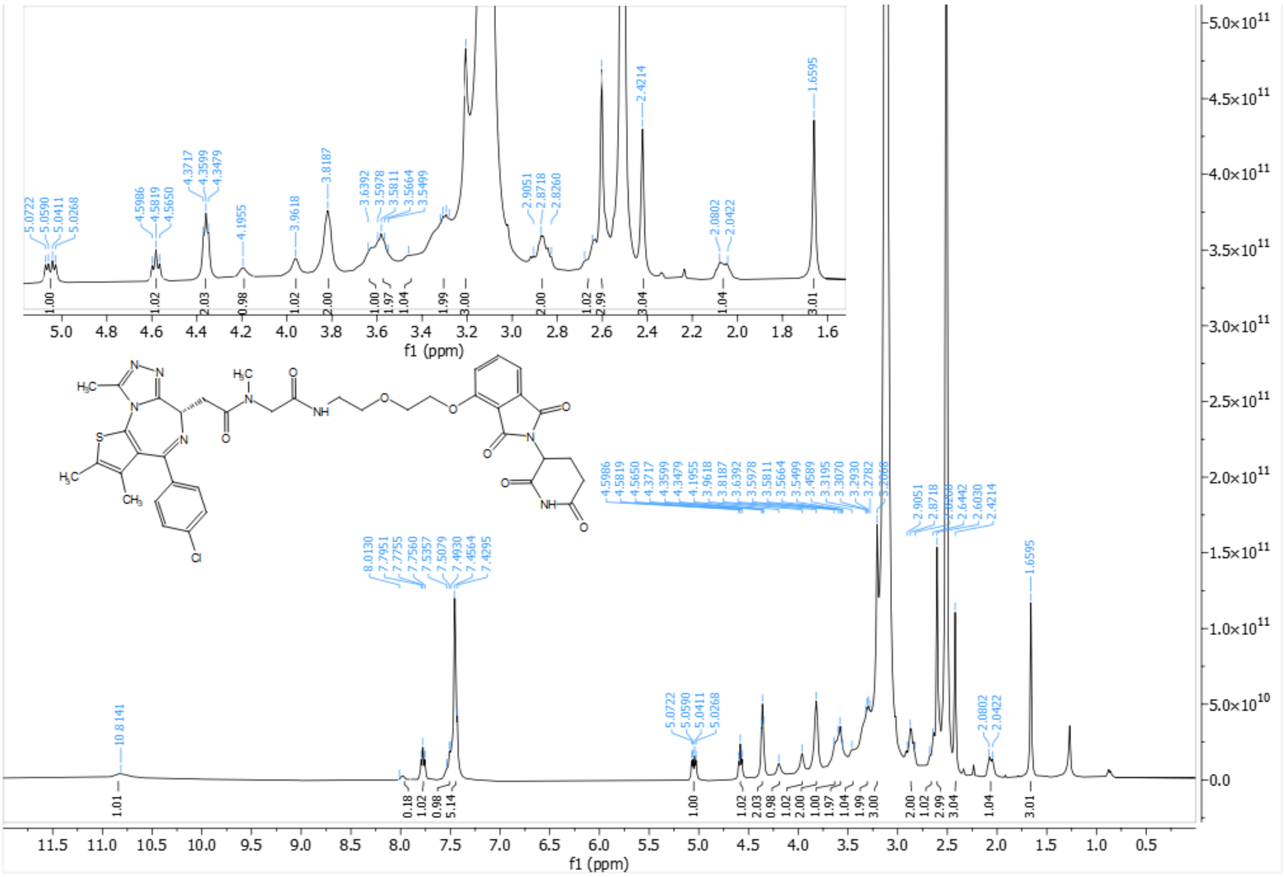

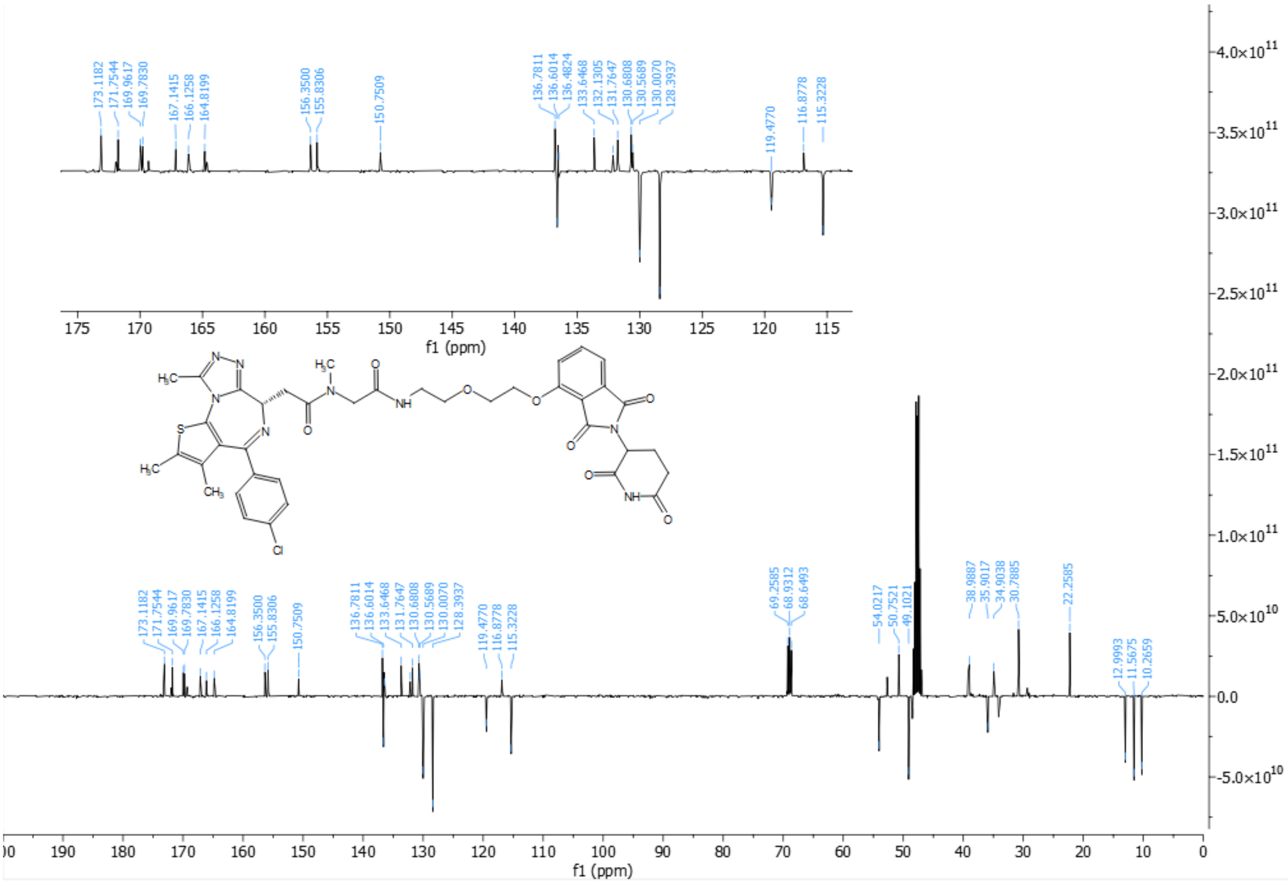

Figure S4. ^{1}H (400 MHz, DMSO-d_6, 353 K, top) and ^{13}C (101 MHz, CD_3OD, bottom) NMR spectra of compound **25**.

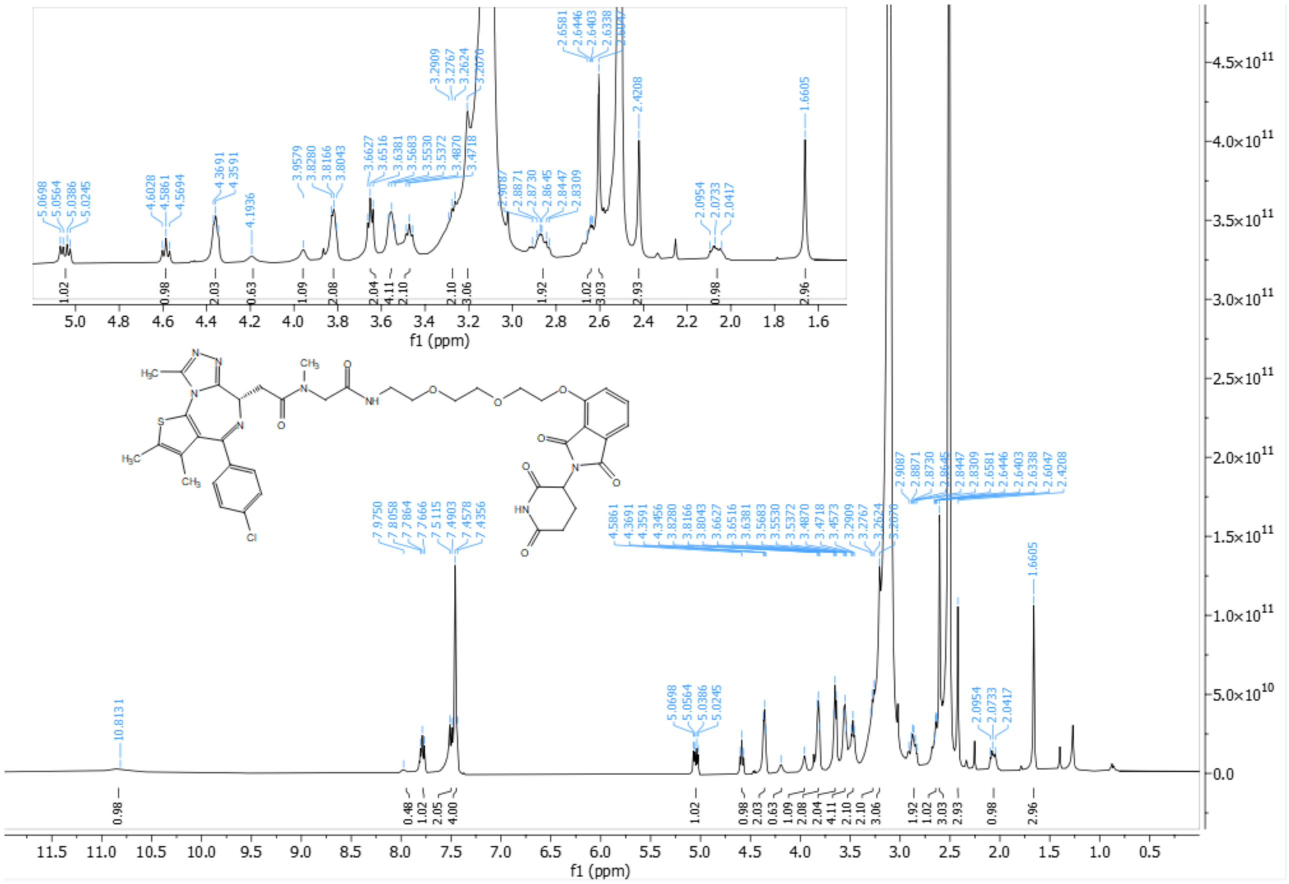

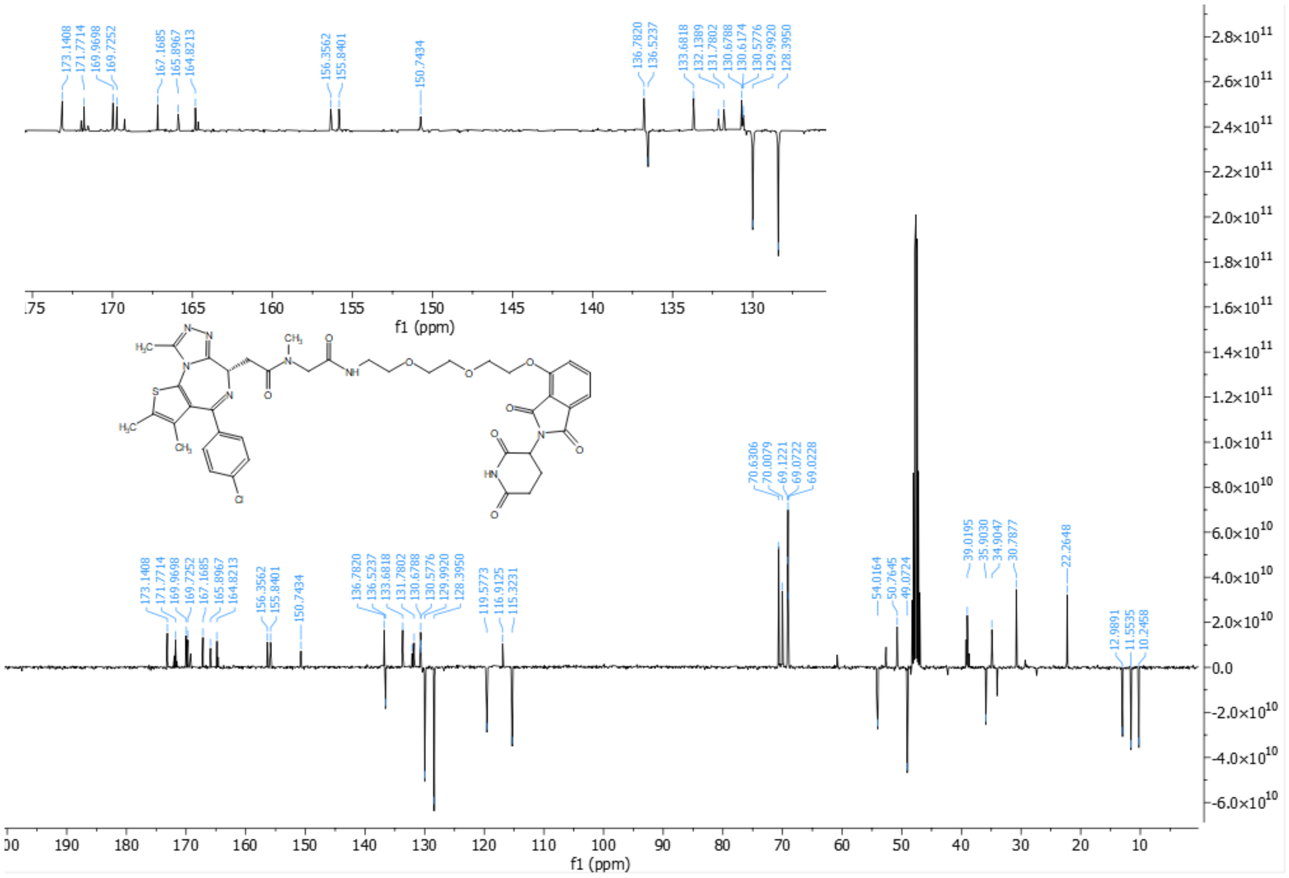

Figure S5. ^{1}H (400 MHz, DMSO-d_6, 353 K, top) and ^{13}C (101 MHz, CD_3OD, bottom) NMR spectra of compound **26**.

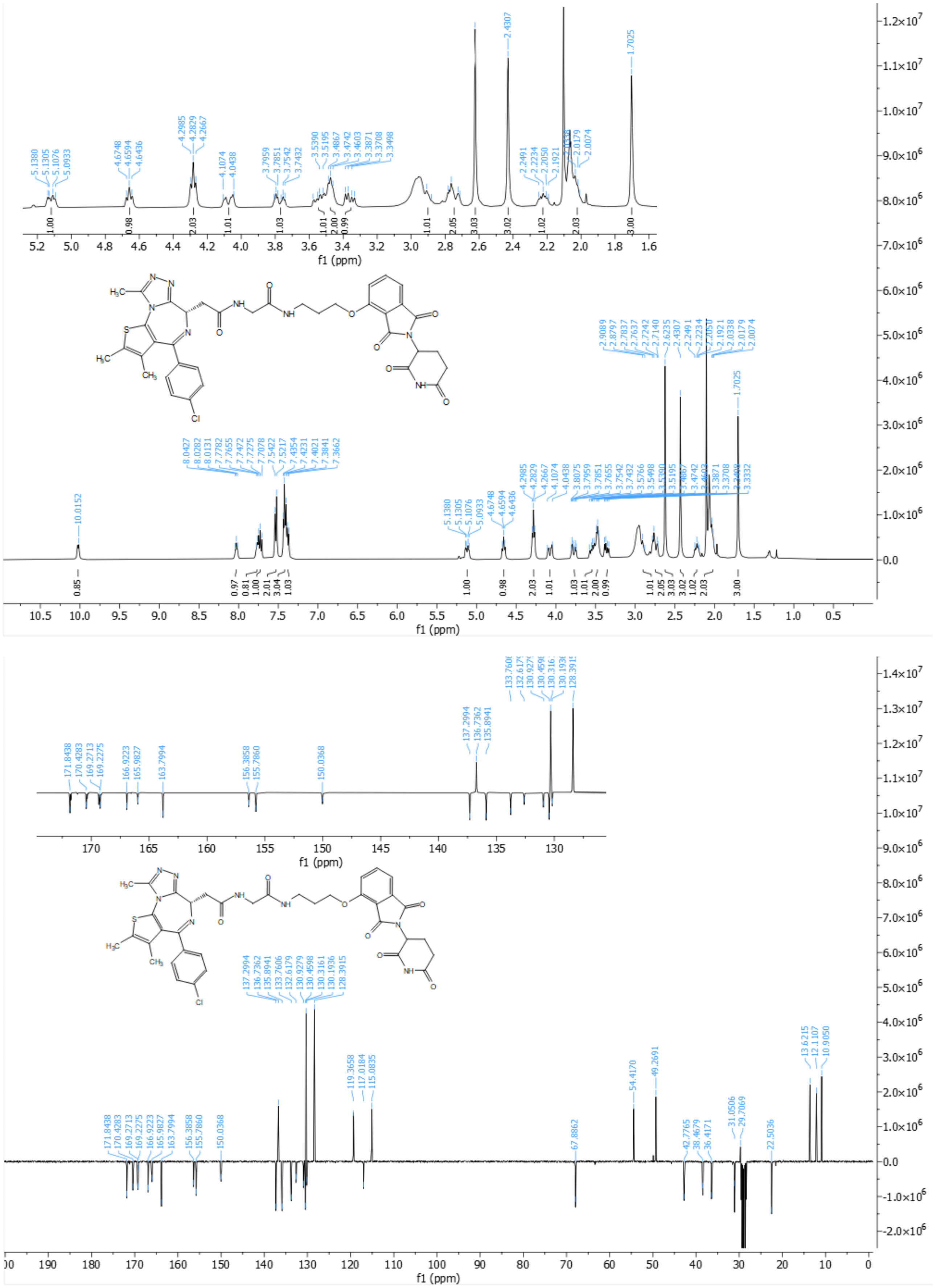

Figure S6. ^{1}H (400 MHz, $(CD_3)_2CO$, top) and ^{13}C (101 MHz, $(CD_3)_2CO$, bottom) NMR spectra of compound **27**.

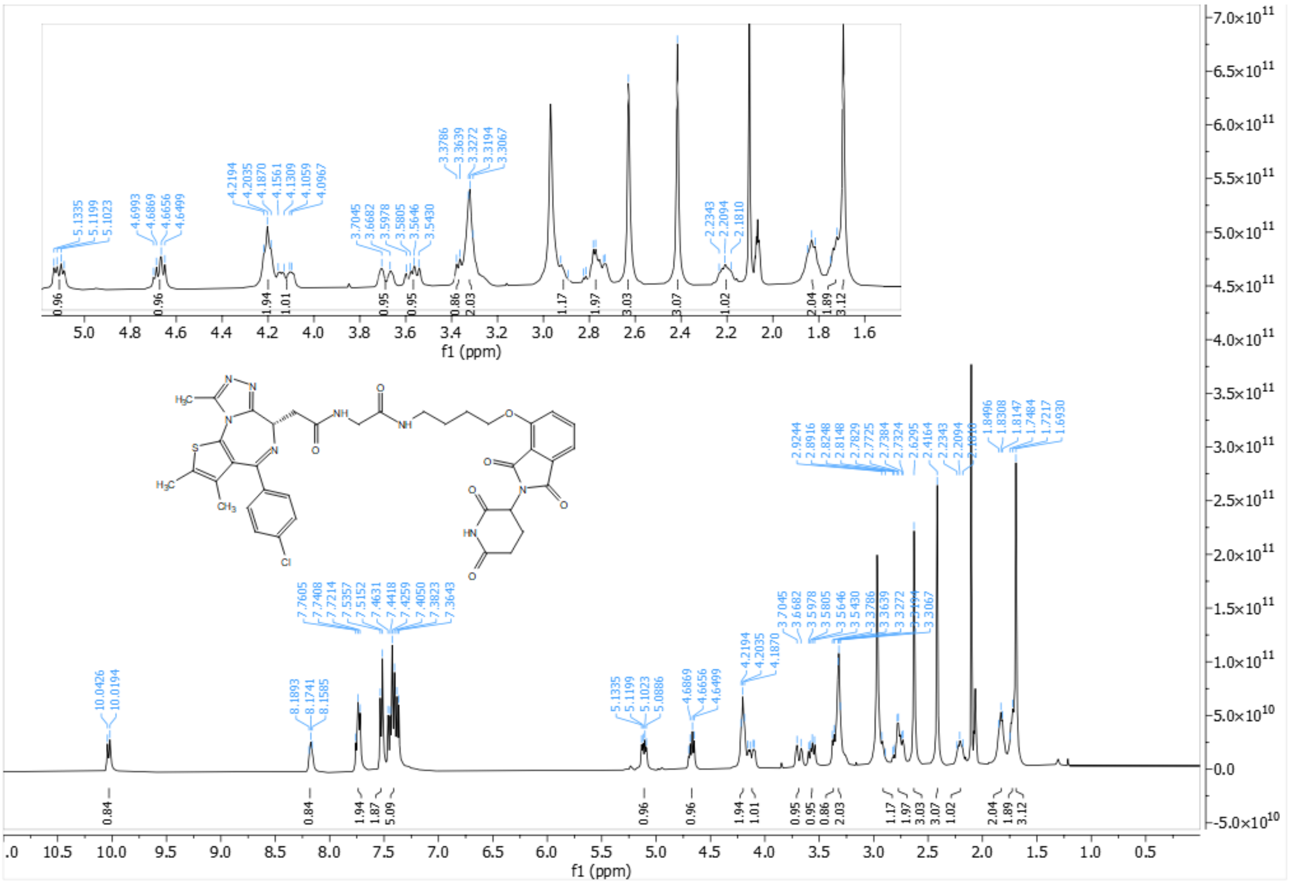

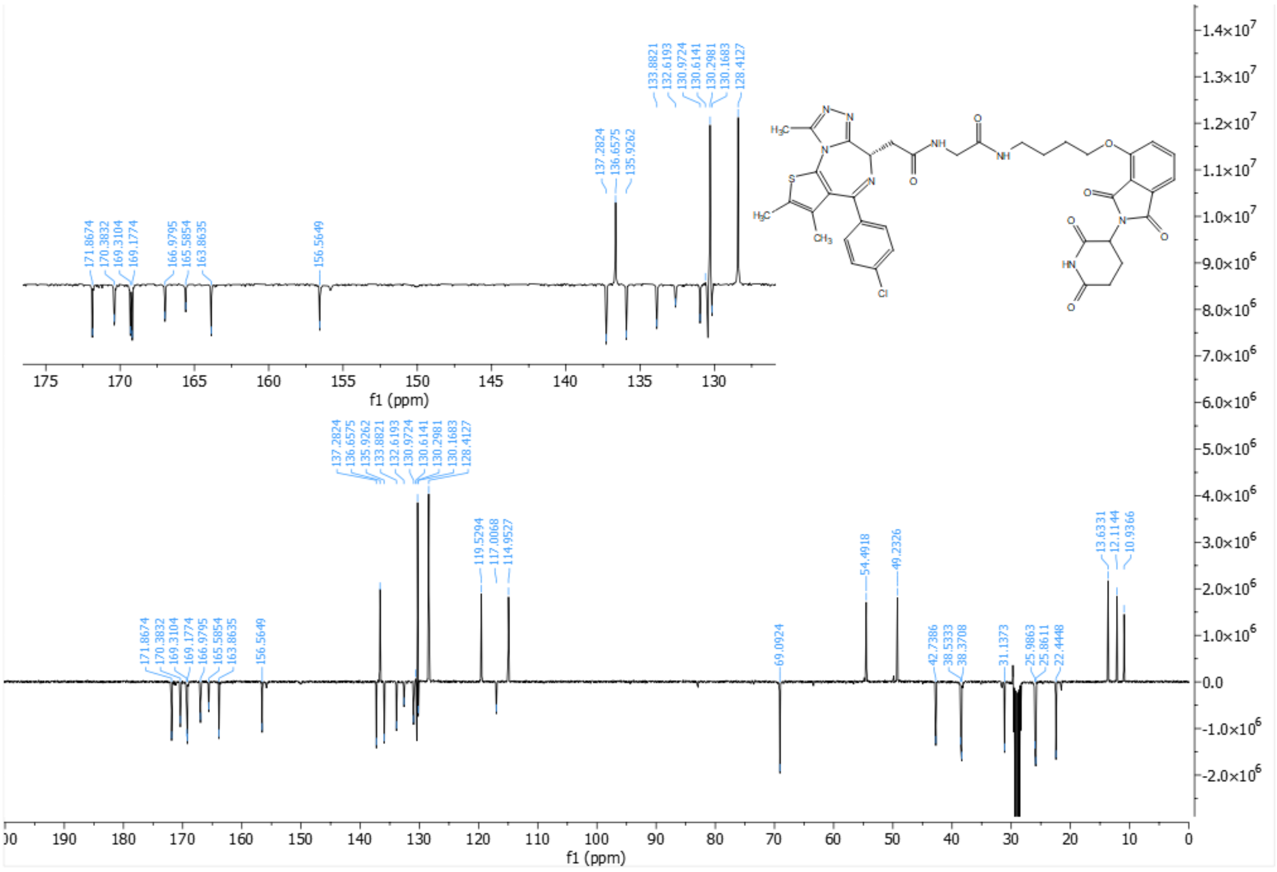

Figure S7. ^{1}H (400 MHz, $(CD_3)_2CO$, top) and ^{13}C (101 MHz, $(CD_3)_2CO$, bottom) NMR spectra of compound **28**.

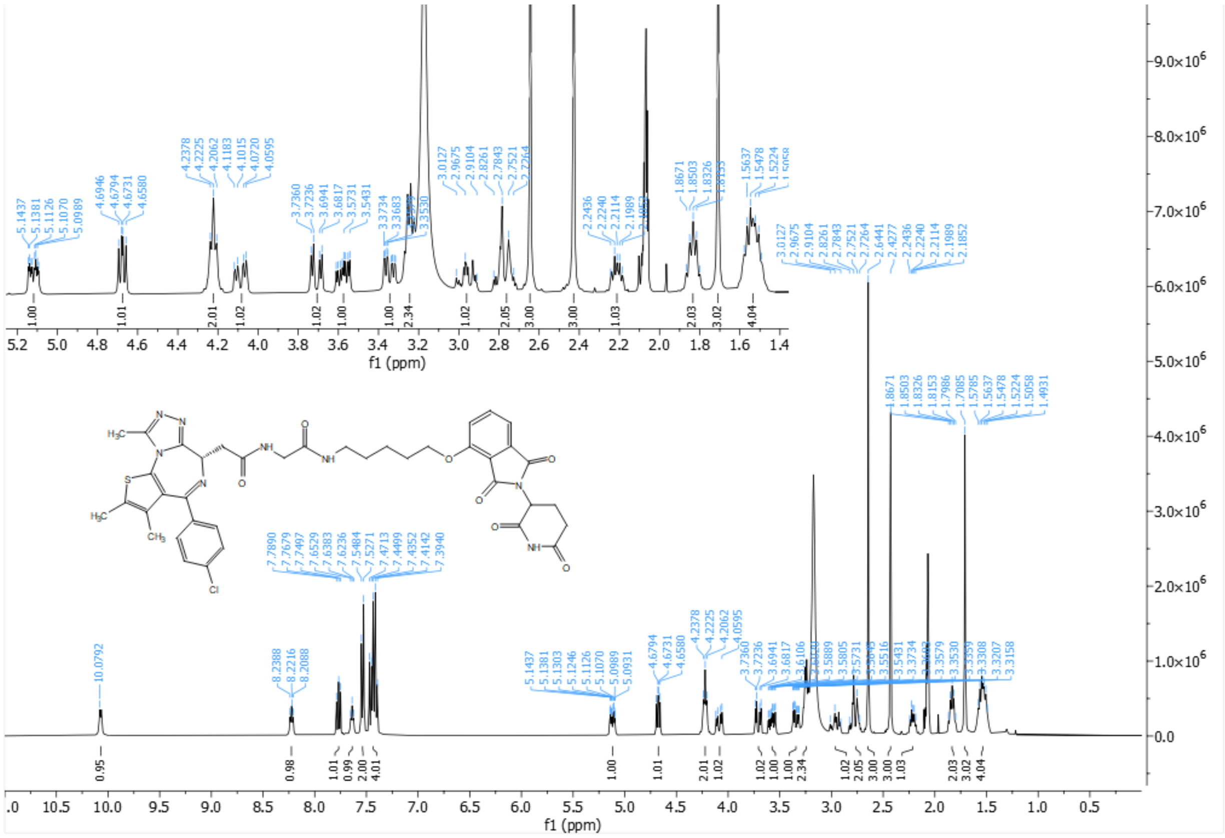

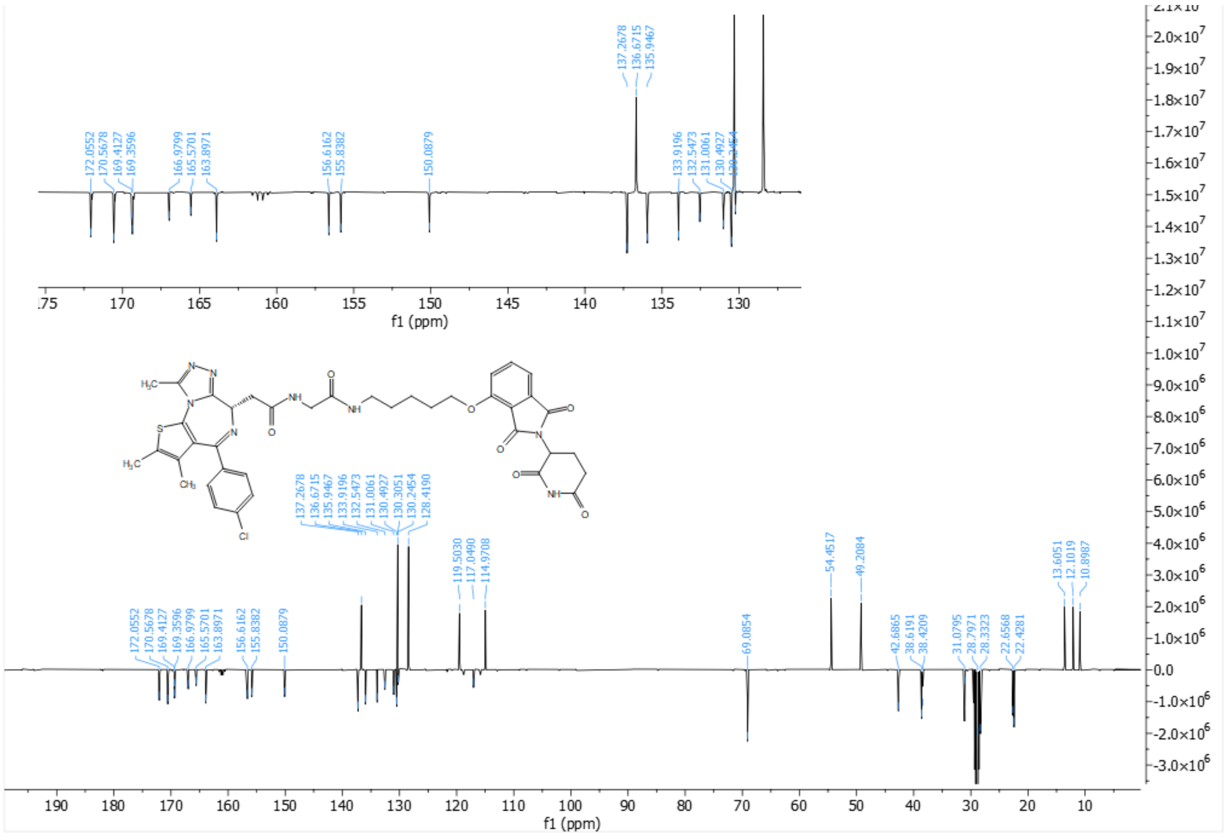

Figure S8. ^{1}H (400 MHz, $(CD_3)_2CO$, top) and ^{13}C (101 MHz, $(CD_3)_2CO$, bottom) NMR spectra of compound **29**.

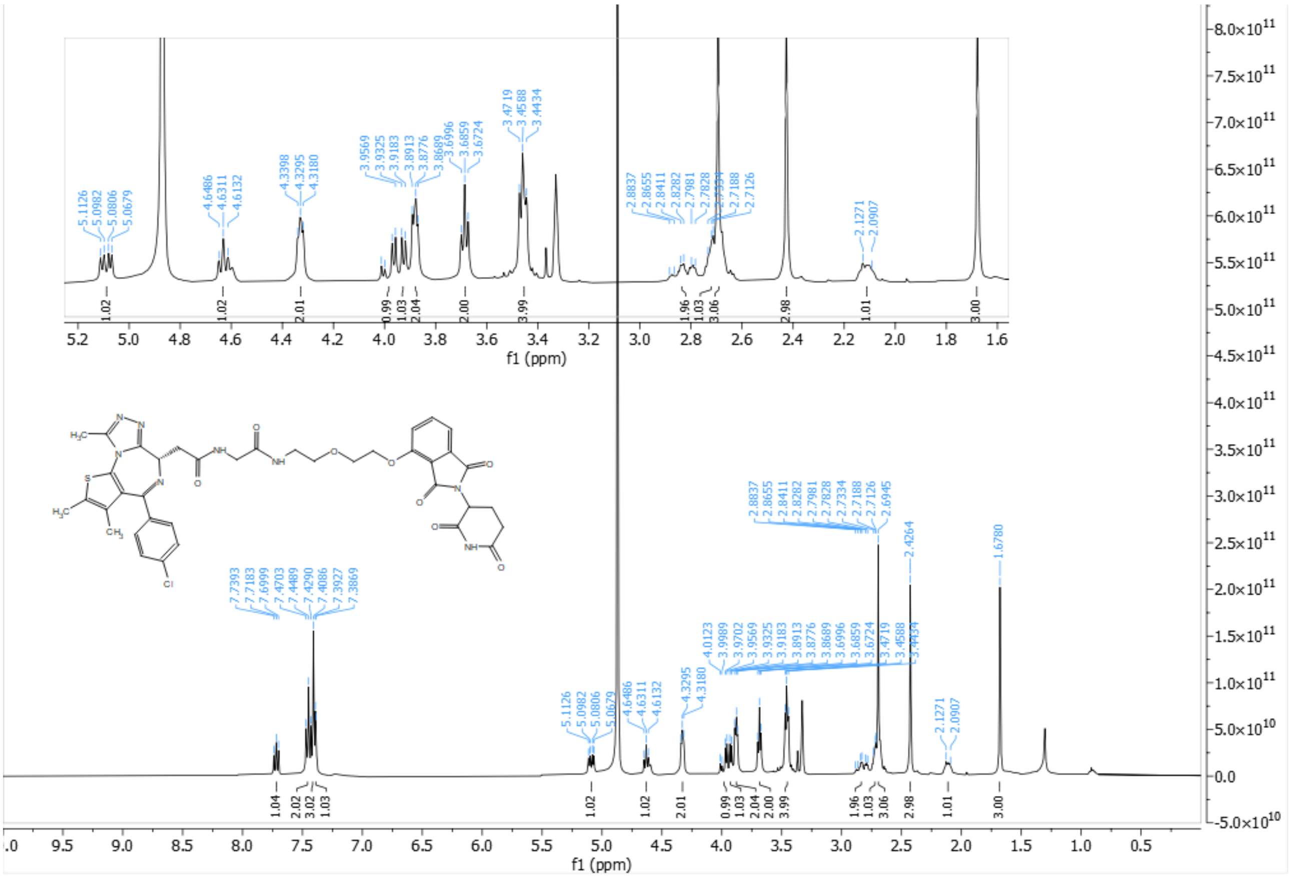

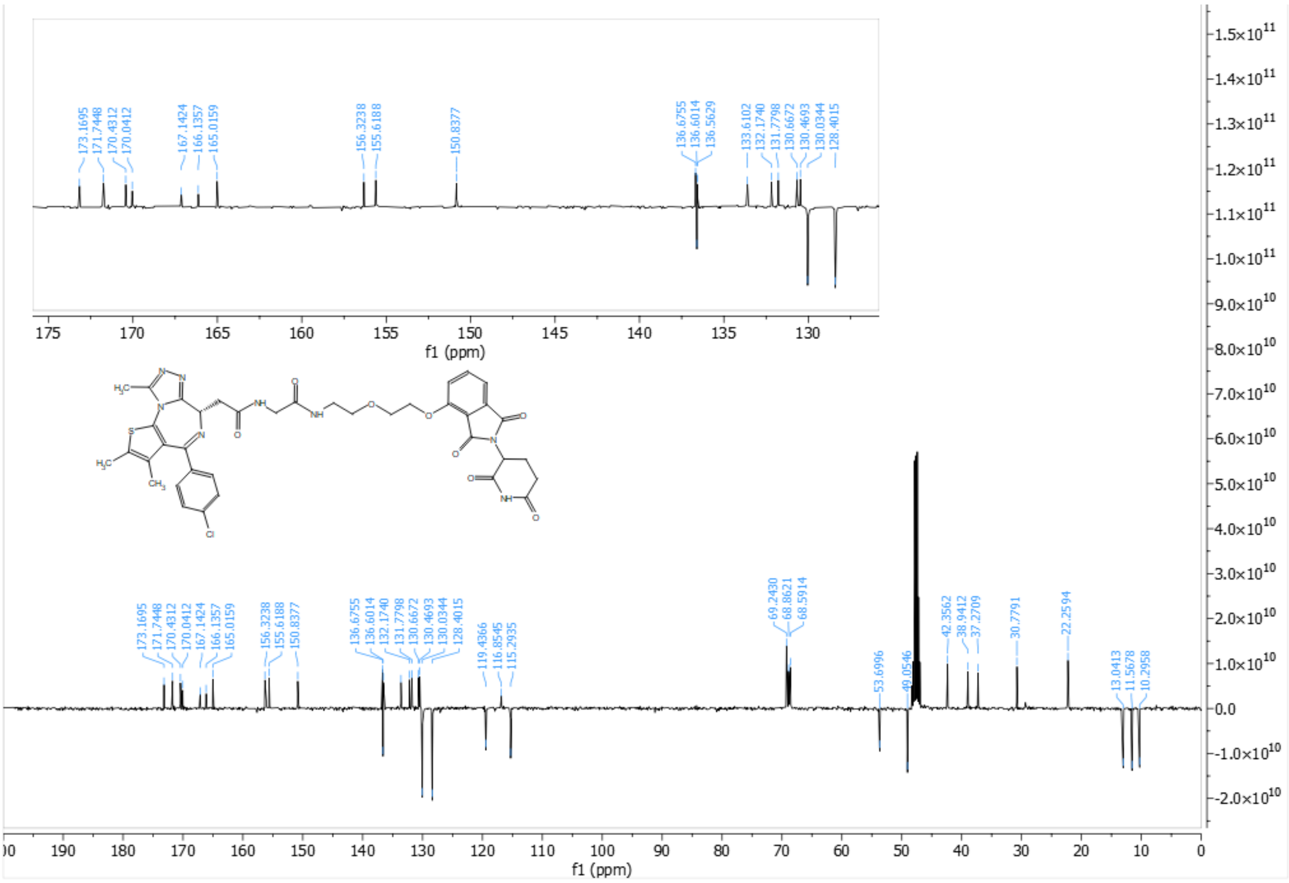

Figure S9. ^{1}H (400 MHz, CD_3OD, top) and ^{13}C (101 MHz, CD_3OD, bottom) NMR spectra of compound **30**.

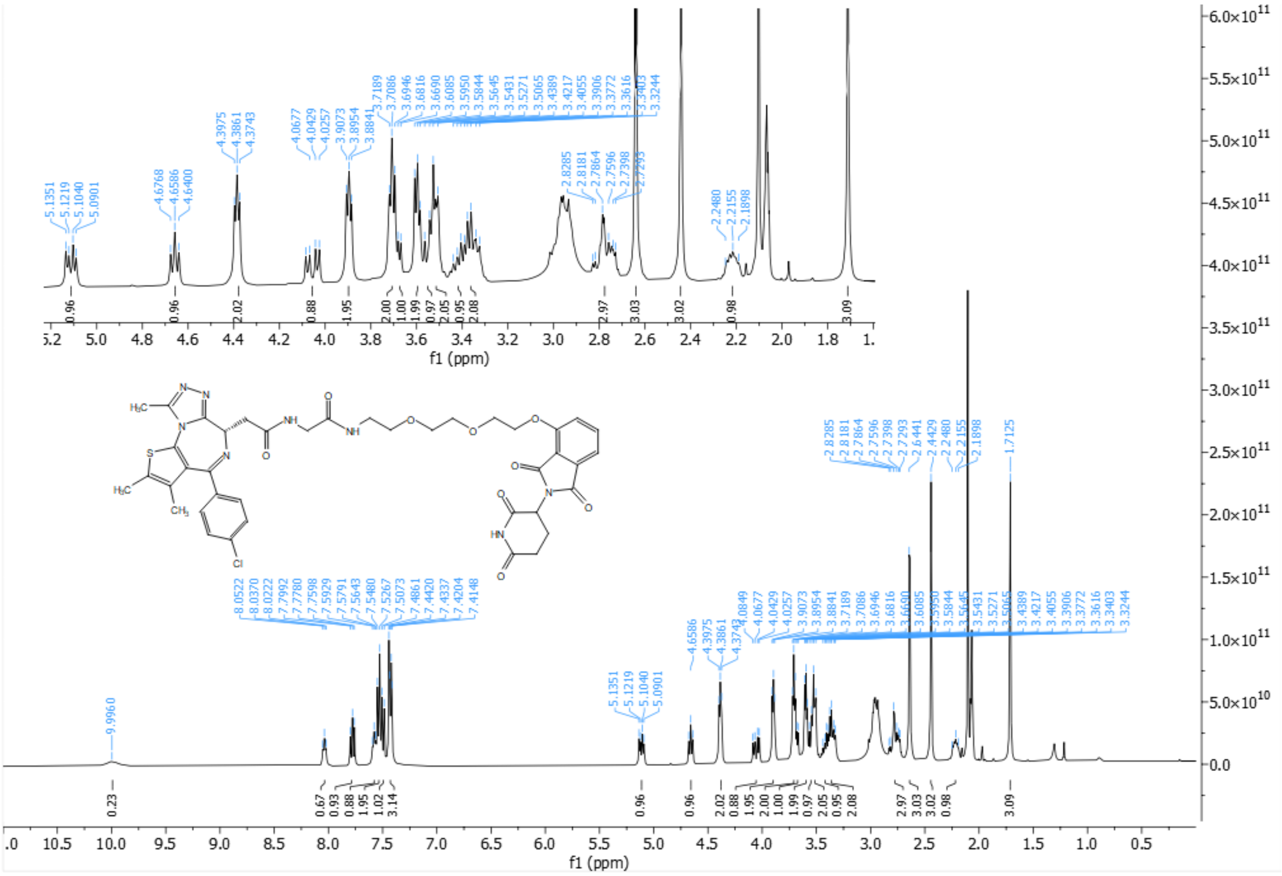

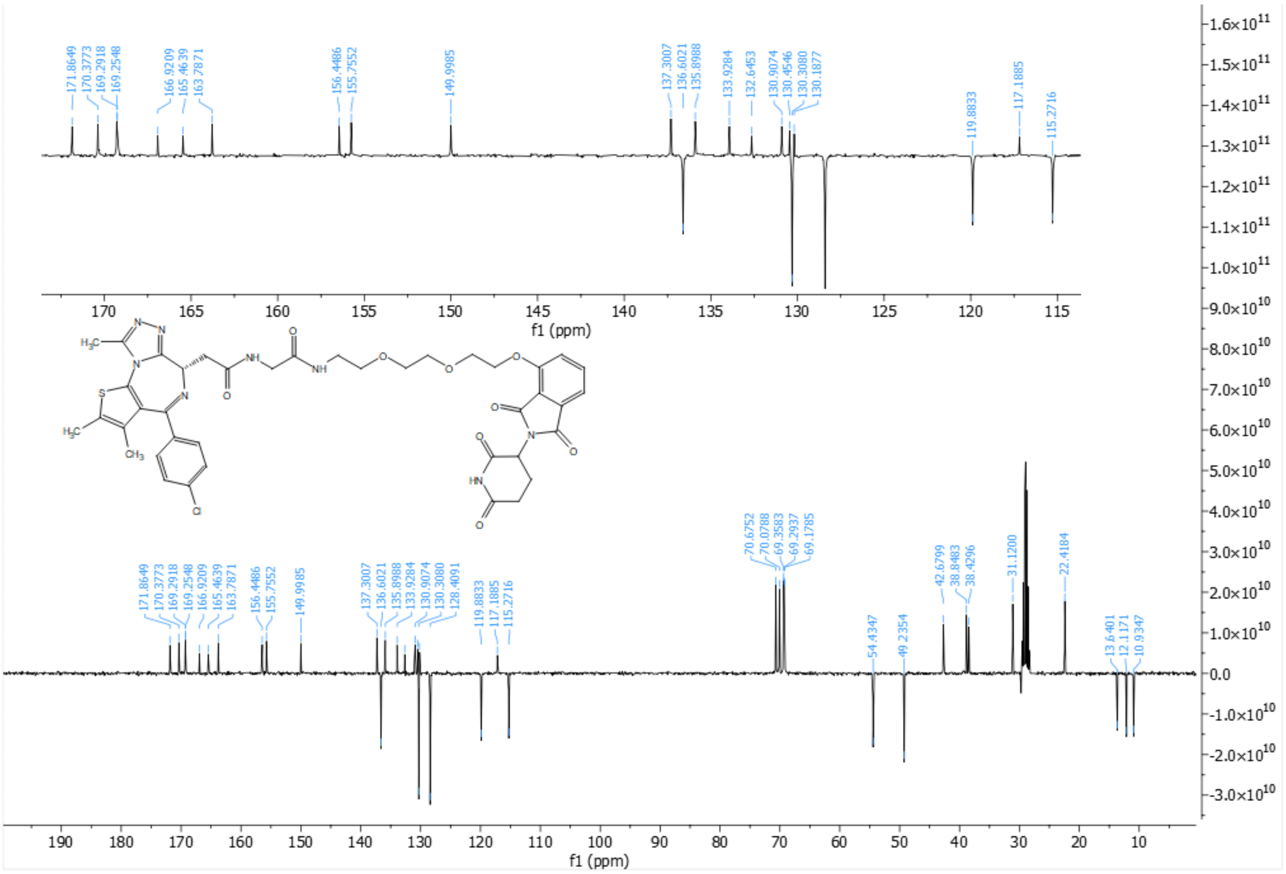

Figure S10. ^{1}H (400 MHz, $(CD_3)_2CO$, top) and ^{13}C (101 MHz, $(CD_3)_2CO$, bottom) NMR spectra of compound **31**.

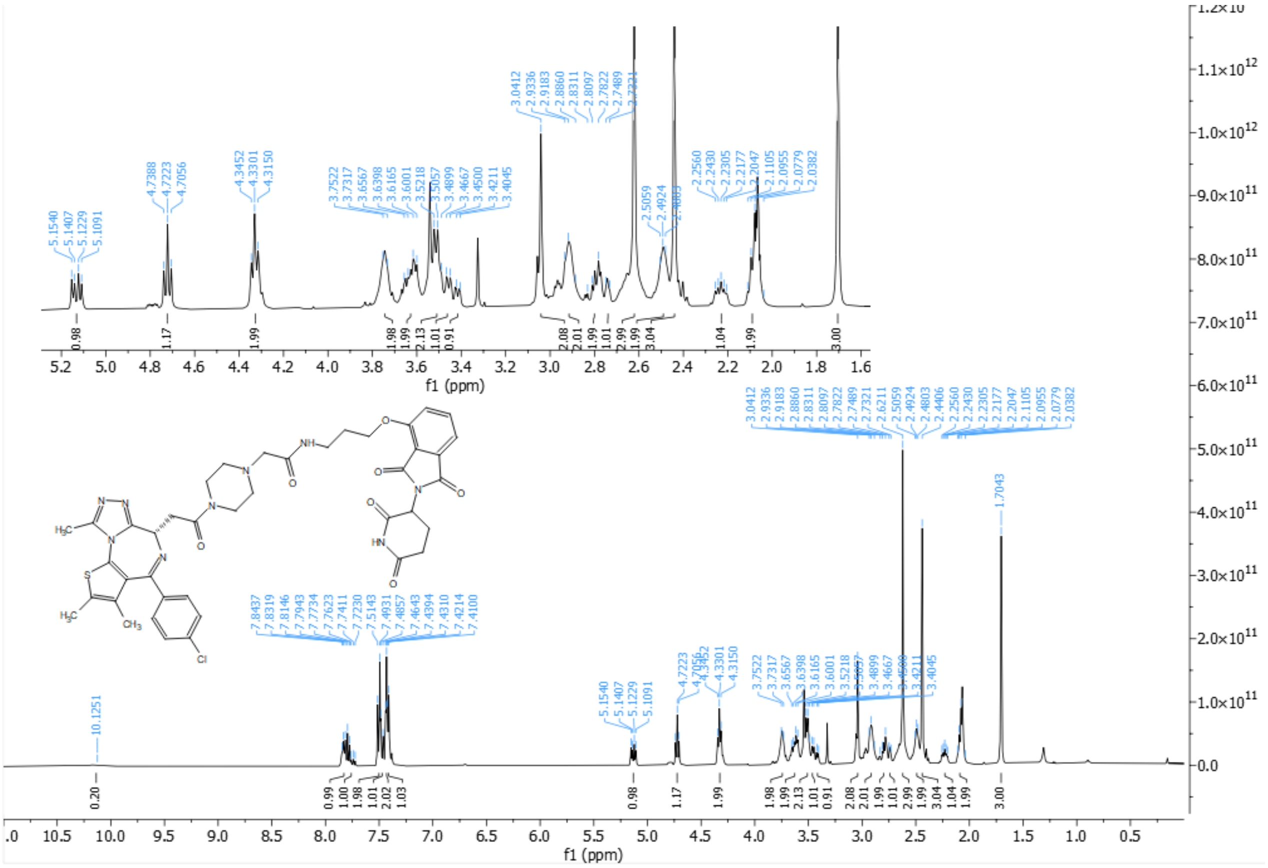

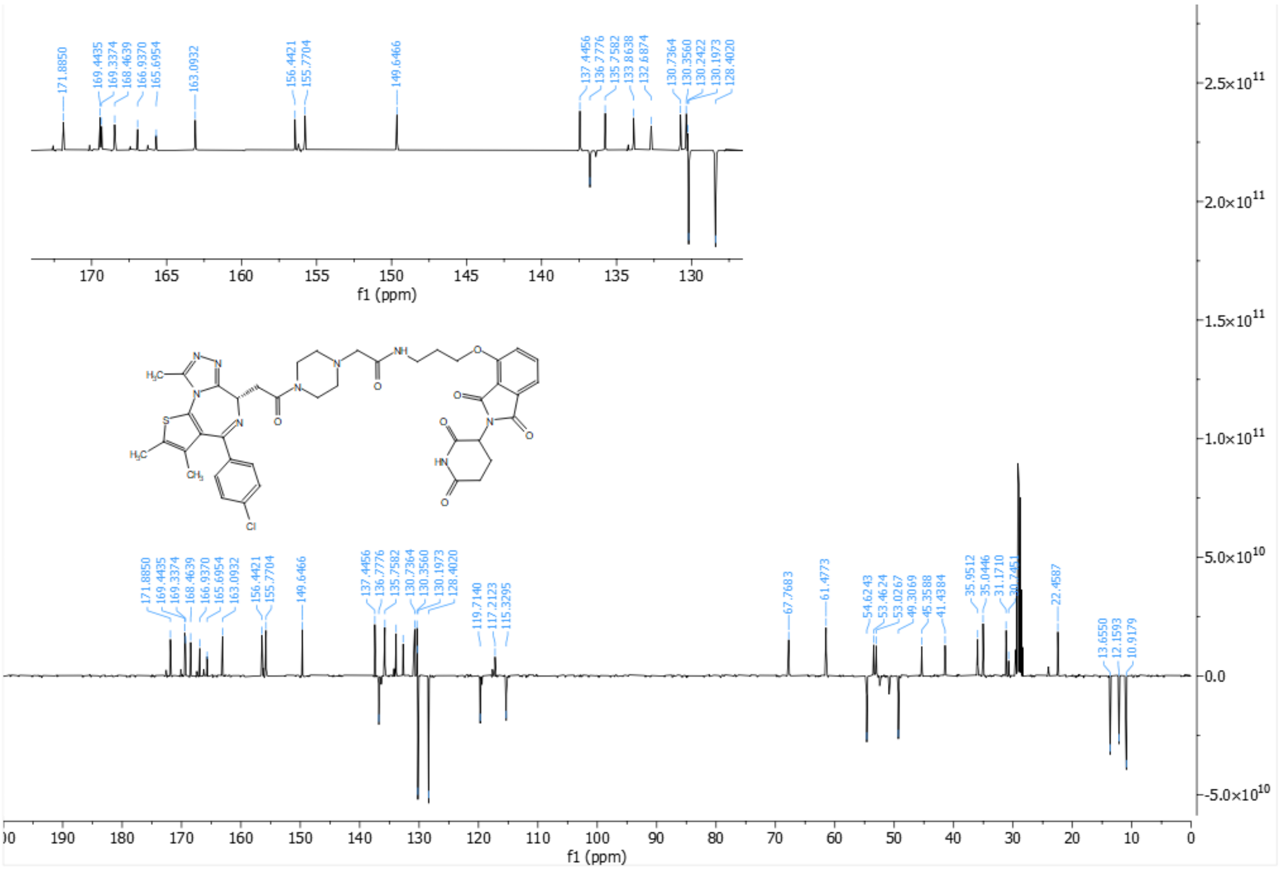

Figure S11. ^{1}H (400 MHz, $(CD_3)_2CO$, top) and ^{13}C (101 MHz, $(CD_3)_2CO$, bottom) NMR spectra of compound **32**.

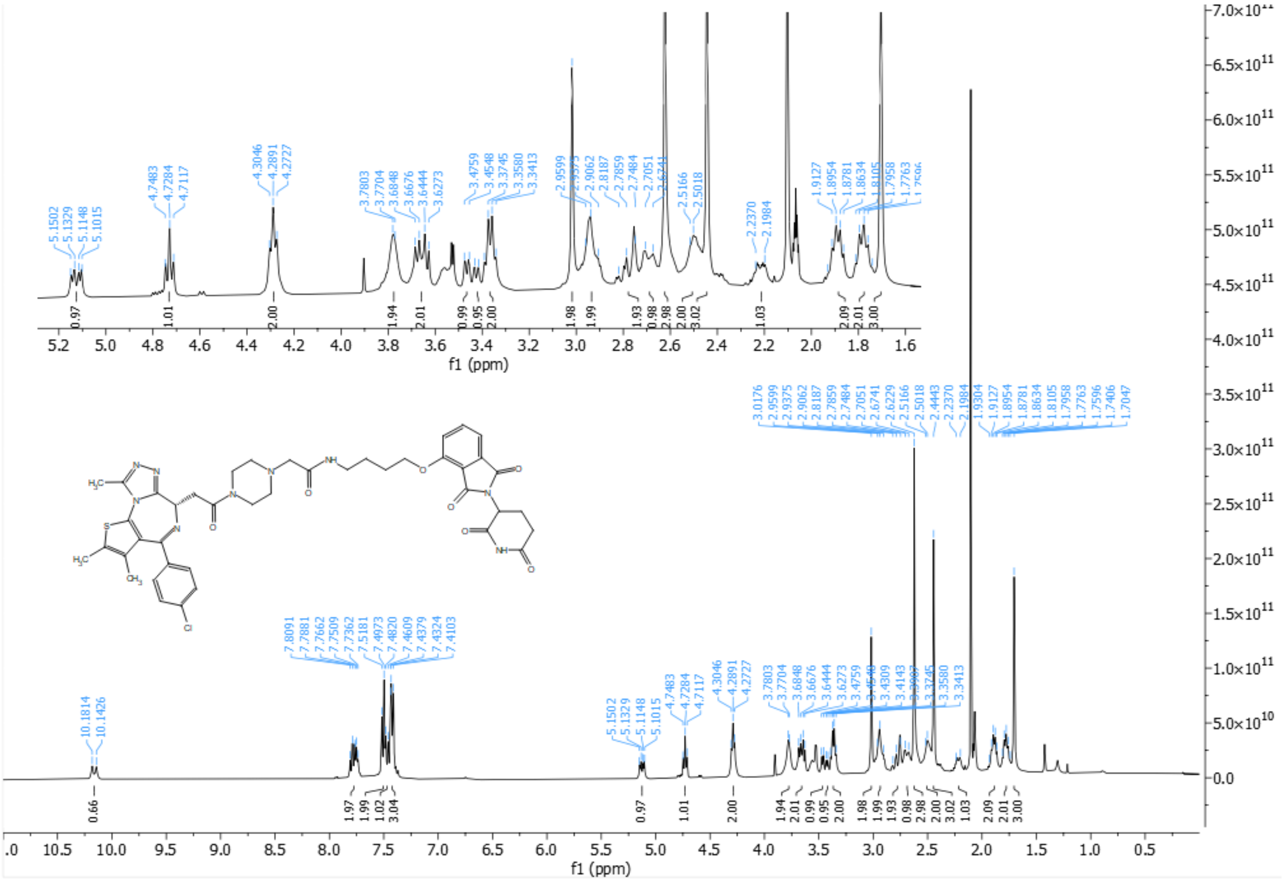

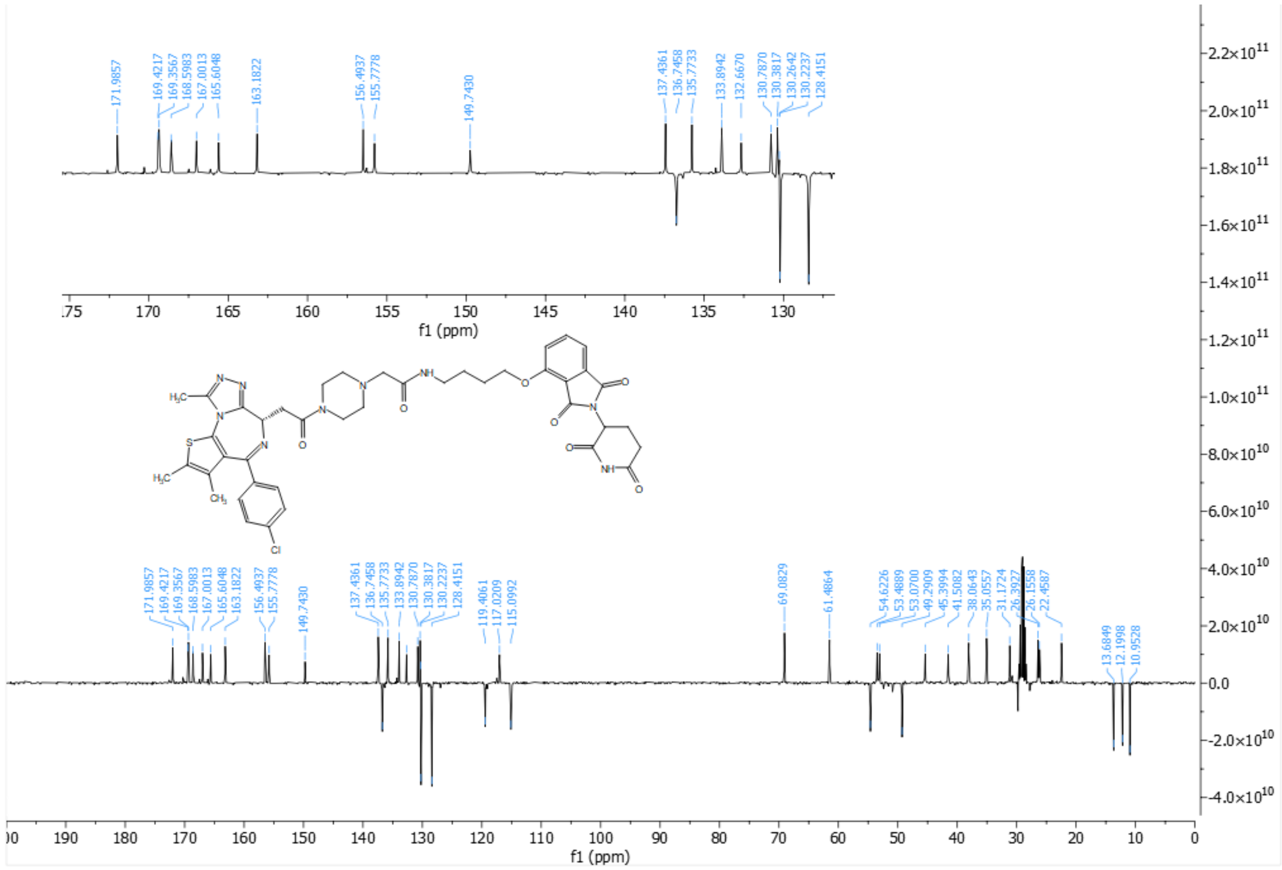

Figure S12. ^{1}H (400 MHz, $(CD_3)_2CO$, top) and ^{13}C (101 MHz, $(CD_3)_2CO$, bottom) NMR spectra of compound **33**.

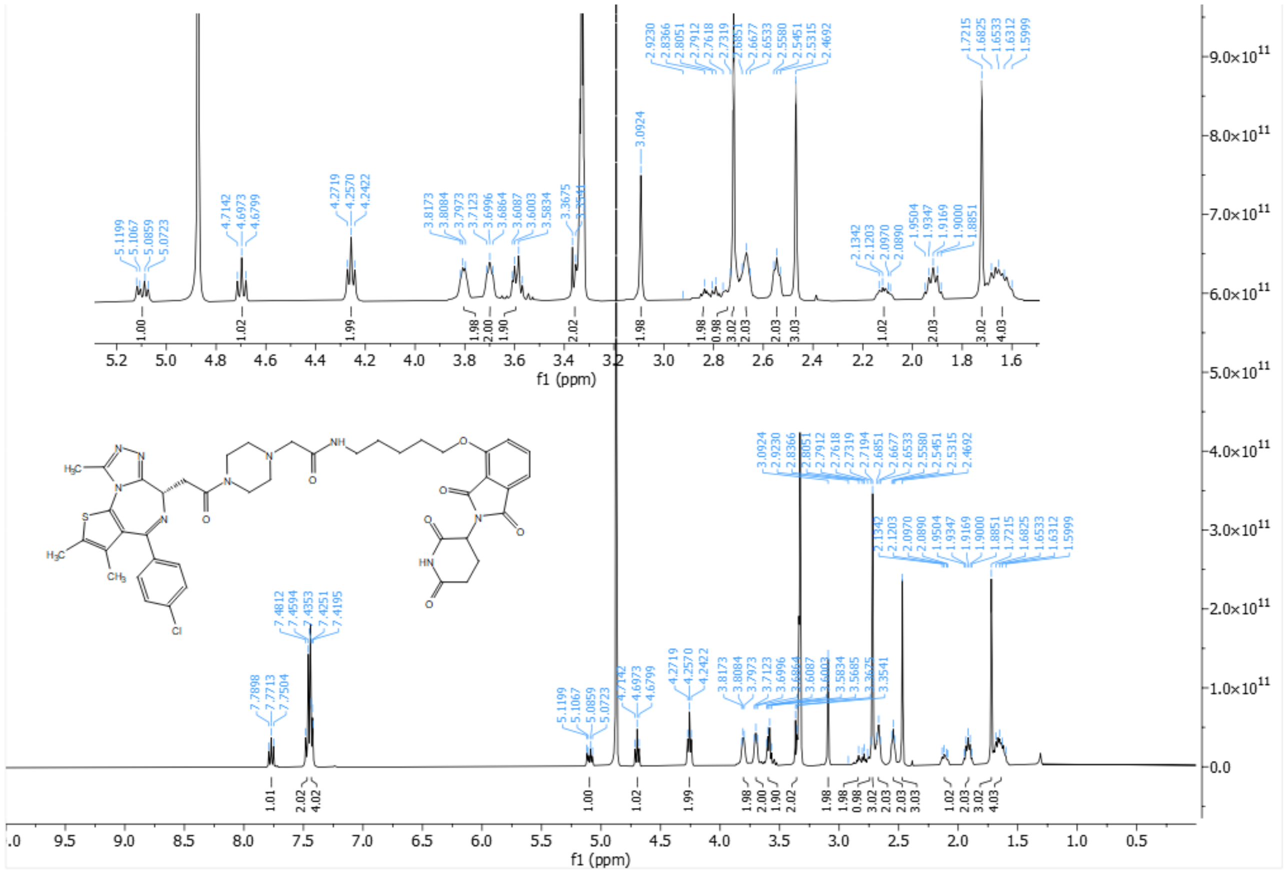

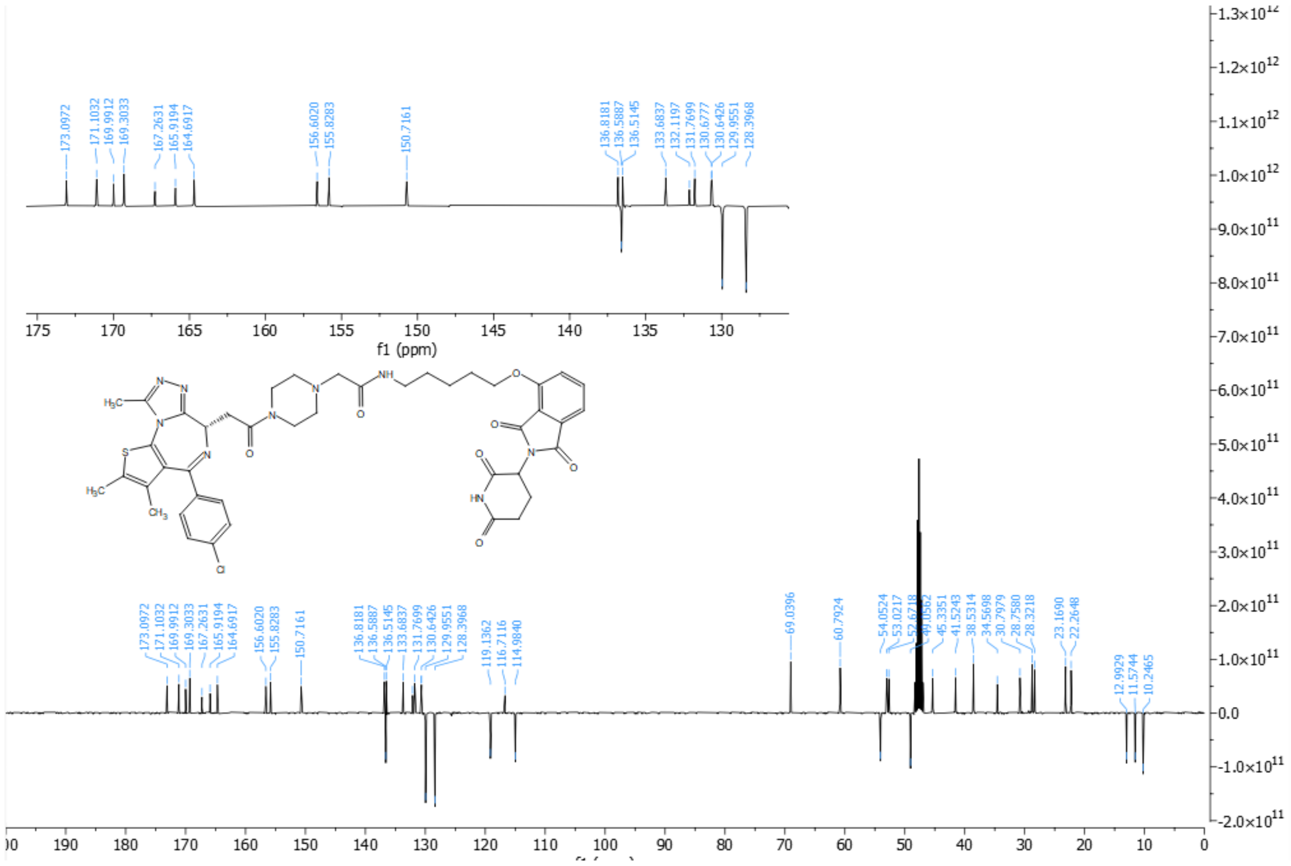

Figure S13. ^{1}H (400 MHz, CD_3OD, top) and ^{13}C (101 MHz, CD_3OD, bottom) NMR spectra of compound **34**.

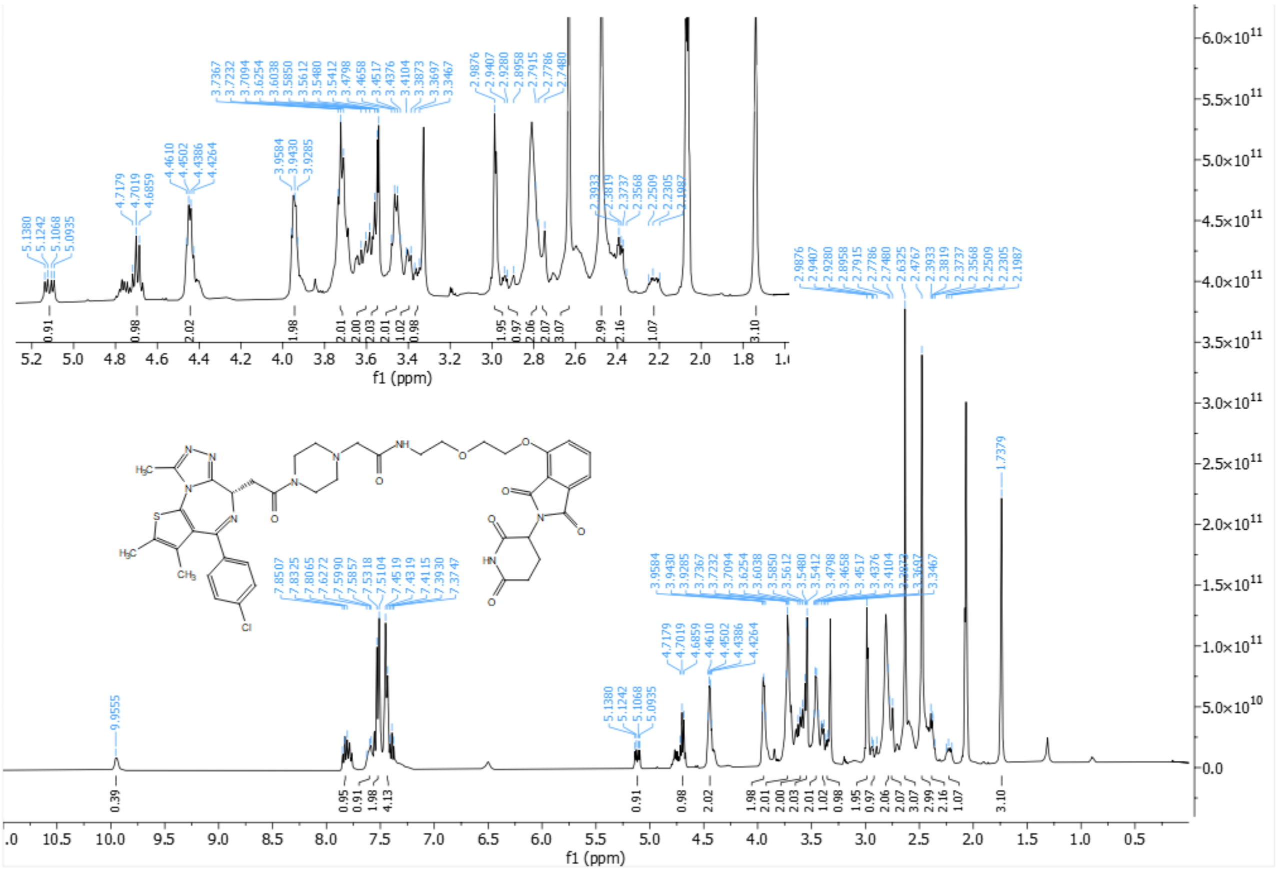

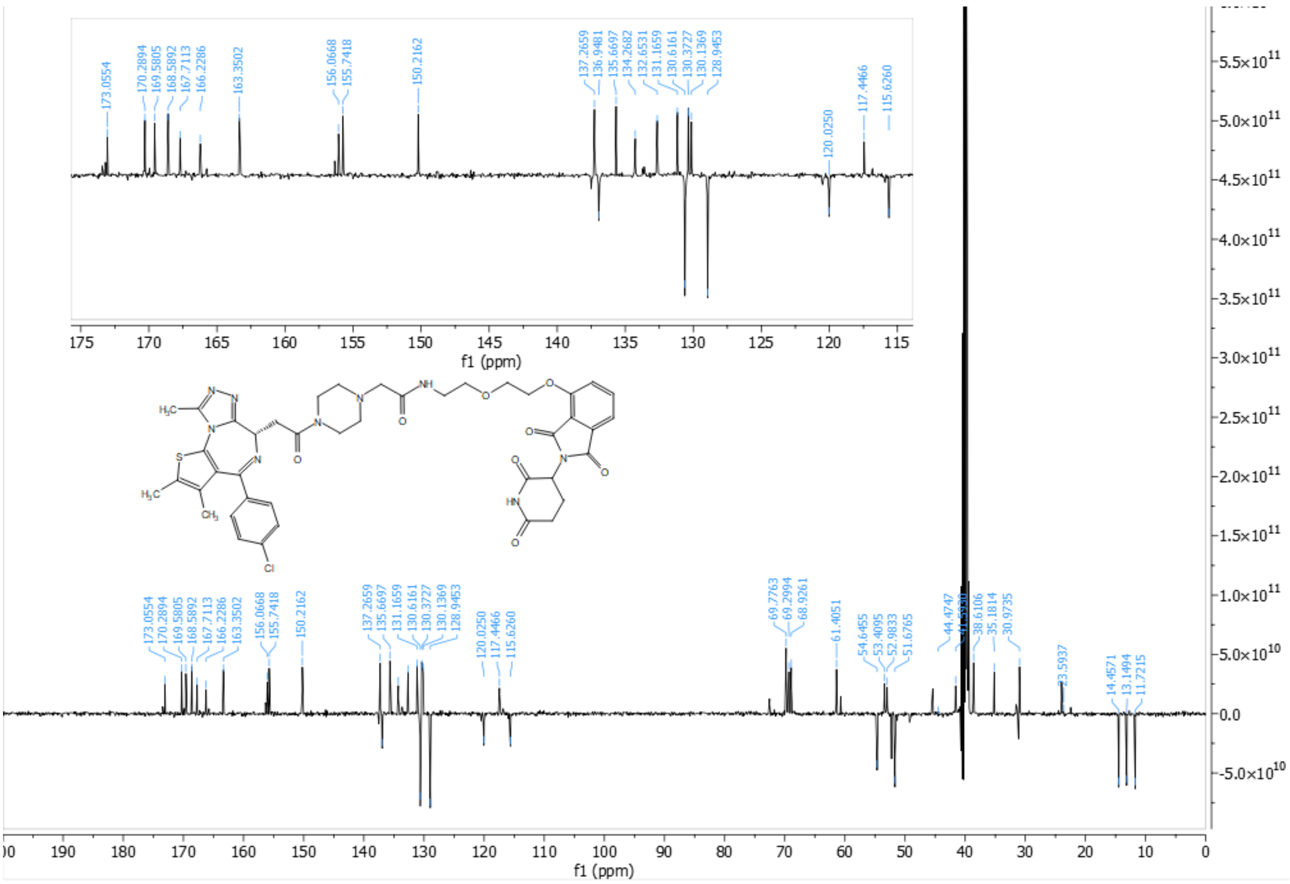

Figure S14. ^{1}H (400 MHz, $(CD_3)_2CO$, top) and ^{13}C (101 MHz, $(CD_3)_2CO$, bottom) NMR spectra of compound **35**.

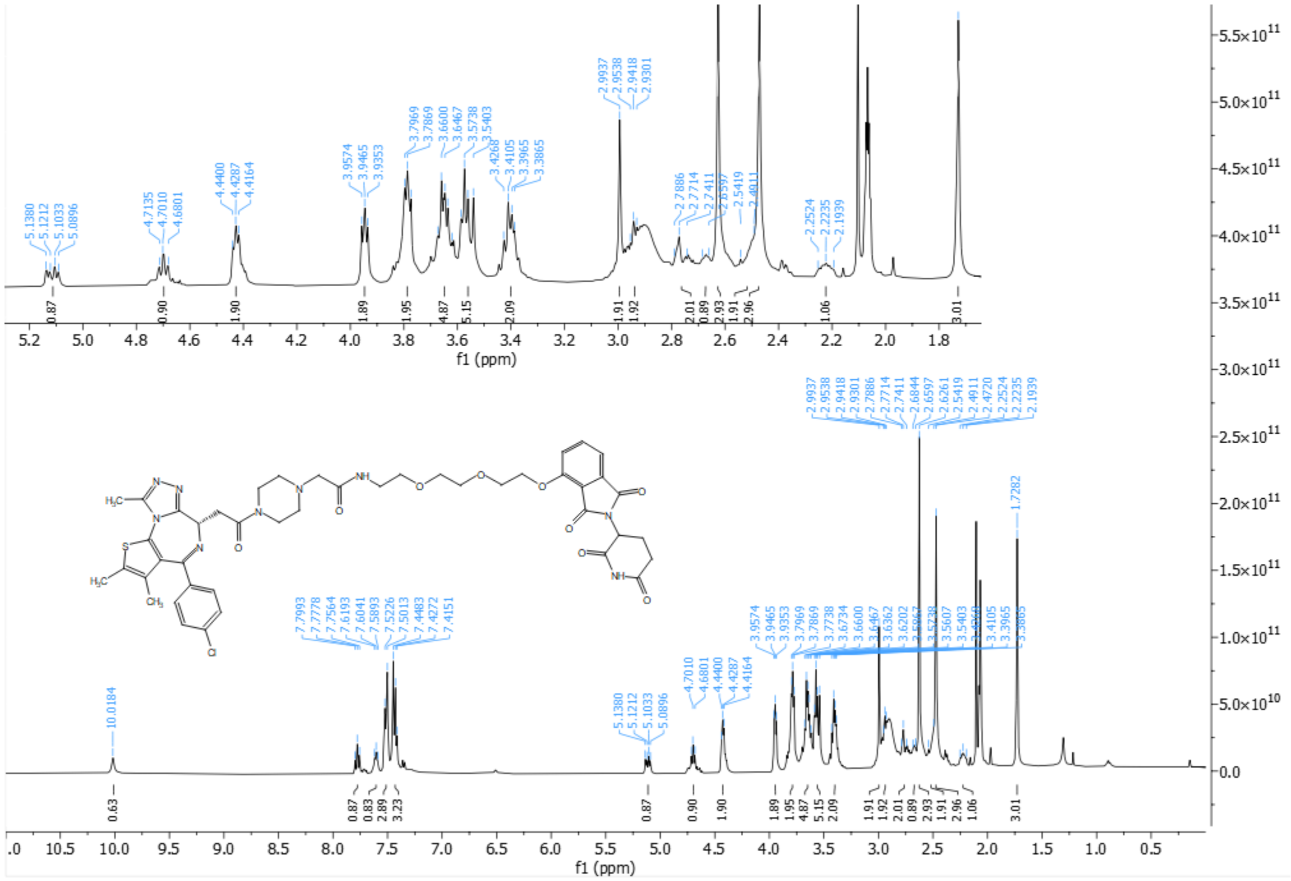

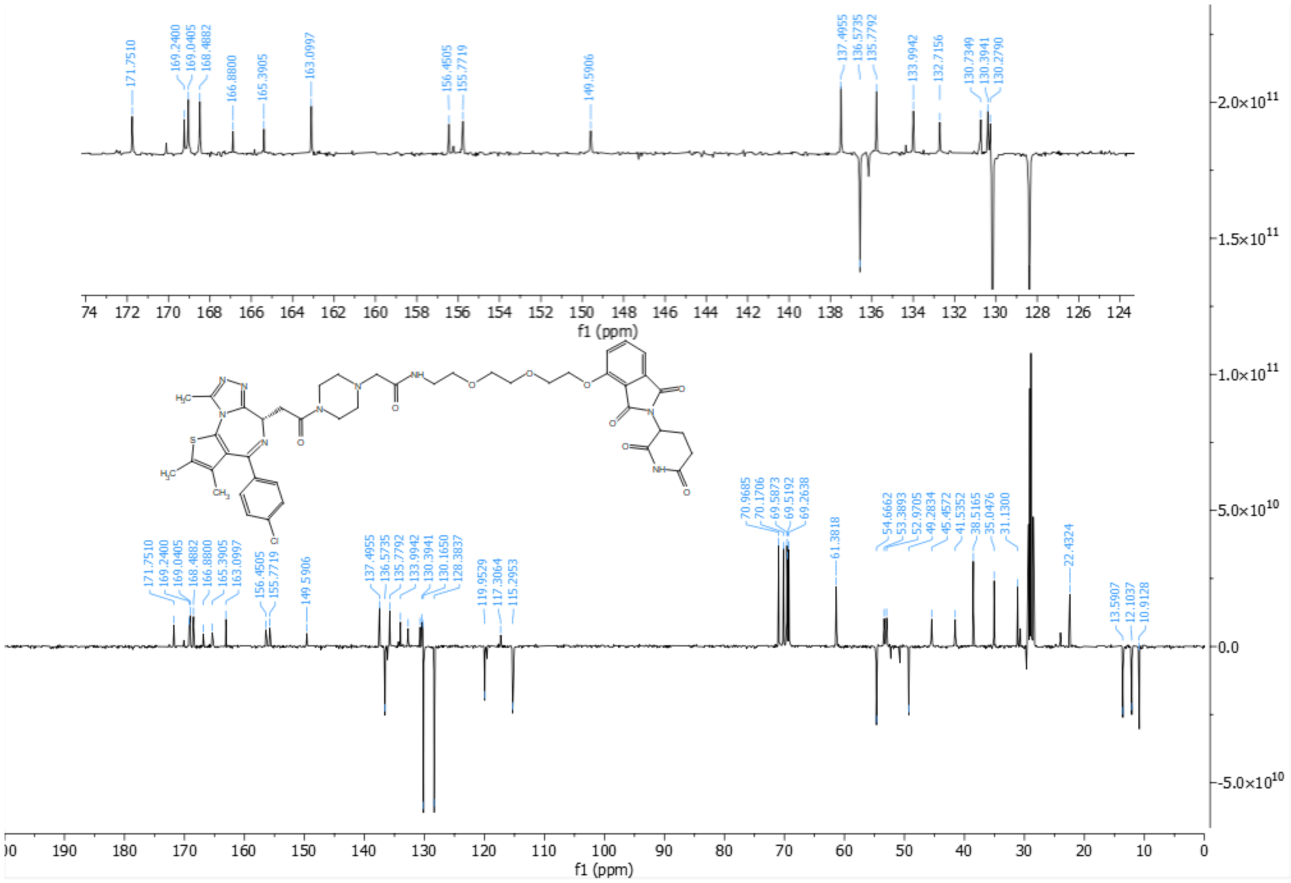

Figure S15. ^{1}H (400 MHz, $(CD_3)_2CO$, top) and ^{13}C (101 MHz, $(CD_3)_2CO$, bottom) NMR spectra of compound **36**.

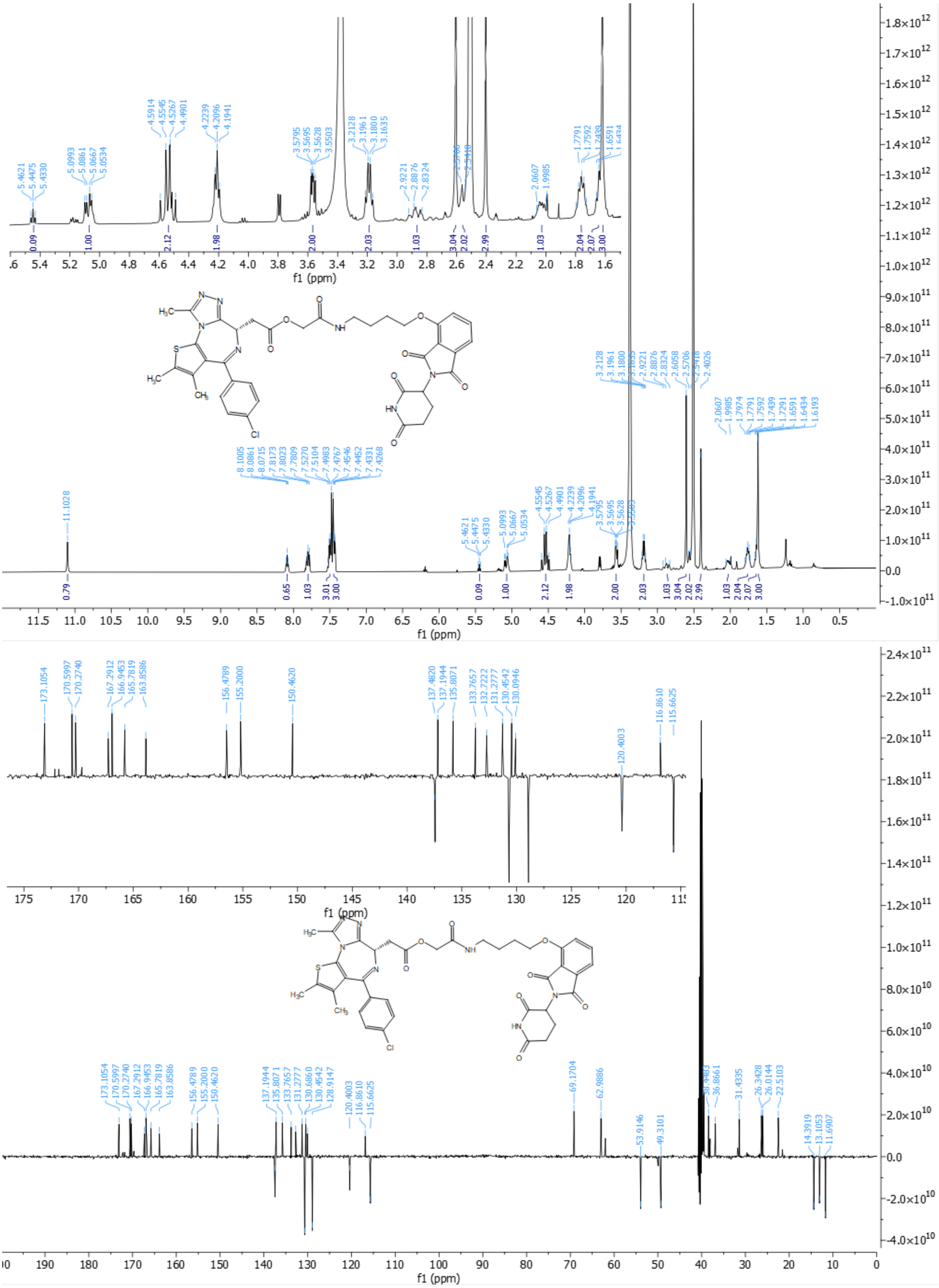

Figure S16. ^{1}H (400 MHz, DMSO-d_6, top) and ^{13}C (101 MHz, DMSO-d_6, bottom) NMR spectra of compound **37**.

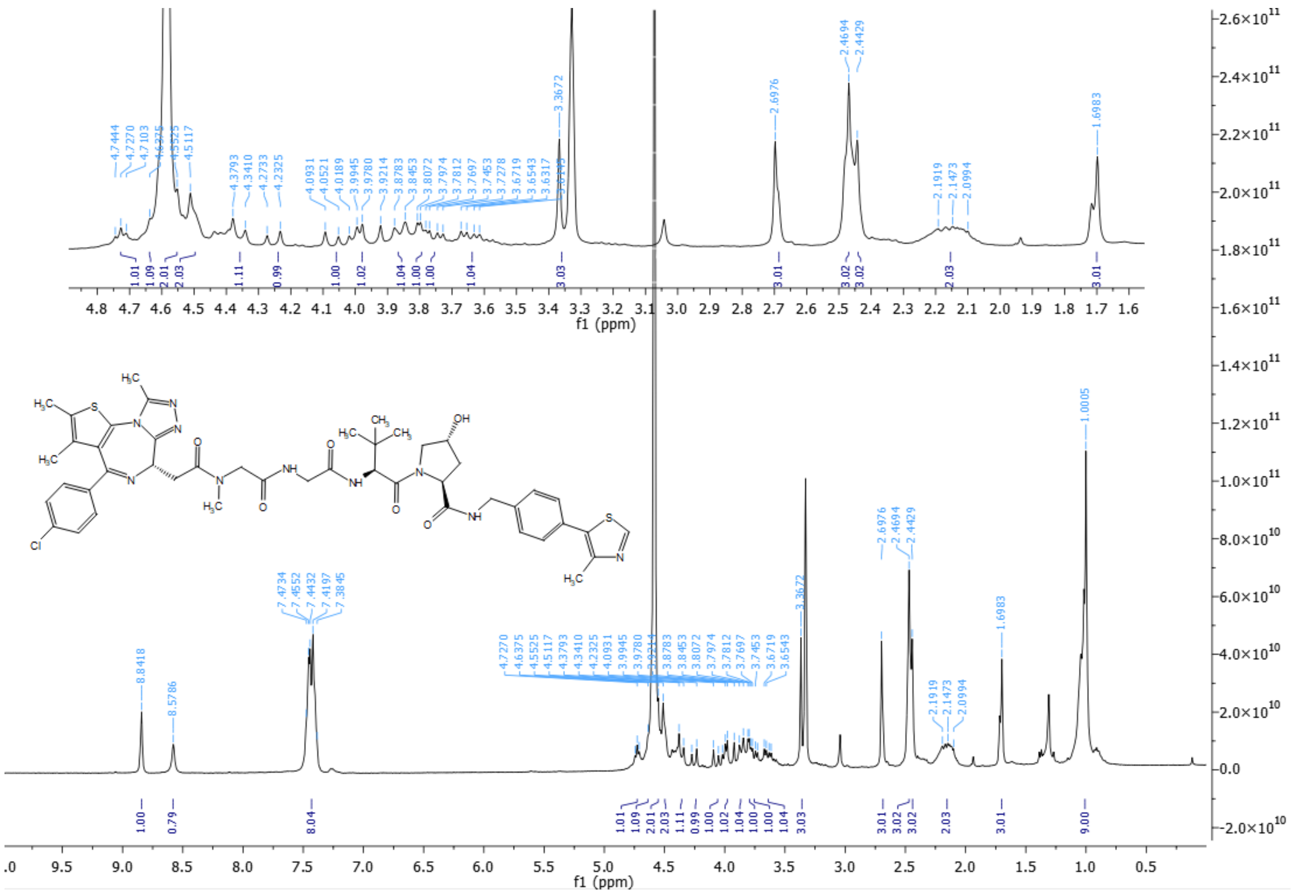

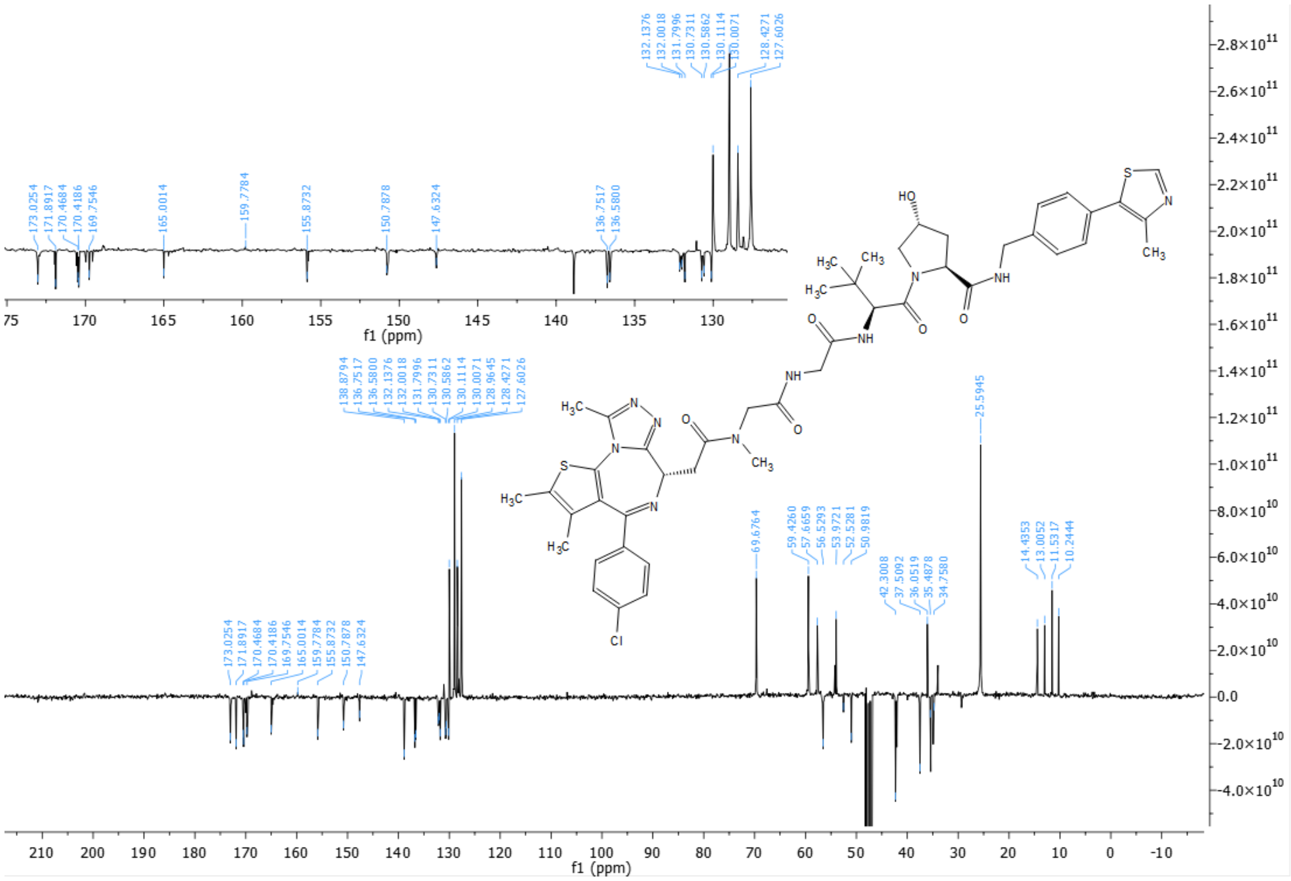

Figure S17. ^{1}H (400 MHz, CD_3OD, top) and ^{13}C (101 MHz, CD_3OD, bottom) NMR spectra of compound **45**.

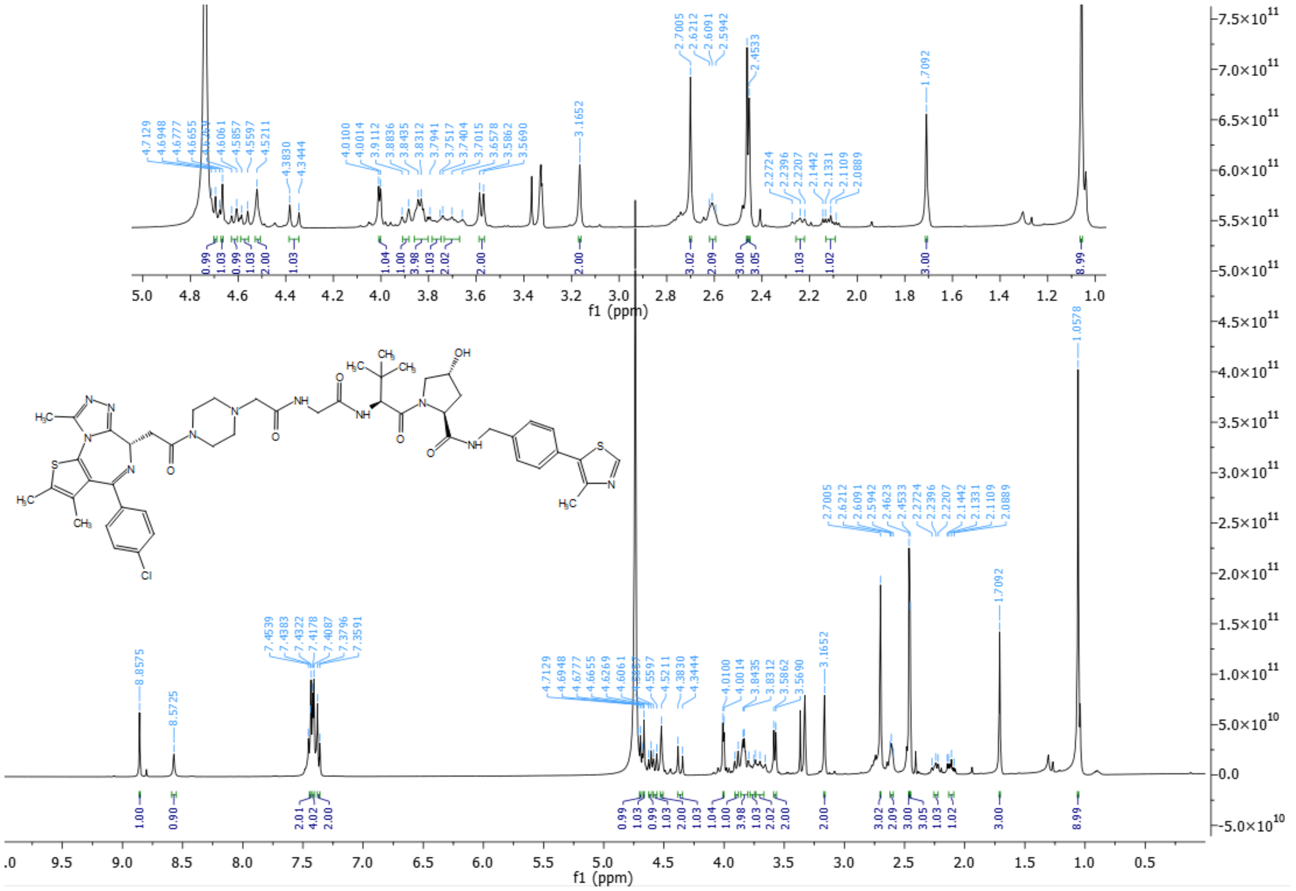

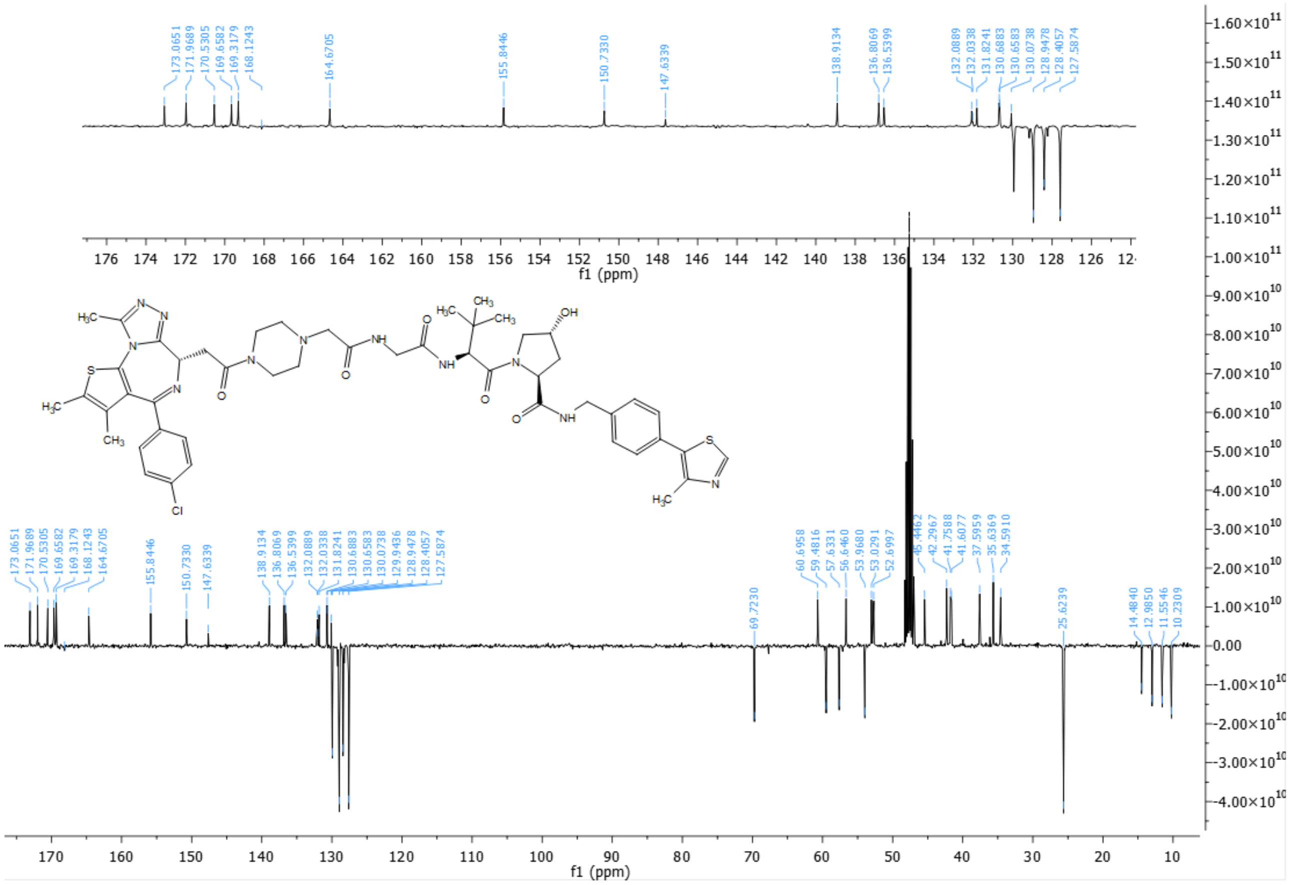

Figure S18. ^{1}H (400 MHz, CD_3OD, top) and ^{13}C (101 MHz, CD_3OD, bottom) NMR spectra of compound **46**.

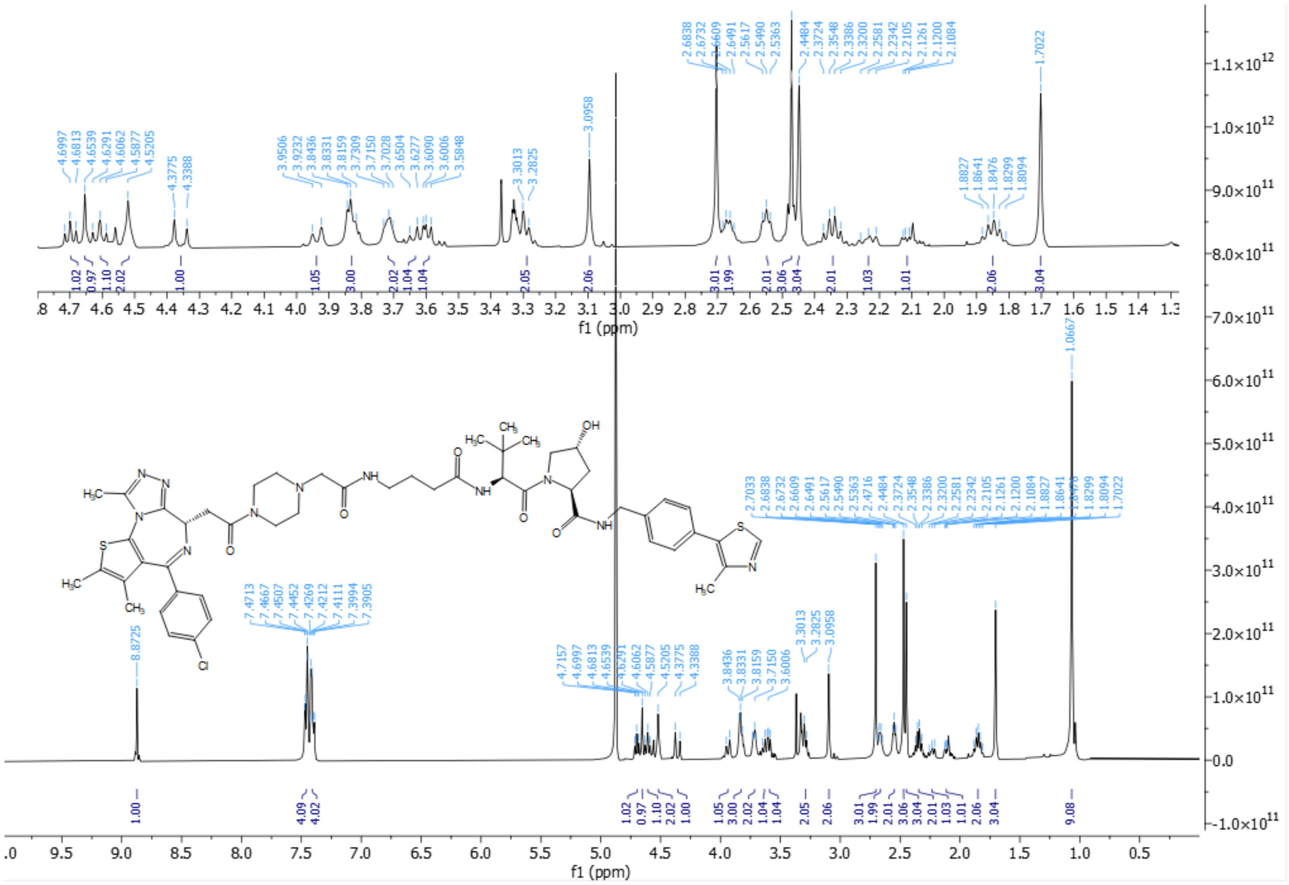

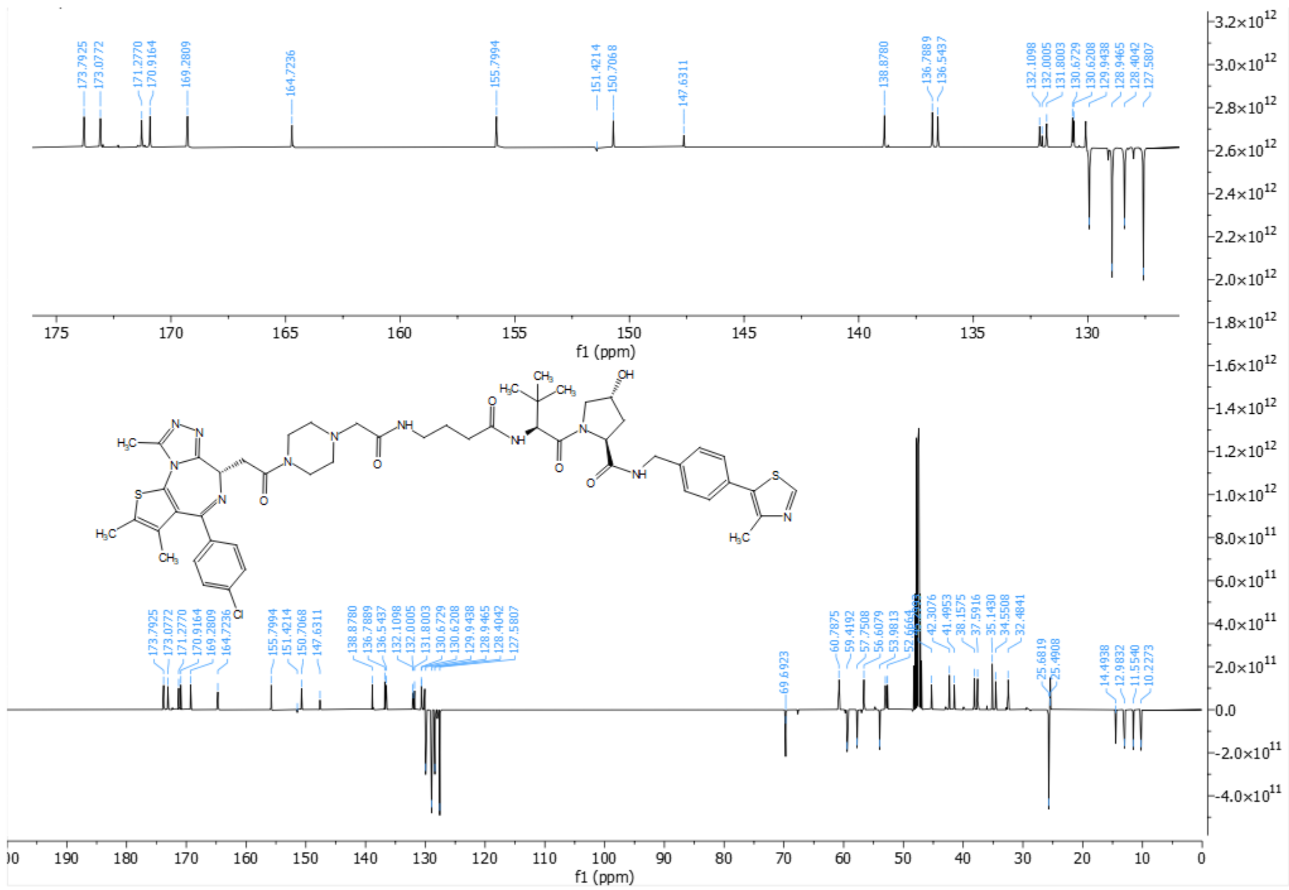

Figure S19. ^{1}H (400 MHz, CD_3OD, top) and ^{13}C (101 MHz, CD_3OD, bottom) NMR spectra of compound **47**.

LC-UV methods for determination of purity of selected active compounds, aqueous solubility, and metabolic stability.

Instrumentation: Shimadzu HPLC system LC-10AD series

Column: Kinetex C18 (150 × 4.6 mm, 5 µm d_p) (Phenomenex)

Mobile Phase A: 0.2% formic acid in water

B: 0.2% formic acid in acetonitrile

Analysis mode: Gradient of concentration

Time (min)	% B
0.00	20
13.50	80
14.50	80
15.00	20
20.00	20

Detection: λ = 254 nm

Flow rate: 1 mL/min

Injected volume: 20 µL

HPLC chromatograms

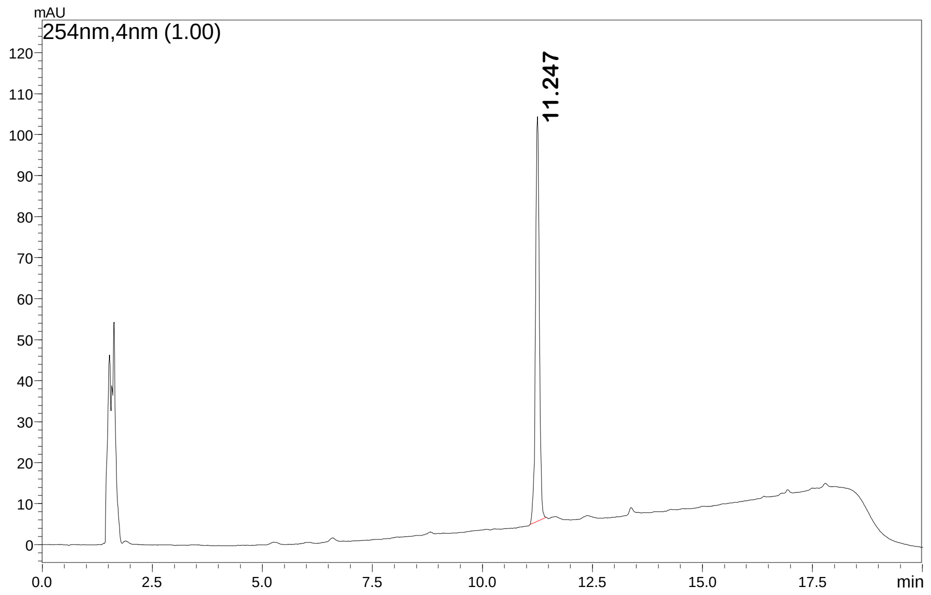

Figure S20. HPLC chromatogram of compound **27**; purity >99%.

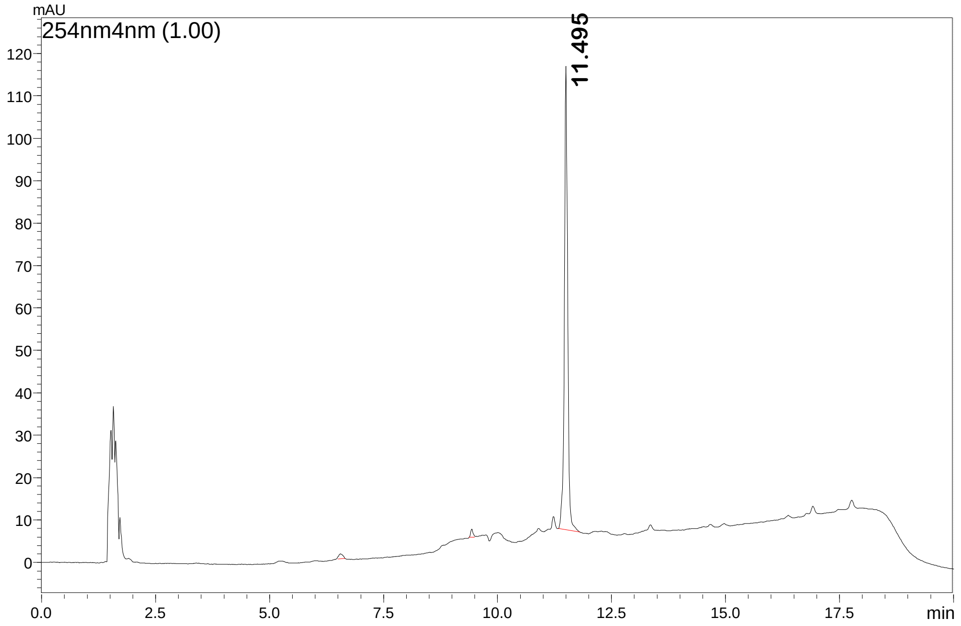

Figure S21. HPLC chromatogram of compound **28**; purity 95.6%.

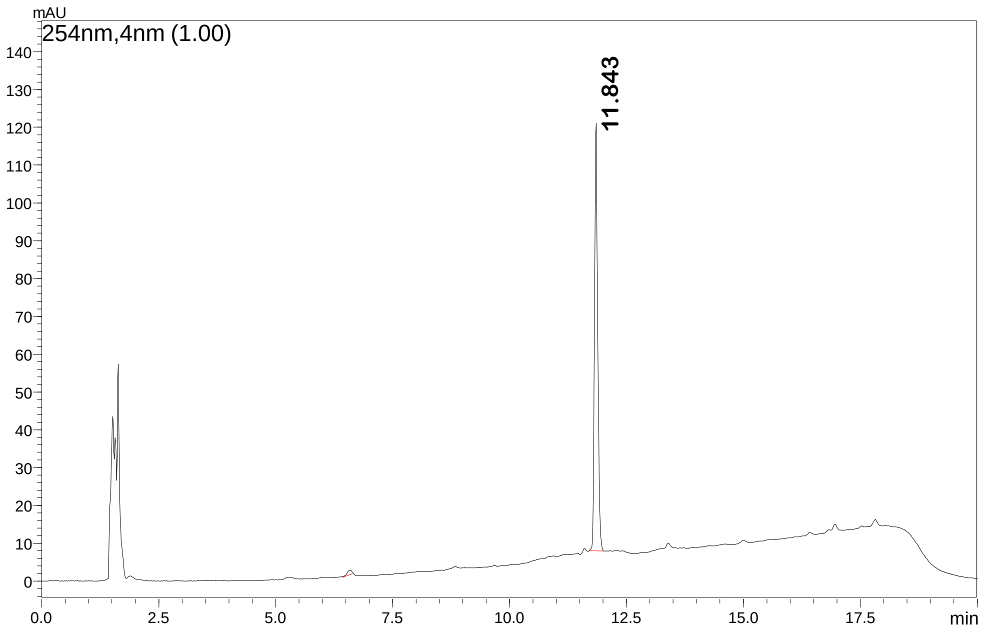

Figure S22. HPLC chromatogram of compound **29**; purity 97.8%.

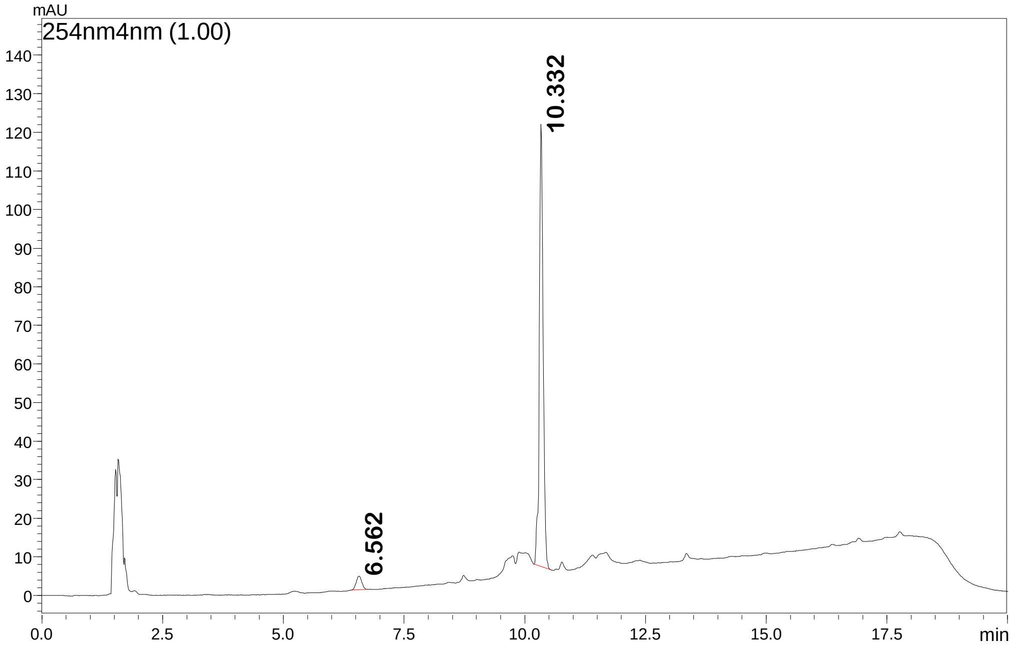

Figure S23. HPLC chromatogram of compound **34**; purity 95.7%.

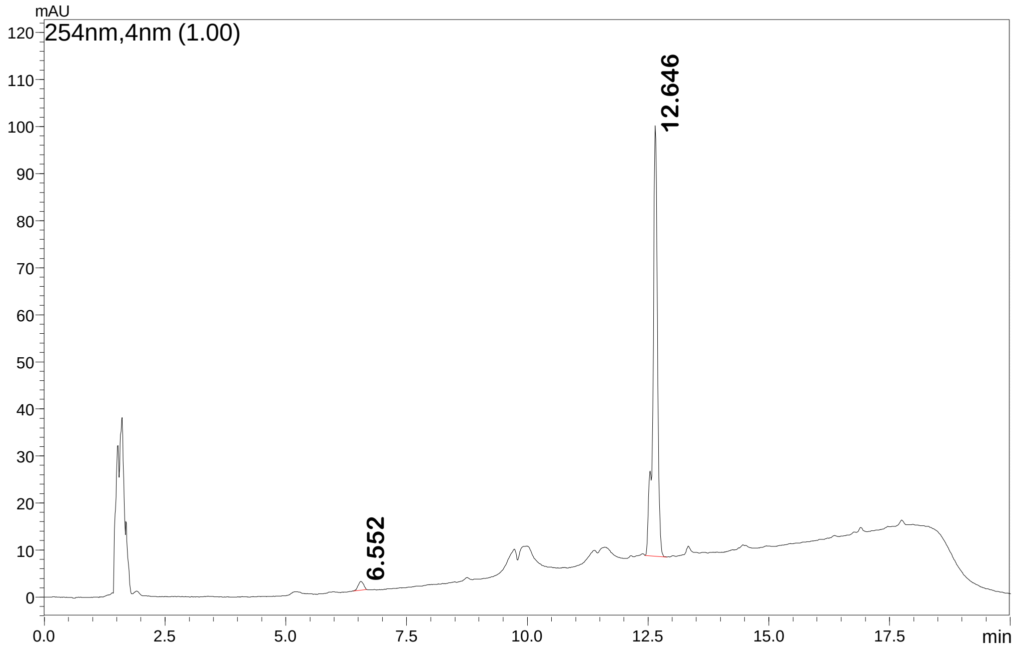

Figure S24. HPLC chromatogram of compound **37**; purity 97.6%.

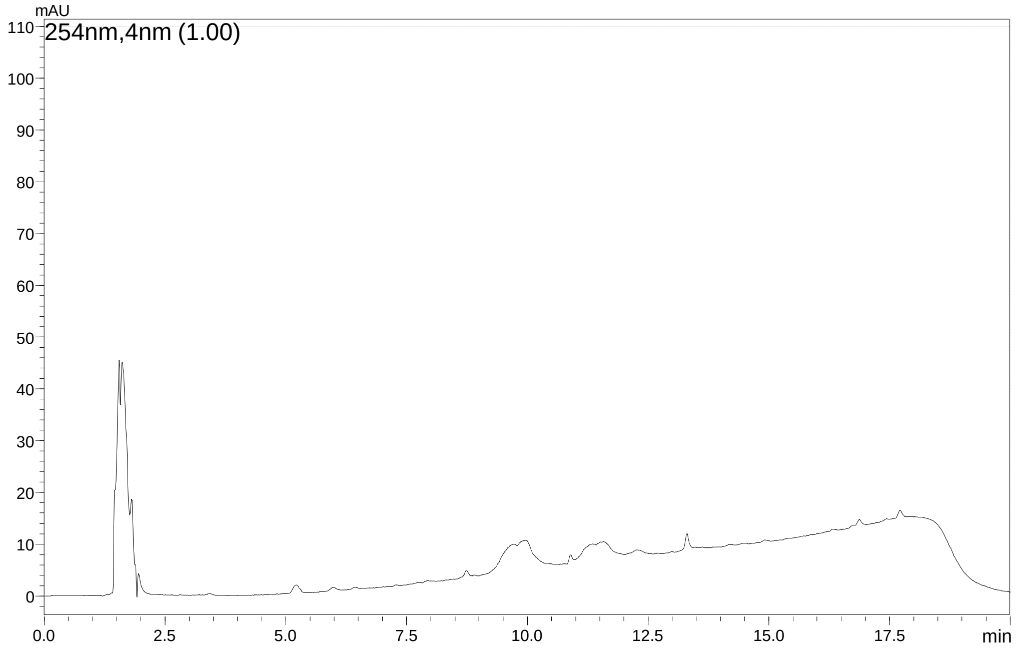

Figure S25. HPLC chromatogram of DMSO.
